# Autism spectrum disorder-specific changes in white matter connectome edge density based on functionally defined nodes

**DOI:** 10.3389/fnins.2023.1285396

**Published:** 2023-11-23

**Authors:** Clara F. Weber, Evelyn M. R. Lake, Stefan P. Haider, Ali Mozayan, Pratheek S. Bobba, Pratik Mukherjee, Dustin Scheinost, Robert T. Constable, Laura Ment, Seyedmehdi Payabvash

**Affiliations:** ^1^Yale University School of Medicine, Department of Radiology and Biomedical Imaging, New Haven, CT, United States; ^2^Social Neuroscience Lab, Department of Psychiatry and Psychotherapy, Lübeck University, Lübeck, Germany; ^3^Center of Brain, Behavior and Metabolism (CBBM), Lübeck University, Lübeck, Germany; ^4^Department of Otorhinolaryngology, Ludwig-Maximilians-University Munich, Munich, Germany; ^5^Department of Radiology and Biomedical Imaging, University of California, San Francisco, San Francisco, CA, United States; ^6^Yale University School of Medicine, Department of Pediatrics and Neurology, New Haven, CT, United States

**Keywords:** autism spectrum disorder, neurodevelopment, DTI, connectome, microstructure

## Abstract

**Introduction:**

Autism spectrum disorder (ASD) is associated with both functional and microstructural connectome disruptions. We deployed a novel methodology using functionally defined nodes to guide white matter (WM) tractography and identify ASD-related microstructural connectome changes across the lifespan.

**Methods:**

We used diffusion tensor imaging and clinical data from four studies in the national database for autism research (NDAR) including 155 infants, 102 toddlers, 230 adolescents, and 96 young adults – of whom 264 (45%) were diagnosed with ASD. We applied cortical nodes from a prior fMRI study identifying regions related to symptom severity scores and used these seeds to construct WM fiber tracts as connectome Edge Density (ED) maps. Resulting ED maps were assessed for between-group differences using voxel-wise and tract-based analysis. We then examined the association of ASD diagnosis with ED driven from functional nodes generated from different sensitivity thresholds.

**Results:**

In ED derived from functionally guided tractography, we identified ASD-related changes in infants (*p*_*FDR*_ ≤ 0.001–0.483). Overall, more wide-spread ASD-related differences were detectable in ED based on functional nodes with positive symptom correlation than negative correlation to ASD, and stricter thresholds for functional nodes resulted in stronger correlation with ASD among infants (*z* = −6.413 to 6.666, *p_*FDR*_* ≤ 0.001–0.968). Voxel-wise analysis revealed wide-spread ED reductions in central WM tracts of toddlers, adolescents, and adults.

**Discussion:**

We detected early changes of aberrant WM development in infants developing ASD when generating microstructural connectome ED map with cortical nodes defined by functional imaging. These were not evident when applying structurally defined nodes, suggesting that functionally guided DTI-based tractography can help identify early ASD-related WM disruptions between cortical regions exhibiting abnormal connectivity patterns later in life. Furthermore, our results suggest a benefit of involving functionally informed nodes in diffusion imaging-based probabilistic tractography, and underline that different age cohorts can benefit from age- and brain development-adapted image processing protocols.

## 1 Introduction

In the United States, one in thirty-six (2.8%) 8-year-old children have been diagnosed with autism spectrum disorder (ASD) (CDC, 2023). Intense efforts to study ASD etiology and pathophysiology have identified numerous etiological contributors, including genetic factors (Jacquemont et al., 2014; Grove et al., 2019) and morphological correlates (Li et al., 2017; Figueiredo et al., 2020), with converging evidence for connectome disruptions playing a central role in the pathogenesis of ASD (Assaf et al., 2010; Hong et al., 2019; Benkarim et al., 2021; Weber et al., 2022).

ASD typically manifests in difficulties in communication and social interaction, as well as through repetitive behavior patterns. These three core symptoms build the basis for the Autism diagnostic observation schedule (ADOS) (Lord et al., 2000), the gold-standard for diagnostic interviewing in ASD (Levy et al., 2009). Among individuals on the autism spectrum, symptom manifestation, onset and severity are largely heterogeneous. Timely diagnosis and therapeutic intervention are crucial for optimal support of autistic individuals, but high heterogeneity in symptom manifestation and severity can impede recognition of early symptoms and access to appropriate resources. While ASD is commonly first diagnosed in childhood and at school age, new diagnoses occur throughout the lifespan (Hume et al., 2021). Depending on a subject’s sex, socioeconomic resources, individual symptom profile and their ability to camouflage symptoms, diagnosis may be significantly delayed (Howlin et al., 2004; Huang et al., 2021; Hume et al., 2021), thus impeding early intervention. This underlines the demand for a better understanding of the underlying pathophysiology as well as for reliable non-invasive biomarkers for ASD.

**FIGURE 1:**
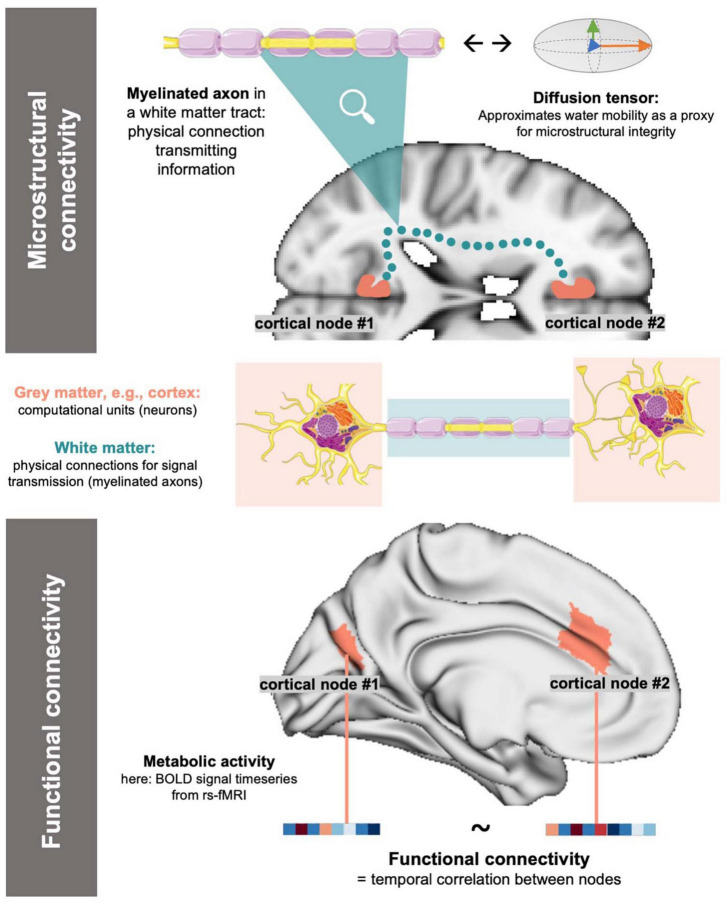
Simplified, conceptual depiction of microstructural and functional connectivity: For functional connectivity, cortical activity is approximated (e.g., as BOLD signals in rs-fMRI) and connectivity is derived as the correlation of activity between nodes. Microstructural connectivity reflects on the physical underpinnings of signal transmission (=WM tracts) by using water diffusivity in a tensor model as a proxy. The above, blue dotted microstructural connection is a conceptual representation of a WM link and does not accurately reflect neuroanatomy. Image sources: Servier Medical Art by Servier, licensed under a Creative Commons Attribution 3.0 Unported License (https://creativecommons.org/licenses/by/3.0/, neuron and axon graph), MNI-152 standard brain template (axial slice) (Mazziotta et al., 2001), standard brain surface mesh plotted via brainspace (https://github.com/MICA-MNI/brainspace/, surface in lower panel) (Fischl, 2012; Vos de Wael et al., 2020).

Magnetic resonance imaging (MRI) offers a unique method to study the human brain *in vivo*. In the most recent efforts to investigate the brain’s connectome, i.e., as the sum of interconnected neuronal populations (Sporns et al., 2005), MRI offers different modalities to study both functional as well as microstructural connectivity between cortical nodes (Sporns et al., 2005; Finn et al., 2015). Functional MRI (fMRI) depicts connectivity as the time-course correlation between energy consumption rates of cortical nodes by leveraging the blood-oxygen-level-dependent signal as a proxy for metabolic activity (Logothetis et al., 2001; Gauthier and Fan, 2019). Microstructural connectivity can be depicted via four-dimensional diffusion-weighted imaging and the subsequent derivation of a diffusion tensor model (diffusion tensor imaging, DTI) (Lenglet, 2015; Tae et al., 2018). DTI enables the description of the microstructural properties of white matter (WM) tracts by reflecting water molecule mobility in their cellular components (Tae et al., 2018). Both techniques image the connection between cortical nodes: fMRI represent synaptic links between nodes (Raichle, 1998; Glover, 2011), while DTI reflects the WM fiber tracts responsible for signal transmission (Lenglet, 2015) (Figure 1).

ASD-related functional connectivity alterations have been identified, pointing toward a mosaic pattern of functional underconnectivity in cortical networks as well as overconnectivity between the cortex and subcortical nuclei (Assaf et al., 2010; Müller et al., 2011; Benkarim et al., 2021). Similarly, microstructural connectivity disruptions have been identified, mostly impacting commissural tracts in the corpus callosum (Cheon et al., 2011; Ameis and Catani, 2015; Weber et al., 2022). The existing evidence of connectome alterations – both functional and microstructural – prompt the effort for multimodal imaging in ASD, to understand the underlying pathophysiological mechanisms and facilitate early recognition (Müller et al., 2011; Li et al., 2017). These connectivity changes show relation to age, specifically, there is evidence for inter-network hyperconnectivity and local hypoconnectivity in ASD children, whereas hypoconnectivity prevails among adults (Uddin et al., 2013a,b; Haghighat et al., 2021). Evidence suggests altered neurodevelopmental processes as potential biomolecular substrates of ASD-associated connectivity changes, including increased neuronal cell count (McFadden and Minshew, 2013; Kana et al., 2014) and columnar density (Buxhoeveden et al., 2006; Hutsler and Casanova, 2016) that impede cortical maturation processes in infancy (Uddin et al., 2013b; Kana et al., 2014; Haghighat et al., 2021). Typically, the first years of life are characterized by rapid cortical maturation and WM development (Deoni et al., 2012; Keehn et al., 2013; Sadeghi et al., 2013; Yu et al., 2020), transitioning into a period of slower growth in early childhood (Mukherjee et al., 2001; Lebel et al., 2008; Lebel and Beaulieu, 2011), and subsequently reaching steady growth levels in adolescence (Mukherjee et al., 2001; Westlye et al., 2010; Yu et al., 2020). Developmental processes and potential atypical deviations can be captured by different MRI modalities: Cortical connectivity alterations are usually studied using functional imaging (Supekar et al., 2013; Li et al., 2017; Morgan et al., 2019), whereas diffusion-weighted or structural imaging is used to detect microstructural changes (Ameis and Catani, 2015; Ismail et al., 2016; Li et al., 2017; Weber et al., 2022). Since ASD-related connectome alterations affect brain maturation differently throughout the lifespan (Ecker et al., 2015; Haghighat et al., 2021), we suggest that ASD-associated developmental abnormalities in certain age groups might benefit from multimodal imaging methods comprising both functional and microstructural information.

In the past years, new methods of MRI processing have emerged that seek to image connectome alterations in ASD, including probabilistic tractography, a method to remodel fibers based on DTI outcomes (Mazziotta et al., 2001; Behrens et al., 2007). From tractography, edge density maps (ED) can be derived that represent the number of WM microstructural connections (edges) between nodes. Traditional DTI-derived metrics such as fractional anisotropy and diffusivity measures reflect on water molecule mobility in single voxels and are therefore restricted in their ability to capture crossing fibers and full-length WM tracts (Seunarine and Alexander, 2014; Tae et al., 2018). ED offers the opportunity to remodel microstructural cortico-cortical connections, therefore allowing to study WM tracts in their full continuity (Behrens et al., 2003, 2007; Seunarine and Alexander, 2014; Owen et al., 2015). Changes in probabilistic ED have been reported in sensory processing and neurodevelopmental disorders, hinting toward potential benefits of including ED in addition to traditional DTI metrics in microstructural imaging studies (Payabvash et al., 2019a,b; Weber et al., 2022).

In a previous study (Weber et al., 2022), we leveraged DTI metrics and probabilistic ED to study the connectome alterations associated with ASD and found changes in adolescents and young adults; however, we were unable to find ASD-associated disruptions in younger cohorts (Weber et al., 2022). In the prior study, we used a generic anatomical atlas to define seed masks in tractography. Based on the increasing evidence for functional connectivity alterations in ASD, we here seek to integrate functional and WM microstructural connectome in our previous approach by using cortical nodes of ASD-related functional changes to guide WM tractography and generate fMRI-informed ED maps of the brain. We utilized multi-centric DTI datasets from four different study cohorts that include different age groups from infancy to adulthood. We employed probabilistic tractography that is, contrary to the preceding study, not based on anatomical cortical areas but rather representative of ASD symptom severity based on prior fMRI studies. We investigated ASD-related changes across different age cohorts using both voxel-wise analysis methods as well as tract-based comparisons.

## 2 Materials and methods

### 2.1 Datasets

In this study, we utilized a dataset of DTI and T_1_-weighted images from the national database of autism research (NDAR), consisting of four different study cohorts that each reflect a different age cohort: (i) *Infants* (A Longitudinal MRI Study of Infants at Risk for Autism (Piven, 2008), median age at imaging: 6 months), (ii) *Toddlers* (Biomarkers of Autism at 12 months (Courchesne, 2007), median age: 32 months), (iii) *Adolescents* (Multimodal Developmental Neurogenetics of Females with ASD (Pelphrey, 2012), median age: 13.1 years), (iv) *Adults* (Atypical late neurodevelopment in autism: A Longitudinal MRI and DTI study (Lainhart, 2007), median age: 19.1 years). For infants, ASD assessment followed at 24 months of age, while all other cohorts were evaluated at the time of imaging. Table 1 provides more detailed demographic information for each cohort. We excluded all subjects lacking ASD diagnosis status or any of the two imaging modalities, as well as subjects with genetic and psychiatric comorbidities. Furthermore, all images underwent visual quality control, and all subjects with failed linear coregistration to a standard brain template were excluded, resulting in a sample size of *n* = 583. A workflow of this process is shown in Supplementary Figure 1.

**TABLE 1:** Demographic information about the four study cohorts investigated.

Age cohort	Original study	*n*	Mean age (SD)	ASD/TDC	Male [*n*, (%)]
Infants	Longitudinal MRI study of infants at risk of autism	155 (27%)	6.68 (0.8) months	34/121	102 (65.8%)
Toddlers	Biomarkers of autism at 12 months	102 (18%)	20.14 (8.33) months	57/45	75 (73.5%)
Adolescents	Multimodal developmental neurogenetics of females with autism	230 (39%)	12.53 (2.95) years	106/124	117 (50.9%)
Young adults	Atypical late neurodevelopment in autism	96 (16%)	19.79 (8.33) years	67/29	95 (99.0%)

ASD, autism spectrum disorder; SD, standard deviation; TDC, typically developing controls.

### 2.2 Image acquisition

The acquisition protocols of study cohorts included:

(i) In the infant cohort, T1-weighted imaging was conducted with a repetition time (TR) of 2400 ms, time to echo (TE) of 3.16 ms, field of view (FOV) of 256, matrix size 224 × 256, and slice thickness 1 mm, diffusion weighted images were acquired in 26 variable *b*-values between 50 and 1000 s/mm2 increasing by 200 s/mm2 at each scan (25 gradient directions and one non-weighted image with *b* = 0 s/mm2) image on 3T Siemens Tim Trio, with TR = 12,800–13,300 ms, TE = 102 ms, FOV 190, matrix size 190 × 190, and slice thickness of 2 mm;
(ii) Toddlers’ T1-weighted imaging was acquired with TR = 6500 ms, TE = 2.8 ms, FOV = 240, matrix size 96 × 96, slice thickness 1.2 mm, DTI included 51 images with *b* = 1000 s/mm2 and one non-weighted *b* = 0 s/mm2 image acquired on 1.5 T GE Signa HDxt, TR = 13200 ms, TE = 80.6 ms, FOV 240, matrix size 96 × 96, and slice thickness 2.5 mm;
(iii) Adolescents’ T1-weighted imaging was acquired with TR = 5300 ms, TE = 3.3 ms, FOV 350, matrix size 192 × 192, slice thickness = 1 mm, DTI included 46 images with *b* = 1000 s/mm2 and one non-weighted b = 0 s/mm2 image acquired on 3T Siemens Magnetom TrioTim, TR = 13,000 ms, TE = 93 ms, FOV 250, matrix size 192 × 192, and slice thickness 2.5 mm;
(iv) Adults’ T1-weighted imaging was acquired with TR = 1800, TE = 1.93, FOV 256, matrix size 256 × 240, slice thickness 1 mm, DTI included 4 repetitions of 12 images with *b* = 1000 s/mm2 and followed by an image with *b* = 0 s/mm2 acquired on 3T Siemens Magnetom TrioTim, with TR = 7000 ms, TE = 91 ms, FOV = 256, matrix size 128 × 128, and slice thickness 2.5 mm.

### 2.3 Data preprocessing

DTI data and T_1_-weighted data were converted to Nifti format and preprocessed using FSL brain extraction in the FMRIB Software Library (FSL)^1^ (Smith, 2002; Smith et al., 2004; Li et al., 2016), which included eddy current correction and brain extraction. We then applied FSL’s diffusion tensor fitting tool (DTIFIT) (Smith et al., 2004) on all DTI data to retrieve mean (MD), axial (AD) and radial diffusivity (RD) as well as fractional anisotropy (FA) maps. These metrics correspond to overall water molecule diffusivity (MD), mobility along (AD) and perpendicular (RD) to a WM tract as well as to directional dependency (FA) and therefore can be appreciated as a proxy of WM integrity and maturation (Lenglet, 2015; Tae et al., 2018). We then corrected for crossing fibers using Bayesian estimation of crossing fibers (BEDPOSTX) in FSL (Behrens et al., 2007; Woolrich et al., 2009), which employs a ball- and stick model to depict water mobility in each voxel.

### 2.4 Seed identification and probabilistic tractography

**FIGURE 2:**
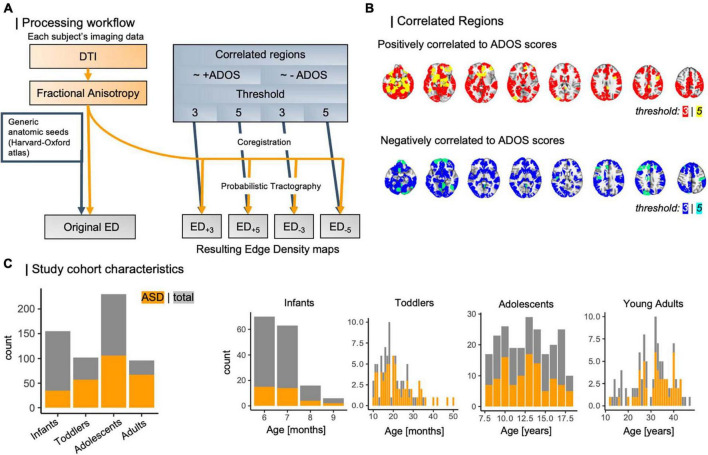
**(A)** Workflow of ED computation for different threshold levels. Based on external data from Lake et al. (2019), which leveraged CPM to analyze brain nodes correlating to ASD symptom severity, we determined functionally informed masks **(B)** to guide tractography. **(C)** Study cohort compositions regarding case/control ratio and age distribution. ADOS, autism diagnostic observation schedule; ASD, autism spectrum disorder; CPM, connectome predictive modeling; DTI, diffusion tensor imaging; ED, edge density.

Subsequently, we linearly coregistered regions of interest (ROIs) to each individual’s native FA space using FSL’s linear transformation tool (Smith et al., 2004). In a previous approach (Weber et al., 2022), we used the Harvard-Oxford subcortical and cortical structural atlases (Mazziotta et al., 2001; Frazier et al., 2005; Desikan et al., 2006; Makris et al., 2006; Goldstein et al., 2007) for identification of cortical nodes. A full list of all regions used is given in Supplementary Table 1. We used these masks as seeds in probabilistic tractography using FSL PROBTRACKX (Smith et al., 2004; Woolrich et al., 2009; Wu et al., 2018) to build each individual’s edge density (ED) maps (Figure 2A) (Owen et al., 2015; Payabvash et al., 2019a; Weber et al., 2022). We built ED maps from cortical nodes defined by functional imaging (CNFI). From previous work by Lake et al. (2019), we then identified cortical regions that correlate to ASD symptom severity as assessed using the Autism diagnostic observation schedule (ADOS) (Lord et al., 2000). Briefly, this work applied connectome-based predictive modeling (CPM) (Shen et al., 2017), which leverages rs-fMRI derived functional connectivity matrices to model individual connectomes in a leave-one-out framework. Then, the number of edges between cortical parcels were determined. These regions were outlined in the Shen atlas (Lee et al., 2014) and subset for different sensitivity thresholds (Figure 2B). In brief, composite networks, i.e., overarching networks comprising sub-scale interactions, correlating to ADOS scores have been identified using CPM, and thresholds are referring to a node’s contribution to composite networks, where most lenient thresholds include all edges appearing at least once in any network and strictest thresholds comprise edges that appear on all sub-scales (Shen et al., 2017; Lake et al., 2019). Here, we utilize four CNFI ROI-masks: two threshold levels (3, 5) per positively and negatively ADOS-correlated regions each. All four were transformed to each subject’s space using linear coregistration. We then employed these masks as seeds in four separate runs of probabilistic tractography, retrieving four new ED maps per individual that are derived from ASD-specific functional changes.

In the following, we will refer to each of these masks by their seed masks of origin, more specifically by direction of correlation to ADOS scores and threshold applied in CPM: ED_+3_ for positively correlated nodes at a threshold of 3, ED_+5_ for positively correlated nodes at a threshold of 5, and vice versa for negatively correlated nodes at thresholds 3 and 5, ED_–3_, and ED_–5_. A full overview of all processing steps is summarized in Figure 2A.

### 2.5 Voxel-wise tract based spatial statistics (TBSS)

For voxel-wise comparison, we applied the FSL’s tract based spatial statistics (TBSS) protocol (Smith et al., 2006). We assessed group level-difference after controlling for age and sex in a linear model using non-parametric permutation-based testing in *n* = 5000 permutations and threshold-free cluster enhancement (Smith and Nichols, 2009) to correct for multiple comparisons across brain space. Each study cohort was analyzed separately to address the confounding influence of differing image acquisition parameters and age group-specific morphological characteristics.

### 2.6 Statistical analysis

We extracted mean values within each of the major WM tracts specified in the John Hopkins University (JHU) atlas (van Zijl et al., 2005), a full list of which is given in Supplementary Table 1. We assessed group differences in tract-based mean ED values using two-sided unpaired *t*-tests between diagnosis groups. To ensure robustness of our results, we repeated all tract-wise analysis after shuffling diagnosis labels in 5000 permutations. In a confirmatory second approach, we determined Spearman’s correlation between ED values in every tract and ASD diagnosis status. Subsequently, we tested whether the threshold applied in CPM correlates with higher group differences, i.e., if tractography guided via stricter or less lenient defined functionally defined nodes leads to higher sensitivity for ASD, using Pearson’s 1898 method of comparing correlation (Pearson and Filon, 1898; Diedenhofen and Diedenhofen, 2016). Briefly, this method compares correlation coefficient between two samples using Fisher’s Z-scores. All *p*-values were corrected for multiple testing using Benjamini and Hochberg’s false discovery rate (FDR) correction (Benjamini and Hochberg, 1995). All statistical analysis were conducted using Python v3.9.7 (van Rossum, 1995) and R v4.3.1 (R Core Team, 2023).

## 3 Results

### 3.1 Study cohort characteristics

In total, we analyzed data from 583 individuals with an age range from 6 months to 50 years, subset into four age-specific cohorts: infants with a mean age of 7 months (median: 7 months), toddlers with a mean age of 20 months (median: 32 months), as well as adolescents and adults, who were on average 13 and 20 years old respectively [median: 13 years (adolescents), 19 years (adults)]. The case-to-control ratio varied between 0.28 and 2.31 (overall: 0.83) and is depicted in Figure 2C along with age distributions across the four study cohorts.

### 3.2 Tract-wise group differences

**FIGURE 3:**
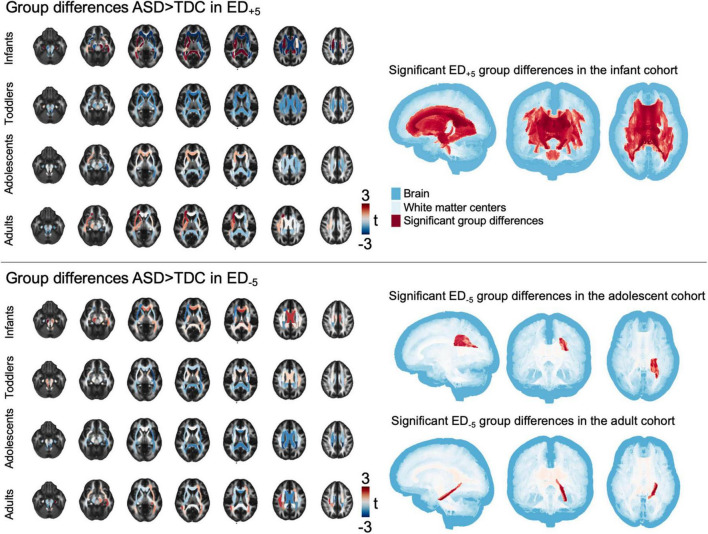
Tract-based results. The figure shows t-statistic in each of the tracts defined in the JHU atlas for each age cohort separately, overlayed on a sample mean FA map. Right panel shows areas of significant group differences (p_FDR_ < 0.05). ASD, autism spectrum disorder; ED, edge density; FA, fractional anisotropy; FDR, false discovery rate; JHU, Johns Hopkins University.

Group differences in tract-based comparison of ED_+5_ revealed wide-spread ASD-related reductions in infants, that reached statistical significance throughout the central and periventricular WM tracts (Figure 3). Toddlers’ t-statistics showed a mosaic pattern of negligible group differences, and adolescents leaned toward widespread reductions that were not statistically significant. Among adults, a mosaic pattern of both ASD-related increases and decreases in *t*-values could be observed, with increases being mainly localized in the left anterior tracts and decreases focused on the right posterior areas. In ED_–5_, mosaic patterns of slight increases and decreases could be found across all age-group cohorts, with toddlers and adolescents exhibiting negative t-statistics and patterns of positive group differences in central WM tracts of infants and adults. Notably, these changes did not reach statistical significance among infants and toddler cohorts (*p_*FDR*_* = 0.143–0.48 in infants and *p_*FDR*_* = in toddlers, detailed *p*-values for each tract are listed in Supplementary Tables 2A,B), whereas in adolescents, lower ED_–5_ were associated with ASD (*t* = −3.346, *p_*FDR*_* = 0.047) and in adults, ED_–5_ in the left hippocampal aspect of the cingulum showed positive association to ASD (*t* = 3.744, *p_*FDR*_* = 0.016) (Figure 3).

In ED based on more lenient thresholds (i.e., ED_–3_ and ED_+3_), we observed a similar pattern, although most t-statistics were leaning toward decreases in ASD, with widespread significant reductions among the adolescent age-group (Supplementary Figure 2). In permutation tests, results showed consistency across shuffled labels (Supplementary Table 5). Confirmatory analysis using Spearman’s rank correlation test showed similar, but smaller effects. A full list of all statistics is given in Supplementary Tables 2A–D. In comparison to our current approach employing tractography based on functional defined nodes, Supplementary Figure 3 and Supplementary Table 4 show results from previous work where we used anatomical nodes to guide tractography (Weber et al., 2022).

### 3.3 Differential impact of different thresholds in seed masks

**FIGURE 4:**
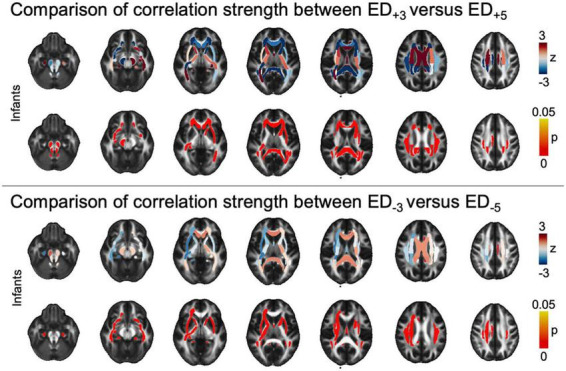
Tract-based comparison of correlation strength between ED based on different threshold leniencies. The figure shows z- and p_FDR_ values in each of the tracts defined in the JHU atlas in the infant cohort, overlayed on a sample mean FA map. Tract-based values are given in Supplementary Table 3A. ASD, autism spectrum disorder; ED, edge density; FA, fractional anisotropy; FDR, false discovery rate; JHU, Johns Hopkins University.

At a tract-level analysis, when we compared ED_+3_ and ED_+5_ in each group respectively, we found that higher threshold masks (i.e., more selective masks derived from functional imaging) used to guide tractography had stronger positive association with ASD diagnosis in the infant cohort in central callosal and periventricular WM tracts, as well as the brainstem (Figure 4). Similarly, when comparing ED_–3_ and ED_–5_, higher thresholds were associated with higher correlation to ASD diagnosis in infants (Figure 4). There was no significant difference in correlation strength between threshold levels in older age cohorts (Supplementary Tables 3A–D).

### 3.4 Voxel-wise group differences

**FIGURE 5:**
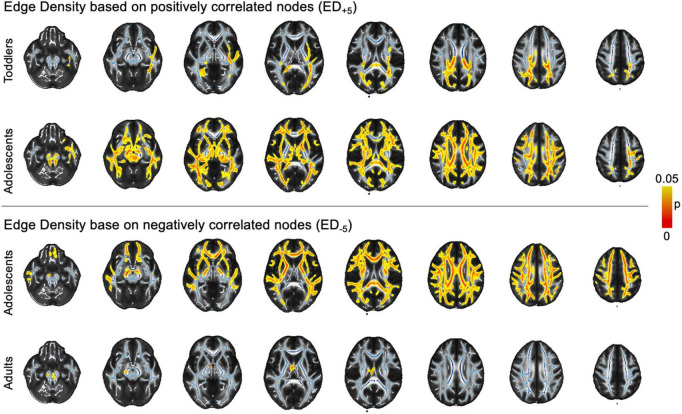
Voxel-wise findings. The image depicts statistically significant values (*p* < 0.05) as determined in permutation testing from TBSS on the standard FA skeleton (blue) on a mean FA template. ASD, autism spectrum disorder; ED, edge density; FA, fractional anisotropy; FDR, false discovery rate; JHU, Johns Hopkins University.ct-based comparison of correlation strength between ED based on different threshold leniencies. The figure shows z- and p_FDR_ values in each of the tracts defined in the JHU atlas in the infant cohort, overlayed on a sample mean FA map. Tract-based values are given in Supplementary Table 3A. ASD, autism spectrum disorder; ED, edge density; FA, fractional anisotropy; FDR, false discovery rate; JHU, Johns Hopkins University.

In voxel-wise analysis using a general linear model controlling for age, we found ASD-related ED_+5_ reductions in adolescents and toddlers. Changes were widespread in adolescents, but less pronounced and more focused on posterior WM tracts in toddlers. In ED_–5_, we found changes in adolescents and adults that both revealed ubiquitous ED reductions (Figure 5). However, there were no significant differences related to ASD diagnosis in any of the age groups when using more lenient ED_+3_ and ED_–3_ thresholds.

## 4 Discussion

Connectome alterations have been established as a key neuroimaging correlate of ASD. Both functional connectivity and microstructural disruptions have been predominantly identified among adolescent and adult ASD cohorts (Müller et al., 2011; Li et al., 2017; Figueiredo et al., 2020). In a previous study leveraging DTI-derived metrics, we could not find group differences in younger cohorts, more specifically in infants and toddlers (Weber et al., 2022). This raises the question if WM connectivity alterations in ASD appear later in life, or if they are not detectable in younger children. In this study, we advanced conventional tractography analyses by integrating findings from functional imaging studies and specifying regions that correlate to ADOS symptom severity to guide tractography. Using this approach, we found ASD-related reductions in edge density based on cortical nodes defined by functional imaging. Notably, we were able to identify changes in infants that were not detectable in our prior study that employed conventional ED based on anatomical nodes. These ED changes were appreciable in most central callosal and periventricular WM tracts. Comparing correlation coefficients between ED maps based on different thresholds, we found higher correlation to ASD diagnosis status in stricter thresholds, i.e., when guiding tractography through nodes with highest sensitivity for ASD. In contrast, in adolescent and adult cohorts, changes were appreciable in ED based on more lenient thresholds, consistent with prior findings where changes were found in ED derived from guiding tractography through generic anatomical nodes, therefore having very low sensitivity for cortical changes in ASD. While we were unable to reproduce infants’ group differences in voxel-wise analysis, we were able to identify changes in the toddler and adolescent cohort, revealing widespread ASD-associated reductions.

Cortical nodes for tractography were derived from a study employing CPM to find composite networks correlating with ASD symptom severity (Lake et al., 2019). First, we used regions correlating positively to ADOS scores, hence pointing out cortical areas that exhibit connectivity changes with increasing symptom severity. These nodes were localized in inferior temporal lobes bilaterally, as well as the right frontal lobe. Additionally, we used nodes that are inversely correlated to symptom severity, which were situated primarily in bilateral frontal and occipital cortical aspects. These nodes differ from our previous approach, where we used a generic anatomic atlas enclosing most of the cortex and subcortical nuclei, a full list of which is given in Supplementary Table 1 (Desikan et al., 2006). More lenient thresholds applied in CPM will result in inclusion of more cortical areas, hence, the overlap between our seed mask at threshold 3 overlaps more with the anatomical nodes from our previous approach than at a stricter threshold. Consequently, tractography will build more selective ED maps between regions of interest that were derived at a threshold of 5.

We found most changes when analyzing ED based on functional nodes that correlate positively to ADOS symptom severity scores, i.e., WM disruptions could be imaged by guiding tractography through cortical parcels that are associated with high symptom severity. These ED maps capture connections between cortical parcels that are functionally impacted by ASD, thus, ED disruptions here suggest WM disconnectivity as a microstructural underpinning of cortical changes. Of note, this effect is observed in pediatric cohorts, whereas cortical nodes were derived from an adolescent cohort (Di Martino et al., 2014; Martino et al., 2017; Lake et al., 2019): we guided tractography in younger children based on cortical alterations that were described later in life (Lake et al., 2019). We were able to detect WM disruptions between these parcels in infants, hence hinting toward shared network alterations across age groups. These changes are apparent as WM disruptions in infants, and potentially propagate to more wide-spread changes in adolescents and adults, as these age groups reveal wide-spread changes even in less selective tractography (Weber et al., 2022). Contrarily, ED based on cortical nodes that are inversely correlated to ADOS scores did not reveal significant changes in pediatric cohorts, but in adolescents and adults. Given the similarity of these results to previous findings from probabilistic tractography based on anatomical nodes, i.e., cortical regions that were not specific to ASD symptom severity, we suggest no further benefit of guiding tractography through nodes that are inversely correlated to ADOS scores.

ASD-related disruptions in adults and adolescents were detectable in our previous study using fractional anisotropy as a traditional DTI-derived metric, and ED based on anatomical nodes, whereas we could not detect changes using functionally guided ED. We hypothesize that ASD-associated microstructural disintegrity is higher in adolescents and adults, hence detectable with less specific methodology (Travers et al., 2012; Ameis and Catani, 2015). Potentially, these larger scale changes camouflage alterations in more ASD-specific, functionally guided tractography. Thus, connectivity alterations in adolescents and older subjects appear more pronounced, whereas ASD alterations in younger children are more likely to be masked by lenient, i.e., non-functionally guided tractography.

Our findings further underline the conceptual link between functional imaging of cortical parcels, and microstructural, diffusion-weighted imaging of the WM tracts connecting those nodes. Consistent with previous evidence for functional underconnectivity (Assaf et al., 2010; Müller et al., 2011), our findings highlight shared WM disruptions in ASD that are apparent in adolescents and adults, and can be appreciated in infants when combining functional and diffusion-weighted imaging.

While we found ASD-related ED decreases on a tract-based level in infants, we could find effects in toddlers using voxel-wise analysis, but not on a tract level. Infants’ and toddlers’ brains are in distinct developmental stages. Brains of infants and young children differ largely from adolescents and adults, as they are still in an earlier stage of development. These differences include higher number of neurons and lower number of axonal connections (Waller et al., 2017; Payabvash et al., 2019b; Yu et al., 2020). Within the first years of life, WM matures, i.e., axonal connections are formed and myelination increases (Barnea-Goraly et al., 2005; Swanson and Hazlett, 2019; Changeux et al., 2021). Altogether, these connections form the basis for fast and efficient signal transmission across the cortex and to subcortical nuclei (Oligschläger et al., 2017). In diffusion imaging, these processes correlate to an increase in fractional anisotropy and axial diffusivity, and a decrease in mean and radial diffusivity (Wakana et al., 2007; Lenglet, 2015). There is converging evidence for abnormal brain maturation in ASD, specifically impaired WM maturation in ASD (Ameis and Catani, 2015; Aoki et al., 2017; Payabvash et al., 2019b), as well as neocortical differentiation (Hutsler and Casanova, 2016; Haghighat et al., 2021) and atypical axonal growth (McFadden and Minshew, 2013; Zikopoulos and Barbas, 2013), In this study, we identified reduced WM integrity in bilateral central callosal and anterior periventricular fiber bundles on a tract-based level in infants, suggesting reduced connectivity especially between frontal lobes. These findings align with previous findings showing abnormal connectivity involving the frontal cortical nodes (Assaf et al., 2010; Kumar et al., 2010; Cheon et al., 2011; Poustka et al., 2012; Li et al., 2020). Of note, we observed a different pattern in toddlers, where significant reductions in posterior tracts were observable in voxel-wise, but not in tract-based analysis. Spatially, these changes overlap with findings from previous studies where microstructural integrity in adolescents and adults were identified in posterior callosal tracts (Noriuchi et al., 1362; Ouyang et al., 2016). While both pediatric cohorts exhibit reductions, it is remarkable that decreases are mostly localized in frontal tracts in infants, whereas they are mostly detectable in posterior tracts of toddlers. This discrepancy, combined with previous findings about frontal connectivity abruptions in infancy and posterior microstructural disintegrity in children and adults, suggests a differential impact of ASD on axonal maturation across age groups. Specifically, variable group differences between age groups indicate that the location and overall susceptibility of white matter development depends on a subject’s age.

Additionally, in infants and toddlers, we found changes at the tract- and voxel-wise level respectively. Due to the rapidly adapting brain maturation processes in early childhood, specifically, slower maturation in toddlerhood as compared to infancy (Uddin et al., 2013b; Yu et al., 2020; Haghighat et al., 2021), group differences are potentially present at different levels, appearing across tracts in infants, and being constricted to focal changes within the centers of WM tracts in toddlers. Additionally, microscale maturation processes, i.e., white-gray-matter boundary maturation, neuronal migration and columnar differentiation might be distorting imaging findings (Mukherjee et al., 2001; Lebel and Beaulieu, 2011; Hutsler and Casanova, 2016; Thompson et al., 2020). Potentially, the study cohort composition comprising different sample sizes might have influenced our findings, hence underlining the importance of ensuring the reproducibility of our results upon wider availability of pediatric imaging datasets.

The main strength of our study is the utilization of a large multimodal imaging dataset from four different studies retrieved from a data repository. We circumvented a site-related distortion of our results by analyzing each cohort on its own. Since all studies were acquired separately from each other, technical differences hinder the comparability between studies. Additionally, the case-to-control ratio varies between groups, with the lowest ratio of 0.28 (34 ASD/121 TDC) in infants and 2.31 (67 ASD/29 TDC) in the adult cohort, which we accounted for using a permutation analysis shuffling group labels and showing robustness of our results. While we aimed to include as many as data as available for this study, we acknowledge this limitation and aim to test reproducibility of our findings upon availability of respective imaging data. Similarly, functional nodes of ASD-related changes were retrieved from another study that did not incorporate pediatric subjects. These subjects were separate from our study cohorts; hence, functional alterations are potentially different between the groups. We seek to combine subject-specific functional connectivity changes with DTI-based tractography upon availability of such data on a larger scale. Additionally, we recognize the low percentage of female participants in the adult cohort. The clinical presentation of individuals on the autism spectrum differs remarkably between males and females (Jacquemont et al., 2014; Alaerts et al., 2016; Beggiato et al., 2017), and there is evidence for differential genetic impact between the sexes. We acknowledge that the generalizability of our results is limited for the adult cohort due to the low number of females involved. Upon availability of further, large-scale, high quality and balanced data sets, the reproducibility of our findings ought to be validated. These data can additionally be leveraged to build representative artificial intelligence algorithms focusing on diagnosis and prognosis prediction (Mofatteh, 2021).

## 5 Conclusion

In a large, multi-centric study involving individuals on the autism spectrum and neurotypical controls, we identified changes in ED that were detected by guiding probabilistic tractography through functionally defined nodes. In a previous study using ED based on anatomical nodes, no WM microstructural differences could be appreciated in pediatric cohorts. In infants, we found widespread reductions in bilateral central callosal and periventricular WM on a tract-based level, and toddlers showed significant reductions in voxel-wise analysis that were widespread across posterior tracts. Stricter thresholds for determining seeds for tractography were associated with higher correlation to ASD diagnosis status in infants. Our findings point toward common axes of microstructural disruptions across age groups that are present between cortical nodes correlating with ASD and can be captured using DTI-based tractography. Our results highlight the importance of multimodal imaging in investigating imaging correlates of ASD. Both cross-sectional and longitudinal data sets are required to ensure the generalizability of our results to a broader collective.

## Data availability statement

The original contributions presented in this study are included in this article/Supplementary material, further inquiries can be directed to the corresponding author.

## Ethics statement

Ethical approval was not required for the study involving humans in accordance with the local legislation and institutional requirements. Written informed consent to participate in this study was not required from the participants or the participants’ legal guardians/next of kin in accordance with the national legislation and the institutional requirements.

## Author contributions

CW: Data curation, Formal analysis, Investigation, Methodology, Software, Visualization, Writing – original draft, Writing – review and editing. EL: Conceptualization, Methodology, Writing – review and editing. SH: Writing – review and editing. PB: Writing – review and editing. PM: Writing – review and editing. DS: Writing – review and editing. RC: Writing – review and editing. LM: Writing – review and editing. SP: Conceptualization, Formal analysis, Funding acquisition, Methodology, Project administration, Writing – original draft, Writing – review and editing.

## Footnotes

1
https://fsl.fmrib.ox.ac.uk
