# Supplementary material for: Autism spectrum disorder-specific changes in white matter connectome edge density based on functionally defined nodes
Source: Front Neurosci. 2023 Nov 23;17:1285396. doi: 10.3389/fnins.2023.1285396 (PMC10702224; doi:10.3389/fnins.2023.1285396)
Supplement: Supplementary file 1 [file Data_Sheet_1.docx]

Supplementary Material

# Supplementary Figures

**
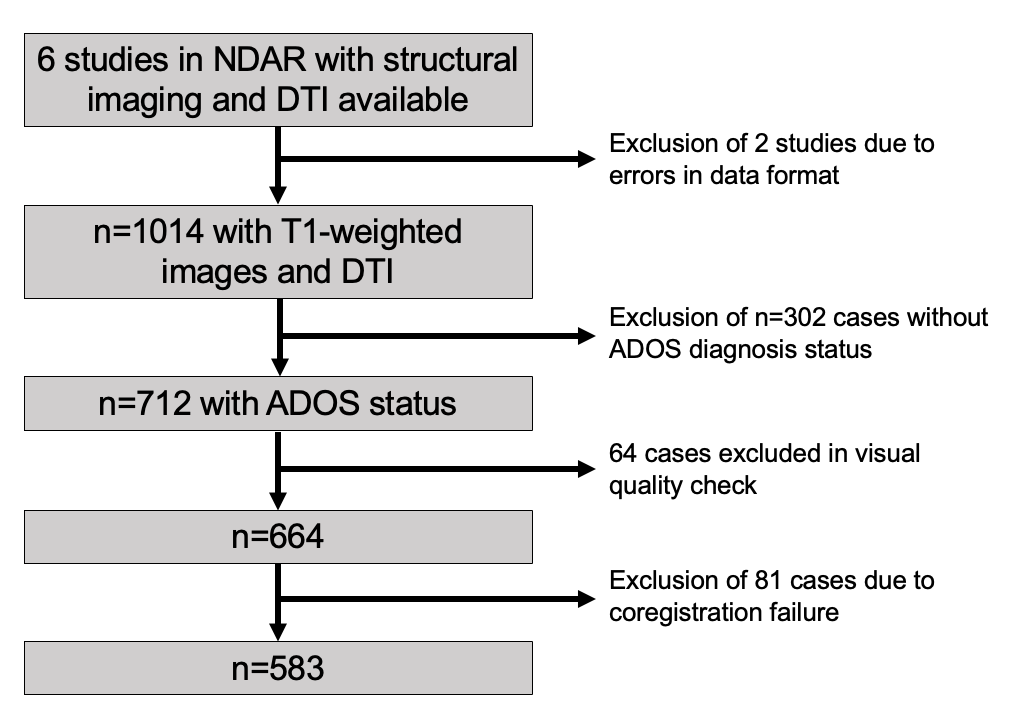
**

**Supplemental Figure 1:** Study cohort composition: case exclusion workflow. *Abbreviations: ASD = autism spectrum disorder, ADOS = autism diagnostic observation schedule, DTI = diffusion tensor imaging, NDAR = national database for autism research*


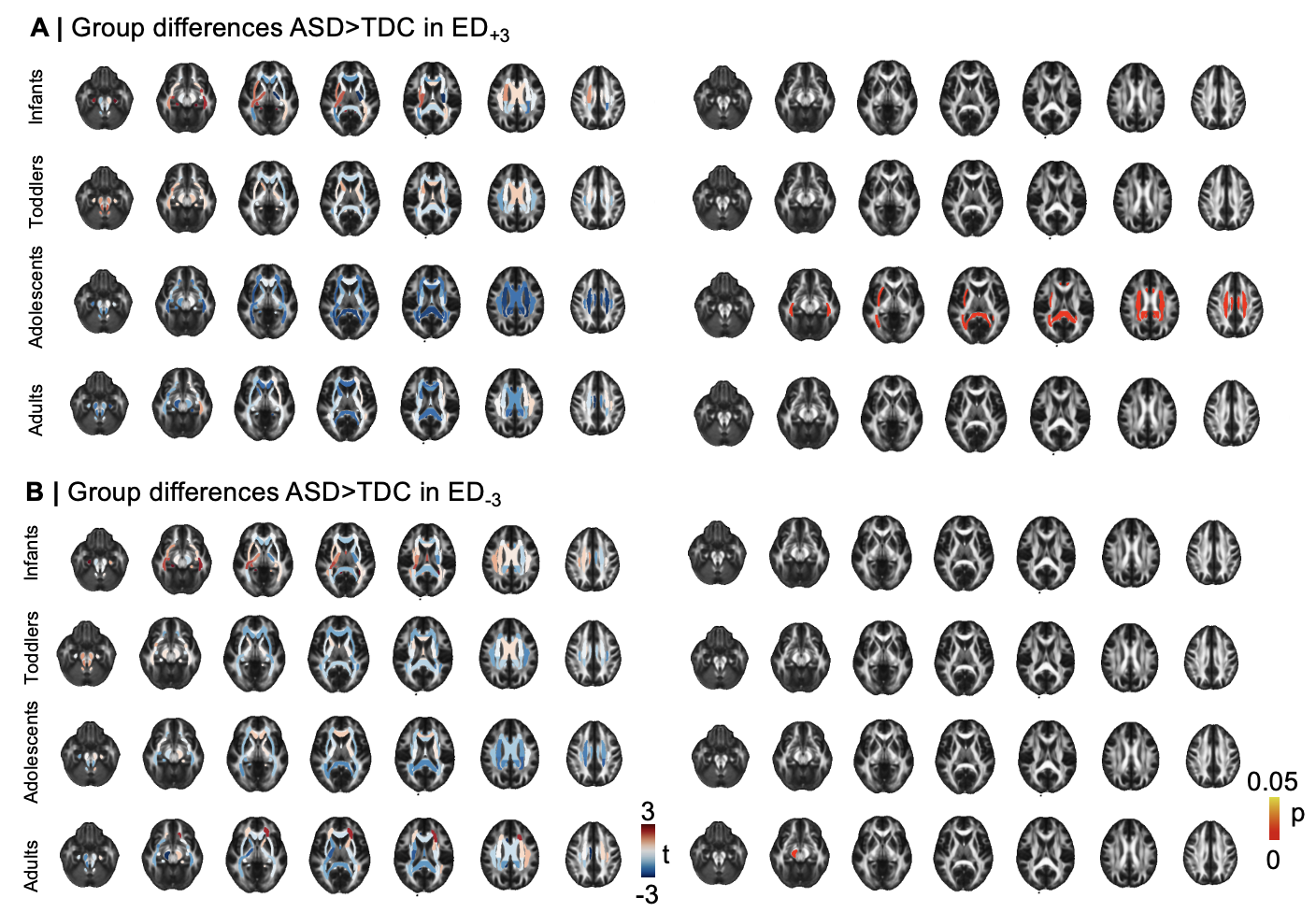


**Supplemental figure 2:** Tract-based results for ED_+3_ and ED_-3._ The figure shows t-statistic and p-values in each of the tracts defined in the JHU atlas for each age cohort separately, overlayed on a sample mean FA map. *Abbreviations: ASD = autism spectrum disorder, ED = edge density, FA = fractional anisotropy, JHU = Johns Hopkins University*

**
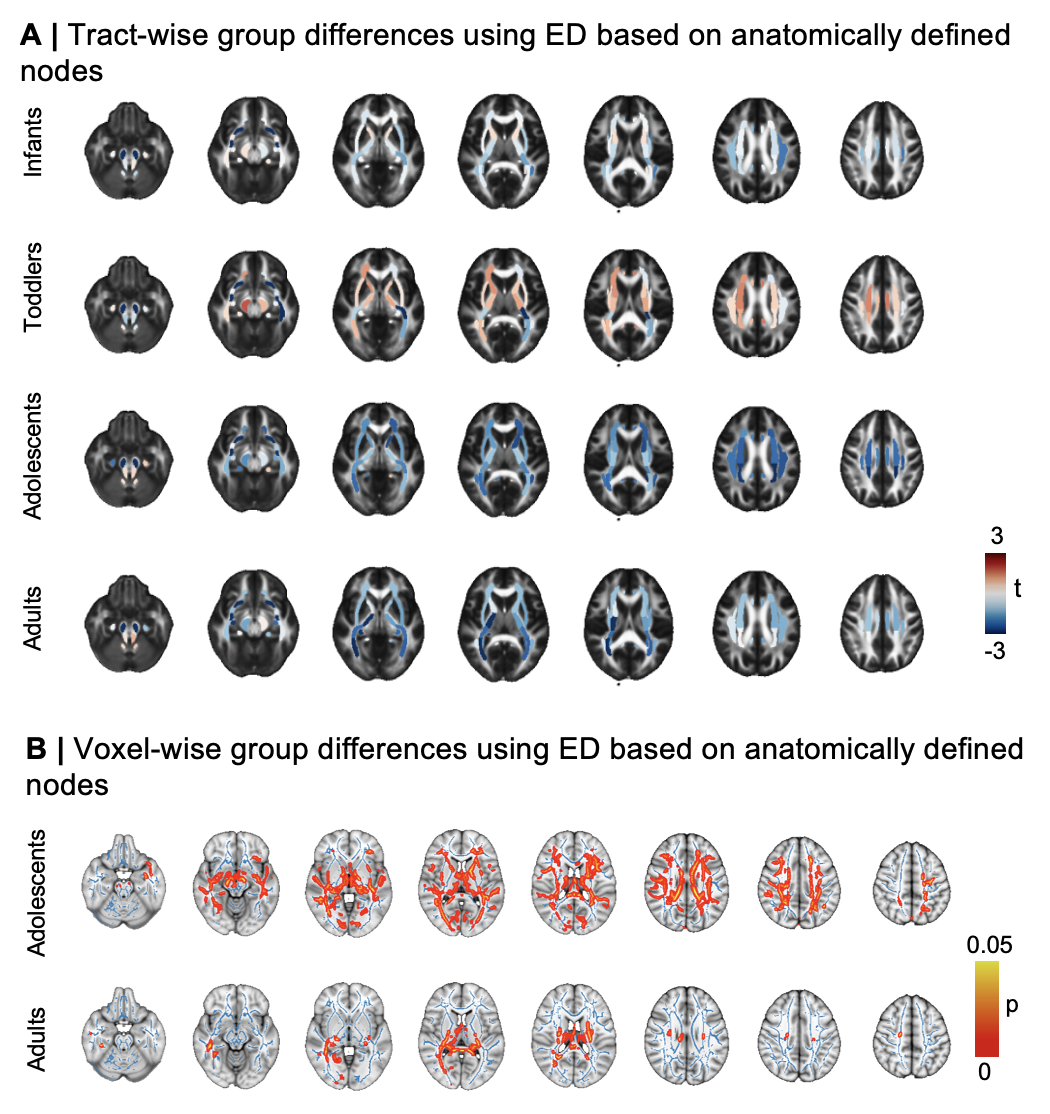
**

**Supplemental figure 3: A** Tract-wise group differences from previous approach, where we utilized probabilistic tractography based on anatomically defined nodes. Figure shows t-value maps on a sample FA template. **B** Voxel-wise group differences in ED based on anatomically defined nodes, generated using a general linear model controlling for age in permutation-based testing (n=5000 permutations). Significance levels were adjusted for multiple comparisons across space using threshold-free cluster enhancement. Figure shows significant changes (p-value maps) on a sample MNI152 brain template, centers of white matter tracts are outlined in blue (mean FA skeleton). *Abbreviations: ASD = autism spectrum disorder, ED = edge density, FA = fractional anisotropy*

# Supplementary Tables

**Supplemental Table 1:** List of regions of interest derived from the Harvard-Oxford cortical and subcortical region atlases (60–63) as well as the John Hopkins University white matter labels atlas(84,95).

| **Atlas** | **Regions included** (L=Left, R=Right) |
| --- | --- |
| John Hopkins University White Matter Labels Atlas | Middle cerebellar peduncle  Pontine crossing fibres  Genu of corpus callosum  Body of corpus callosum  Splenium of corpus callosum  Fornix (column and body of fornix)  Corticospinal tract L/R  Medial lemniscus L/R  Inferior cerebellar peduncle L/R  Superior cerebellar peduncle L/R  Cerebral peduncle L/R  Anterior limb of internal capsule L/R  Posterior limb of internal capsule L/R  Retrolenticular part of internal capsule L/R  Anterior corona radiata L/R  Superior corona radiata L/R  Posterior corona radiata L/R  Posterior thalamic radiation (include optic radiation) L/R  Sagittal stratum (include inferior longitudinal fasciculus and inferior fronto-occipital fasciculus) L/R  External capsule L/R  Cingulum (cingulate gyrus) L/R  Cingulum (hippocampus) L/R  Fornix (cres) / Stria terminalis L/R  Superior longitudinal fasciculus L/R  Superior fronto-occipital fasciculus L/R  Inferior fronto-occipital fasciculus L/R  Uncinate fasciculus L/R  Tapetum L/R |
| Harvard-Oxford Cortical Atlas | Frontal Pole L/R  Insular Cortex L/R  Superior Frontal Gyrus L/R  Middle Frontal Gyrus L/R  Inferior Frontal Gyrus pars triangularis L/R  Inferior Frontal Gyrus pars opercularis L/R  Precentral Gyrus L/R  Temporal Pole L/R  Superior Temporal Gyrus, anterior division L/R  Superior Temporal Gyrus, posterior division L/R  Middle Temporal Gyrus, anterior division L/R  Middle Temporal Gyrus, posterior division L/R  Middle Temporal Gyrus, temporooccipital part L/R  Inferior Temporal Gyrus, anterior division L/R  Inferior Temporal Gyrus, posterior division L/R  Inferior Temporal Gyrus, temporooccipital part L/R  Postcentral Gyrus L/R  Superior Parietal Lobule L/R  Supramarginal Gyrus, anterior division L/R  Supramarginal Gyrus, posterior division L/R  Angular Gyrus L/R  Lateral Occipital Cortex, superior division L/R  Lateral Occipital Cortex, inferior division L/R  Intracalcarine Cortex L/R  Frontal Medial Cortex L/R  Juxtapositional Lobule Cortex L/R  Subcallosal Cortex L/R  Paracingulate Gyrus L/R  Cingulate Gyrus, anterior division L/R  Cingulate Gyrus, posterior division L/R  Precuneous Cortex L/R  Frontal Orbital Cortex L/R  Parahippocampal Gyrus, anterior division L/R  Parahippocampal Gyrus, posterior division L/R  Lingual Gyrus L/R  Temporal Fusiform Cortex, anterior division L/R  Temporal Fusiform Cortex, posterior division L/R  Temporal Occipital Fusiform Cortex L/R  Occipital Fusiform Gyrus L/R  Frontal Operculum Cortex L/R  Central Opercular Cortex L/R  Parietal Operculum Cortex L/R  Planum Polare L/R  Heschl’s Gyrus (incl. H1 and H2) L/R  Planum Temporale L/R  Supracalcarine Cortex L/R  Occipital Pole L/R |
| Harvard-Oxford Subcortical Atlas | Thalamus L/R  Putamen L/R  Pallidum L/R  Hippocampus L/R  Amygdala L/R  Nucleus Accumbens L/R  Brainstem |

**Supplemental tables 2**: Tract-wise results for each age cohort separately. We assessed group differences in ED based on different thresholds separately using a two-sided unpaired t-test as well as Spearman’s rank correlation test. *Abbreviations: ASD = autism spectrum disorder, ED = edge density, FDR = false discovery rate, L = left, R = right*

| **2.a Infants** | | | | | |
| --- | --- | --- | --- | --- | --- |
|  | tract | t | pFDR | Spearman's Rho | pFDR |
| ED_-3_ | Middle cerebellar peduncle | 0.101 | 0.987 | -0.076 | 0.747 |
| ED_-3_ | Pontine crossing tract | -0.34 | 0.987 | 0.065 | 0.768 |
| ED_-3_ | Genu of corpus callosum | -1.151 | 0.877 | 0.107 | 0.722 |
| ED_-3_ | Body of corpus callosum | -1.365 | 0.789 | 0.095 | 0.723 |
| ED_-3_ | Splenium of corpus callosum | 0.298 | 0.987 | -0.102 | 0.722 |
| ED_-3_ | Fornix (column and body of fornix) | 2.013 | 0.391 | 0.07 | 0.747 |
| ED_-3_ | Corticospinal tract R | -0.709 | 0.877 | 0.052 | 0.834 |
| ED_-3_ | Corticospinal tract L | -0.037 | 0.988 | 0.069 | 0.747 |
| ED_-3_ | Medial lemniscus R | -0.532 | 0.933 | -0.03 | 0.928 |
| ED_-3_ | Medial lemniscus L | -0.394 | 0.987 | -0.004 | 0.997 |
| ED_-3_ | Inferior cerebellar peduncle R | -0.139 | 0.987 | 0.001 | 0.997 |
| ED_-3_ | Inferior cerebellar peduncle L | 0.246 | 0.987 | -0.011 | 0.975 |
| ED_-3_ | Superior cerebellar peduncle R | -0.055 | 0.988 | -0.028 | 0.93 |
| ED_-3_ | Superior cerebellar peduncle L | -0.091 | 0.987 | 0.038 | 0.888 |
| ED_-3_ | Cerebral peduncle R | 0.142 | 0.987 | 0.082 | 0.723 |
| ED_-3_ | Cerebral peduncle L | -0.796 | 0.877 | 0.103 | 0.722 |
| ED_-3_ | Anterior limb of internal capsule R | 0.396 | 0.987 | -0.102 | 0.722 |
| ED_-3_ | Anterior limb of internal capsule L | -0.765 | 0.877 | -0.049 | 0.837 |
| ED_-3_ | Posterior limb of internal capsule R | 1.478 | 0.724 | 0.054 | 0.825 |
| ED_-3_ | Posterior limb of internal capsule L | -1.869 | 0.416 | 0.082 | 0.723 |
| ED_-3_ | Retrolenticular part of internal capsule R | 2.067 | 0.391 | -0.013 | 0.974 |
| ED_-3_ | Retrolenticular part of internal capsule L | 0.292 | 0.987 | 0.054 | 0.825 |
| ED_-3_ | Anterior corona radiata R | -0.176 | 0.987 | <0.001 | 0.997 |
| ED_-3_ | Anterior corona radiata L | -0.015 | 0.988 | 0.011 | 0.975 |
| ED_-3_ | Superior corona radiata R | 0.654 | 0.877 | -0.045 | 0.865 |
| ED_-3_ | Superior corona radiata L | -0.883 | 0.877 | 0.092 | 0.723 |
| ED_-3_ | Posterior corona radiata R | 0.559 | 0.933 | -0.038 | 0.888 |
| ED_-3_ | Posterior corona radiata L | -0.652 | 0.877 | 0.094 | 0.723 |
| ED_-3_ | Posterior thalamic radiation R | -0.662 | 0.877 | 0.091 | 0.723 |
| ED_-3_ | Posterior thalamic radiation L | 0.369 | 0.987 | -0.026 | 0.937 |
| ED_-3_ | Sagittal stratum R | 2.007 | 0.391 | -0.119 | 0.682 |
| ED_-3_ | Sagittal stratum L | 2.51 | 0.233 | 0.072 | 0.747 |
| ED_-3_ | External capsule R | 0.709 | 0.877 | -0.069 | 0.747 |
| ED_-3_ | External capsule L | -0.19 | 0.987 | -0.064 | 0.768 |
| ED_-3_ | Cingulum (cingulate gyrus) R | 1.224 | 0.867 | -0.043 | 0.87 |
| ED_-3_ | Cingulum (cingulate gyrus) L | -1.64 | 0.589 | 0.104 | 0.722 |
| ED_-3_ | Cingulum (hippocampus) R | 2.874 | 0.132 | -0.073 | 0.747 |
| ED_-3_ | Cingulum (hippocampus) L | 1.083 | 0.877 | 0.062 | 0.779 |
| ED_-3_ | Fornix (cres)/Stria terminalis R | 1.971 | 0.391 | 0.029 | 0.928 |
| ED_-3_ | Fornix (cres)/Stria terminalis L | 0.668 | 0.877 | 0.081 | 0.723 |
| ED_-3_ | Superior longitudinal fasciculus R | 1.087 | 0.877 | 0.031 | 0.928 |
| ED_-3_ | Superior longitudinal fasciculus L | 0.214 | 0.987 | 0.018 | 0.971 |
| ED_-3_ | Superior fronto-occipital fasciculus R | 1.023 | 0.877 | -0.134 | 0.682 |
| ED_-3_ | Superior fronto-occipital fasciculus L | -0.72 | 0.877 | 0.054 | 0.825 |
| ED_-3_ | Inferior fronto-occipital fasciculus R | 1.326 | 0.789 | -0.097 | 0.723 |
| ED_-3_ | Inferior fronto-occipital fasciculus L | 0.434 | 0.987 | -0.013 | 0.974 |
| ED_-3_ | Uncinate fasciculus R | 0.976 | 0.877 | -0.150 | 0.682 |
| ED_-3_ | Uncinate fasciculus L | 0.688 | 0.877 | 0.046 | 0.859 |
| ED_-3_ | Tapetum R | 0.638 | 0.877 | 0.054 | 0.825 |
| ED_-3_ | Tapetum L | 2.894 | 0.132 | -0.025 | 0.937 |
| ED_-5_ | Middle cerebellar peduncle | 2.112 | 0.143 | -0.024 | 0.943 |
| ED_-5_ | Pontine crossing tract | 2.48 | 0.143 | -0.132 | 0.682 |
| ED_-5_ | Genu of corpus callosum | 1.514 | 0.267 | 0.097 | 0.723 |
| ED_-5_ | Body of corpus callosum | 0.256 | 0.48 | -0.084 | 0.723 |
| ED_-5_ | Splenium of corpus callosum | 2.166 | 0.143 | -0.088 | 0.723 |
| ED_-5_ | Fornix (column and body of fornix) | -0.053 | 0.489 | -0.077 | 0.747 |
| ED_-5_ | Corticospinal tract R | 3.022 | 0.076 | -0.157 | 0.682 |
| ED_-5_ | Corticospinal tract L | 1.515 | 0.267 | -0.118 | 0.682 |
| ED_-5_ | Medial lemniscus R | 1.338 | 0.327 | -0.030 | 0.928 |
| ED_-5_ | Medial lemniscus L | 0.698 | 0.417 | -0.133 | 0.682 |
| ED_-5_ | Inferior cerebellar peduncle R | 0.097 | 0.481 | 0.007 | 0.995 |
| ED_-5_ | Inferior cerebellar peduncle L | 1.163 | 0.341 | -0.13 | 0.682 |
| ED_-5_ | Superior cerebellar peduncle R | 0.624 | 0.417 | <0.001 | 0.997 |
| ED_-5_ | Superior cerebellar peduncle L | 0.203 | 0.48 | -0.238 | 0.283 |
| ED_-5_ | Cerebral peduncle R | 2.07 | 0.143 | -0.118 | 0.682 |
| ED_-5_ | Cerebral peduncle L | 0.22 | 0.48 | -0.103 | 0.722 |
| ED_-5_ | Anterior limb of internal capsule R | -1.501 | 0.267 | -0.031 | 0.928 |
| ED_-5_ | Anterior limb of internal capsule L | -0.977 | 0.383 | 0.001 | 0.997 |
| ED_-5_ | Posterior limb of internal capsule R | 0.896 | 0.383 | -0.040 | 0.883 |
| ED_-5_ | Posterior limb of internal capsule L | 0.163 | 0.48 | -0.128 | 0.682 |
| ED_-5_ | Retrolenticular part of internal capsule R | -0.881 | 0.383 | 0.141 | 0.682 |
| ED_-5_ | Retrolenticular part of internal capsule L | 0.982 | 0.383 | -0.090 | 0.723 |
| ED_-5_ | Anterior corona radiata R | -2.202 | 0.143 | 0.049 | 0.837 |
| ED_-5_ | Anterior corona radiata L | 0.64 | 0.417 | 0.092 | 0.723 |
| ED_-5_ | Superior corona radiata R | 0.147 | 0.48 | 0.041 | 0.87 |
| ED_-5_ | Superior corona radiata L | -0.466 | 0.473 | -0.031 | 0.928 |
| ED_-5_ | Posterior corona radiata R | -1.753 | 0.236 | 0.082 | 0.723 |
| ED_-5_ | Posterior corona radiata L | 0.409 | 0.475 | -0.051 | 0.834 |
| ED_-5_ | Posterior thalamic radiation R | 0.356 | 0.48 | -0.015 | 0.971 |
| ED_-5_ | Posterior thalamic radiation L | 1.159 | 0.341 | -0.031 | 0.928 |
| ED_-5_ | Sagittal stratum R | -0.269 | 0.48 | 0.112 | 0.717 |
| ED_-5_ | Sagittal stratum L | 1.547 | 0.267 | -0.090 | 0.723 |
| ED_-5_ | External capsule R | -2.343 | 0.143 | 0.034 | 0.912 |
| ED_-5_ | External capsule L | -0.95 | 0.383 | -0.021 | 0.952 |
| ED_-5_ | Cingulum (cingulate gyrus) R | 0.586 | 0.424 | -0.042 | 0.87 |
| ED_-5_ | Cingulum (cingulate gyrus) L | 1.981 | 0.161 | 0.002 | 0.997 |
| ED_-5_ | Cingulum (hippocampus) R | -0.907 | 0.383 | -0.066 | 0.765 |
| ED_-5_ | Cingulum (hippocampus) L | 0.782 | 0.408 | -0.117 | 0.682 |
| ED_-5_ | Fornix (cres)/Stria terminalis R | 0.637 | 0.417 | 0.082 | 0.723 |
| ED_-5_ | Fornix (cres)/Stria terminalis L | 1.24 | 0.341 | -0.153 | 0.682 |
| ED_-5_ | Superior longitudinal fasciculus R | 0.017 | 0.493 | 0.097 | 0.723 |
| ED_-5_ | Superior longitudinal fasciculus L | 0.215 | 0.48 | -0.015 | 0.971 |
| ED_-5_ | Superior fronto-occipital fasciculus R | -0.776 | 0.408 | -0.056 | 0.825 |
| ED_-5_ | Superior fronto-occipital fasciculus L | -1.159 | 0.341 | -0.084 | 0.723 |
| ED_-5_ | Inferior fronto-occipital fasciculus R | -0.412 | 0.475 | 0.081 | 0.723 |
| ED_-5_ | Inferior fronto-occipital fasciculus L | 0.124 | 0.48 | -0.022 | 0.952 |
| ED_-5_ | Uncinate fasciculus R | 0.15 | 0.48 | 0.105 | 0.722 |
| ED_-5_ | Uncinate fasciculus L | 0.235 | 0.48 | -0.088 | 0.723 |
| ED_-5_ | Tapetum R | -0.656 | 0.417 | -0.017 | 0.971 |
| ED_-5_ | Tapetum L | 1.14 | 0.341 | -0.061 | 0.779 |
| ED_+3_ | Middle cerebellar peduncle | -0.649 | 0.983 | -0.051 | 0.834 |
| ED_+3_ | Pontine crossing tract | -0.329 | 0.983 | 0.066 | 0.767 |
| ED_+3_ | Genu of corpus callosum | -1.096 | 0.983 | 0.046 | 0.859 |
| ED_+3_ | Body of corpus callosum | -0.561 | 0.983 | 0.085 | 0.723 |
| ED_+3_ | Splenium of corpus callosum | 0.346 | 0.983 | -0.034 | 0.912 |
| ED_+3_ | Fornix (column and body of fornix) | -0.122 | 0.983 | -0.010 | 0.975 |
| ED_+3_ | Corticospinal tract R | -0.763 | 0.983 | 0.051 | 0.834 |
| ED_+3_ | Corticospinal tract L | 0.353 | 0.983 | 0.069 | 0.747 |
| ED_+3_ | Medial lemniscus R | -1.335 | 0.964 | 0.001 | 0.997 |
| ED_+3_ | Medial lemniscus L | -1.059 | 0.983 | 0.010 | 0.975 |
| ED_+3_ | Inferior cerebellar peduncle R | -0.809 | 0.983 | 0.016 | 0.971 |
| ED_+3_ | Inferior cerebellar peduncle L | 0.063 | 0.983 | 0.013 | 0.974 |
| ED_+3_ | Superior cerebellar peduncle R | -0.454 | 0.983 | 0.001 | 0.997 |
| ED_+3_ | Superior cerebellar peduncle L | -0.382 | 0.983 | 0.029 | 0.928 |
| ED_+3_ | Cerebral peduncle R | -0.167 | 0.983 | 0.025 | 0.937 |
| ED_+3_ | Cerebral peduncle L | 0.054 | 0.983 | 0.090 | 0.723 |
| ED_+3_ | Anterior limb of internal capsule R | -0.016 | 0.987 | -0.123 | 0.682 |
| ED_+3_ | Anterior limb of internal capsule L | -0.046 | 0.983 | -0.064 | 0.768 |
| ED_+3_ | Posterior limb of internal capsule R | 1.262 | 0.964 | -0.026 | 0.937 |
| ED_+3_ | Posterior limb of internal capsule L | -2.003 | 0.964 | 0.071 | 0.747 |
| ED_+3_ | Retrolenticular part of internal capsule R | 1.243 | 0.964 | 0.006 | 0.997 |
| ED_+3_ | Retrolenticular part of internal capsule L | 0.233 | 0.983 | 0.095 | 0.723 |
| ED_+3_ | Anterior corona radiata R | -0.3 | 0.983 | 0.022 | 0.952 |
| ED_+3_ | Anterior corona radiata L | -0.058 | 0.983 | -0.124 | 0.682 |
| ED_+3_ | Superior corona radiata R | 0.899 | 0.983 | -0.003 | 0.997 |
| ED_+3_ | Superior corona radiata L | -0.068 | 0.983 | 0.115 | 0.682 |
| ED_+3_ | Posterior corona radiata R | -0.601 | 0.983 | -0.158 | 0.682 |
| ED_+3_ | Posterior corona radiata L | -1.465 | 0.964 | 0.132 | 0.682 |
| ED_+3_ | Posterior thalamic radiation R | -1.209 | 0.964 | 0.106 | 0.722 |
| ED_+3_ | Posterior thalamic radiation L | 0.432 | 0.983 | -0.011 | 0.975 |
| ED_+3_ | Sagittal stratum R | 0.936 | 0.983 | -0.063 | 0.77 |
| ED_+3_ | Sagittal stratum L | 1.567 | 0.964 | 0.052 | 0.834 |
| ED_+3_ | External capsule R | 1.07 | 0.983 | -0.126 | 0.682 |
| ED_+3_ | External capsule L | -0.492 | 0.983 | -0.018 | 0.971 |
| ED_+3_ | Cingulum (cingulate gyrus) R | 0.145 | 0.983 | 0.101 | 0.722 |
| ED_+3_ | Cingulum (cingulate gyrus) L | 0.120 | 0.983 | 0.080 | 0.724 |
| ED_+3_ | Cingulum (hippocampus) R | 1.706 | 0.964 | 0.009 | 0.975 |
| ED_+3_ | Cingulum (hippocampus) L | 1.926 | 0.964 | 0.021 | 0.952 |
| ED_+3_ | Fornix (cres)/Stria terminalis R | 1.489 | 0.964 | 0.042 | 0.870 |
| ED_+3_ | Fornix (cres)/Stria terminalis L | -0.169 | 0.983 | 0.082 | 0.723 |
| ED_+3_ | Superior longitudinal fasciculus R | 0.129 | 0.983 | 0.009 | 0.975 |
| ED_+3_ | Superior longitudinal fasciculus L | -0.176 | 0.983 | 0.123 | 0.682 |
| ED_+3_ | Superior fronto-occipital fasciculus R | 1.436 | 0.964 | -0.111 | 0.719 |
| ED_+3_ | Superior fronto-occipital fasciculus L | -0.748 | 0.983 | 0.089 | 0.723 |
| ED_+3_ | Inferior fronto-occipital fasciculus R | 0.289 | 0.983 | -0.070 | 0.747 |
| ED_+3_ | Inferior fronto-occipital fasciculus L | 0.421 | 0.983 | 0.020 | 0.958 |
| ED_+3_ | Uncinate fasciculus R | 0.241 | 0.983 | -0.159 | 0.682 |
| ED_+3_ | Uncinate fasciculus L | 1.480 | 0.964 | 0.088 | 0.723 |
| ED_+3_ | Tapetum R | 0.092 | 0.983 | -0.035 | 0.912 |
| ED_+3_ | Tapetum L | 0.480 | 0.983 | -0.077 | 0.747 |
| ED_+5_ | Middle cerebellar peduncle | -1.955 | 0.091 | -0.041 | 0.870 |
| ED_+5_ | Pontine crossing tract | -2.271 | 0.046 | 0.124 | 0.682 |
| ED_+5_ | Genu of corpus callosum | -6.203 | **<0.001** | 0.158 | 0.682 |
| ED_+5_ | Body of corpus callosum | 5.407 | **<0.001** | -0.067 | 0.758 |
| ED_+5_ | Splenium of corpus callosum | -6.882 | **<0.001** | 0.151 | 0.682 |
| ED_+5_ | Fornix (column and body of fornix) | 4.836 | **<0.001** | -0.118 | 0.682 |
| ED_+5_ | Corticospinal tract R | -2.067 | 0.069 | 0.146 | 0.682 |
| ED_+5_ | Corticospinal tract L | -0.997 | 0.376 | 0.029 | 0.928 |
| ED_+5_ | Medial lemniscus R | -1.380 | 0.230 | 0.102 | 0.722 |
| ED_+5_ | Medial lemniscus L | -1.105 | 0.338 | -0.003 | 0.997 |
| ED_+5_ | Inferior cerebellar peduncle R | -1.907 | 0.095 | -0.099 | 0.723 |
| ED_+5_ | Inferior cerebellar peduncle L | -1.083 | 0.341 | -0.027 | 0.932 |
| ED_+5_ | Superior cerebellar peduncle R | -1.476 | 0.201 | 0.116 | 0.682 |
| ED_+5_ | Superior cerebellar peduncle L | -1.134 | 0.330 | 0.004 | 0.997 |
| ED_+5_ | Cerebral peduncle R | -2.602 | **0.021** | 0.144 | 0.682 |
| ED_+5_ | Cerebral peduncle L | 6.140 | **<0.001** | -0.148 | 0.682 |
| ED_+5_ | Anterior limb of internal capsule R | 3.667 | 0.001 | 0.122 | 0.682 |
| ED_+5_ | Anterior limb of internal capsule L | -4.946 | <0.001 | 0.129 | 0.682 |
| ED_+5_ | Posterior limb of internal capsule R | 5.035 | <0.001 | 0.049 | 0.837 |
| ED_+5_ | Posterior limb of internal capsule L | -1.530 | 0.183 | -0.058 | 0.806 |
| ED_+5_ | Retrolenticular part of internal capsule R | 0.770 | 0.483 | -0.094 | 0.723 |
| ED_+5_ | Retrolenticular part of internal capsule L | -1.564 | 0.181 | -0.299 | 0.031 |
| ED_+5_ | Anterior corona radiata R | -4.342 | **<0.001** | 0.134 | 0.682 |
| ED_+5_ | Anterior corona radiata L | -4.924 | **<0.001** | 0.118 | 0.682 |
| ED_+5_ | Superior corona radiata R | 6.225 | **<0.001** | 0.094 | 0.723 |
| ED_+5_ | Superior corona radiata L | 0.365 | 0.716 | -0.126 | 0.682 |
| ED_+5_ | Posterior corona radiata R | -5.639 | **<0.001** | 0.157 | 0.682 |
| ED_+5_ | Posterior corona radiata L | -5.102 | **<0.001** | -0.169 | 0.682 |
| ED_+5_ | Posterior thalamic radiation R | 2.567 | **0.022** | 0.069 | 0.747 |
| ED_+5_ | Posterior thalamic radiation L | -3.565 | **0.001** | -0.207 | 0.433 |
| ED_+5_ | Sagittal stratum R | 0.912 | 0.406 | 0.012 | 0.975 |
| ED_+5_ | Sagittal stratum L | 5.695 | **<0.001** | -0.226 | 0.310 |
| ED_+5_ | External capsule R | 4.469 | **<0.001** | 0.083 | 0.723 |
| ED_+5_ | External capsule L | -3.912 | **0.001** | -0.039 | 0.883 |
| ED_+5_ | Cingulum (cingulate gyrus) R | -4.586 | **<0.001** | 0.18 | 0.625 |
| ED_+5_ | Cingulum (cingulate gyrus) L | 5.605 | **<0.001** | -0.016 | 0.971 |
| ED_+5_ | Cingulum (hippocampus) R | 4.827 | **<0.001** | -0.05 | 0.834 |
| ED_+5_ | Cingulum (hippocampus) L | 5.397 | **<0.001** | -0.169 | 0.682 |
| ED_+5_ | Fornix (cres)/Stria terminalis R | 1.203 | 0.298 | -0.043 | 0.870 |
| ED_+5_ | Fornix (cres)/Stria terminalis L | 4.121 | **<0.001** | -0.188 | 0.581 |
| ED_+5_ | Superior longitudinal fasciculus R | -3.827 | **0.001** | 0.059 | 0.804 |
| ED_+5_ | Superior longitudinal fasciculus L | -2.772 | **0.013** | -0.186 | 0.581 |
| ED_+5_ | Superior fronto-occipital fasciculus R | 5.983 | **<0.001** | 0.102 | 0.722 |
| ED_+5_ | Superior fronto-occipital fasciculus L | 0.656 | 0.547 | 0.016 | 0.971 |
| ED_+5_ | Inferior fronto-occipital fasciculus R | -3.096 | **0.006** | 0.074 | 0.747 |
| ED_+5_ | Inferior fronto-occipital fasciculus L | 0.949 | 0.394 | 0.070 | 0.747 |
| ED_+5_ | Uncinate fasciculus R | -1.530 | 0.183 | 0.069 | 0.747 |
| ED_+5_ | Uncinate fasciculus L | -0.548 | 0.610 | -0.077 | 0.747 |
| ED_+5_ | Tapetum R | -0.437 | 0.677 | 0.069 | 0.747 |
| ED_+5_ | Tapetum L | -5.33 | **<0.001** | -0.204 | 0.433 |

P-values were adjusted for multiple comparisons using Benjamini-Hochberg’s false discovery rate (FDR) correction method.

| **2.b. Toddlers** | | | | | |
| --- | --- | --- | --- | --- | --- |
|  | tract | t | p_FDR_ | Spearman’s Rho | p_FDR_ |
| ED_-3_ | Middle cerebellar peduncle | 0.082 | 0.972 | 0.002 | 1.000 |
| ED_-3_ | Pontine crossing tract | -0.060 | 0.972 | -0.024 | 0.939 |
| ED_-3_ | Genu of corpus callosum | -1.485 | 0.864 | -0.103 | 0.659 |
| ED_-3_ | Body of corpus callosum | 0.478 | 0.972 | 0.096 | 0.680 |
| ED_-3_ | Splenium of corpus callosum | -0.894 | 0.889 | -0.059 | 0.781 |
| ED_-3_ | Fornix (column and body of fornix) | 0.648 | 0.972 | 0.119 | 0.617 |
| ED_-3_ | Corticospinal tract R | 1.306 | 0.882 | 0.143 | 0.536 |
| ED_-3_ | Corticospinal tract L | 1.127 | 0.882 | 0.104 | 0.657 |
| ED_-3_ | Medial lemniscus R | 0.965 | 0.887 | 0.106 | 0.655 |
| ED_-3_ | Medial lemniscus L | 0.184 | 0.972 | -0.008 | 0.995 |
| ED_-3_ | Inferior cerebellar peduncle R | 1.038 | 0.887 | 0.100 | 0.665 |
| ED_-3_ | Inferior cerebellar peduncle L | 0.557 | 0.972 | 0.069 | 0.761 |
| ED_-3_ | Superior cerebellar peduncle R | 1.425 | 0.864 | 0.130 | 0.600 |
| ED_-3_ | Superior cerebellar peduncle L | 1.128 | 0.882 | 0.120 | 0.617 |
| ED_-3_ | Cerebral peduncle R | 0.391 | 0.972 | 0.064 | 0.772 |
| ED_-3_ | Cerebral peduncle L | 0.199 | 0.972 | 0.053 | 0.807 |
| ED_-3_ | Anterior limb of internal capsule R | 0.446 | 0.972 | 0.098 | 0.675 |
| ED_-3_ | Anterior limb of internal capsule L | -0.336 | 0.972 | 0.002 | 1.000 |
| ED_-3_ | Posterior limb of internal capsule R | -0.230 | 0.972 | -0.010 | 0.984 |
| ED_-3_ | Posterior limb of internal capsule L | 0.124 | 0.972 | 0.079 | 0.727 |
| ED_-3_ | Retrolenticular part of internal capsule R | -1.229 | 0.882 | -0.013 | 0.977 |
| ED_-3_ | Retrolenticular part of internal capsule L | -1.456 | 0.864 | -0.083 | 0.714 |
| ED_-3_ | Anterior corona radiata R | -1.510 | 0.864 | -0.108 | 0.645 |
| ED_-3_ | Anterior corona radiata L | -1.373 | 0.864 | -0.071 | 0.756 |
| ED_-3_ | Superior corona radiata R | -0.231 | 0.972 | 0.022 | 0.946 |
| ED_-3_ | Superior corona radiata L | -0.292 | 0.972 | 0.065 | 0.772 |
| ED_-3_ | Posterior corona radiata R | -1.122 | 0.882 | -0.077 | 0.736 |
| ED_-3_ | Posterior corona radiata L | -0.903 | 0.889 | -0.059 | 0.781 |
| ED_-3_ | Posterior thalamic radiation R | -0.807 | 0.896 | -0.004 | 1.000 |
| ED_-3_ | Posterior thalamic radiation L | -0.650 | 0.972 | 0.001 | 1.000 |
| ED_-3_ | Sagittal stratum R | 0.313 | 0.972 | 0.119 | 0.617 |
| ED_-3_ | Sagittal stratum L | 0.118 | 0.972 | 0.047 | 0.835 |
| ED_-3_ | External capsule R | -0.387 | 0.972 | 0.016 | 0.965 |
| ED_-3_ | External capsule L | -1.563 | 0.864 | -0.057 | 0.792 |
| ED_-3_ | Cingulum (cingulate gyrus) R | -0.792 | 0.896 | -0.069 | 0.761 |
| ED_-3_ | Cingulum (cingulate gyrus) L | -0.986 | 0.887 | -0.062 | 0.776 |
| ED_-3_ | Cingulum (hippocampus) R | -0.177 | 0.972 | 0.008 | 0.995 |
| ED_-3_ | Cingulum (hippocampus) L | 0.212 | 0.972 | 0.073 | 0.745 |
| ED_-3_ | Fornix (cres)/Stria terminalis R | -0.858 | 0.894 | 0.023 | 0.942 |
| ED_-3_ | Fornix (cres)/Stria terminalis L | -0.556 | 0.972 | <0.001 | 1.000 |
| ED_-3_ | Superior longitudinal fasciculus R | -1.512 | 0.864 | -0.107 | 0.648 |
| ED_-3_ | Superior longitudinal fasciculus L | -1.860 | 0.864 | -0.131 | 0.598 |
| ED_-3_ | Superior fronto-occipital fasciculus R R | 1.947 | 0.864 | 0.189 | 0.416 |
| ED_-3_ | Superior fronto-occipital fasciculus R L | 0.527 | 0.972 | 0.063 | 0.772 |
| ED_-3_ | Inferior fronto-occipital fasciculus R | -0.035 | 0.972 | 0.070 | 0.758 |
| ED_-3_ | Inferior fronto-occipital fasciculus L | -0.064 | 0.972 | 0.018 | 0.957 |
| ED_-3_ | Uncinate fasciculus R | 1.056 | 0.887 | 0.147 | 0.524 |
| ED_-3_ | Uncinate fasciculus L | 0.554 | 0.972 | 0.105 | 0.655 |
| ED_-3_ | Tapetum R | -0.329 | 0.972 | -0.045 | 0.845 |
| ED_-3_ | Tapetum L | -2.108 | 0.864 | -0.182 | 0.416 |
| ED_-5_ | Middle cerebellar peduncle | -0.791 | 0.926 | -0.094 | 0.682 |
| ED_-5_ | Pontine crossing tract | 0.235 | 0.995 | -0.057 | 0.792 |
| ED_-5_ | Genu of corpus callosum | -0.900 | 0.926 | -0.111 | 0.637 |
| ED_-5_ | Body of corpus callosum | 0.619 | 0.926 | 0.067 | 0.769 |
| ED_-5_ | Splenium of corpus callosum | -1.627 | 0.840 | -0.165 | 0.470 |
| ED_-5_ | Fornix (column and body of fornix) | 0.062 | 0.995 | -0.024 | 0.939 |
| ED_-5_ | Corticospinal tract R | 1.203 | 0.926 | 0.115 | 0.623 |
| ED_-5_ | Corticospinal tract L | 0.676 | 0.926 | 0.037 | 0.882 |
| ED_-5_ | Medial lemniscus R | 1.579 | 0.840 | 0.128 | 0.603 |
| ED_-5_ | Medial lemniscus L | 0.762 | 0.926 | 0.108 | 0.642 |
| ED_-5_ | Inferior cerebellar peduncle R | 0.432 | 0.954 | 0.100 | 0.665 |
| ED_-5_ | Inferior cerebellar peduncle L | -0.090 | 0.995 | 0.004 | 1.000 |
| ED_-5_ | Superior cerebellar peduncle R | 1.744 | 0.840 | 0.157 | 0.504 |
| ED_-5_ | Superior cerebellar peduncle L | 0.693 | 0.926 | 0.133 | 0.588 |
| ED_-5_ | Cerebral peduncle R | -0.049 | 0.995 | -0.042 | 0.854 |
| ED_-5_ | Cerebral peduncle L | -0.184 | 0.995 | -0.029 | 0.918 |
| ED_-5_ | Anterior limb of internal capsule R | 0.556 | 0.935 | -0.004 | 1.000 |
| ED_-5_ | Anterior limb of internal capsule L | 0.877 | 0.926 | 0.146 | 0.524 |
| ED_-5_ | Posterior limb of internal capsule R | 0.176 | 0.995 | 0.018 | 0.957 |
| ED_-5_ | Posterior limb of internal capsule L | -0.014 | 0.995 | -0.024 | 0.937 |
| ED_-5_ | Retrolenticular part of internal capsule R | -1.037 | 0.926 | -0.075 | 0.740 |
| ED_-5_ | Retrolenticular part of internal capsule L | -1.868 | 0.840 | -0.182 | 0.416 |
| ED_-5_ | Anterior corona radiata R | -0.624 | 0.926 | -0.063 | 0.772 |
| ED_-5_ | Anterior corona radiata L | -1.007 | 0.926 | -0.058 | 0.788 |
| ED_-5_ | Superior corona radiata R | 0.156 | 0.995 | 0.043 | 0.851 |
| ED_-5_ | Superior corona radiata L | -0.818 | 0.926 | -0.066 | 0.772 |
| ED_-5_ | Posterior corona radiata R | -0.892 | 0.926 | -0.112 | 0.634 |
| ED_-5_ | Posterior corona radiata L | -0.925 | 0.926 | -0.083 | 0.714 |
| ED_-5_ | Posterior thalamic radiation R | -1.114 | 0.926 | -0.055 | 0.796 |
| ED_-5_ | Posterior thalamic radiation L | -1.758 | 0.840 | -0.179 | 0.432 |
| ED_-5_ | Sagittal stratum R | -0.215 | 0.995 | -0.002 | 1.000 |
| ED_-5_ | Sagittal stratum L | -0.431 | 0.954 | -0.010 | 0.984 |
| ED_-5_ | External capsule R | -0.229 | 0.995 | -0.002 | 1.000 |
| ED_-5_ | External capsule L | -2.758 | 0.354 | -0.240 | 0.338 |
| ED_-5_ | Cingulum (cingulate gyrus) R | -1.185 | 0.926 | -0.096 | 0.680 |
| ED_-5_ | Cingulum (cingulate gyrus) L | -0.264 | 0.995 | -0.051 | 0.808 |
| ED_-5_ | Cingulum (hippocampus) R | 0.126 | 0.995 | 0.010 | 0.984 |
| ED_-5_ | Cingulum (hippocampus) L | -0.160 | 0.995 | -0.081 | 0.725 |
| ED_-5_ | Fornix (cres)/Stria terminalis R | -1.192 | 0.926 | -0.079 | 0.727 |
| ED_-5_ | Fornix (cres)/Stria terminalis L | -0.747 | 0.926 | -0.068 | 0.767 |
| ED_-5_ | Superior longitudinal fasciculus R | -0.509 | 0.954 | -0.012 | 0.982 |
| ED_-5_ | Superior longitudinal fasciculus L | 0.778 | 0.926 | 0.096 | 0.680 |
| ED_-5_ | Superior fronto-occipital fasciculus R | 0.879 | 0.926 | 0.056 | 0.796 |
| ED_-5_ | Superior fronto-occipital fasciculus L | 0.006 | 0.995 | 0.067 | 0.771 |
| ED_-5_ | Inferior fronto-occipital fasciculus R | -0.151 | 0.995 | 0.059 | 0.784 |
| ED_-5_ | Inferior fronto-occipital fasciculus L | -0.655 | 0.926 | -0.092 | 0.686 |
| ED_-5_ | Uncinate fasciculus R | 0.782 | 0.926 | 0.121 | 0.617 |
| ED_-5_ | Uncinate fasciculus L | 0.471 | 0.954 | 0.045 | 0.842 |
| ED_-5_ | Tapetum R | -0.570 | 0.935 | -0.045 | 0.842 |
| ED_-5_ | Tapetum L | -1.953 | 0.840 | -0.185 | 0.416 |
| ED_+3_ | Middle cerebellar peduncle | 0.379 | 0.908 | 0.009 | 0.990 |
| ED_+3_ | Pontine crossing tract | 0.403 | 0.908 | 0.027 | 0.927 |
| ED_+3_ | Genu of corpus callosum | -0.868 | 0.908 | -0.071 | 0.756 |
| ED_+3_ | Body of corpus callosum | 0.758 | 0.908 | 0.122 | 0.617 |
| ED_+3_ | Splenium of corpus callosum | -0.717 | 0.908 | -0.043 | 0.851 |
| ED_+3_ | Fornix (column and body of fornix) | 0.533 | 0.908 | 0.085 | 0.707 |
| ED_+3_ | Corticospinal tract R | 1.257 | 0.908 | 0.147 | 0.524 |
| ED_+3_ | Corticospinal tract L | 1.332 | 0.908 | 0.108 | 0.642 |
| ED_+3_ | Medial lemniscus R | 1.079 | 0.908 | 0.101 | 0.660 |
| ED_+3_ | Medial lemniscus L | 0.702 | 0.908 | 0.055 | 0.796 |
| ED_+3_ | Inferior cerebellar peduncle R | 1.494 | 0.900 | 0.132 | 0.591 |
| ED_+3_ | Inferior cerebellar peduncle L | 1.492 | 0.900 | 0.148 | 0.524 |
| ED_+3_ | Superior cerebellar peduncle R | 1.745 | 0.900 | 0.181 | 0.419 |
| ED_+3_ | Superior cerebellar peduncle L | 1.769 | 0.900 | 0.182 | 0.416 |
| ED_+3_ | Cerebral peduncle R | 0.292 | 0.908 | 0.059 | 0.781 |
| ED_+3_ | Cerebral peduncle L | 0.775 | 0.908 | 0.101 | 0.660 |
| ED_+3_ | Anterior limb of internal capsule R | 1.128 | 0.908 | 0.145 | 0.524 |
| ED_+3_ | Anterior limb of internal capsule L | 0.422 | 0.908 | 0.075 | 0.738 |
| ED_+3_ | Posterior limb of internal capsule R | -0.101 | 0.958 | -0.020 | 0.955 |
| ED_+3_ | Posterior limb of internal capsule L | 0.600 | 0.908 | 0.126 | 0.603 |
| ED_+3_ | Retrolenticular part of internal capsule R | -0.292 | 0.908 | 0.039 | 0.871 |
| ED_+3_ | Retrolenticular part of internal capsule L | -0.782 | 0.908 | -0.024 | 0.937 |
| ED_+3_ | Anterior corona radiata R | -0.659 | 0.908 | -0.063 | 0.774 |
| ED_+3_ | Anterior corona radiata L | -0.430 | 0.908 | -0.028 | 0.924 |
| ED_+3_ | Superior corona radiata R | 0.108 | 0.958 | 0.055 | 0.796 |
| ED_+3_ | Superior corona radiata L | 0.009 | 0.993 | 0.073 | 0.743 |
| ED_+3_ | Posterior corona radiata R | -0.886 | 0.908 | -0.044 | 0.848 |
| ED_+3_ | Posterior corona radiata L | -0.408 | 0.908 | -0.004 | 1.000 |
| ED_+3_ | Posterior thalamic radiation R | -0.199 | 0.939 | 0.059 | 0.781 |
| ED_+3_ | Posterior thalamic radiation L | -0.172 | 0.939 | 0.021 | 0.952 |
| ED_+3_ | Sagittal stratum R | 0.972 | 0.908 | 0.159 | 0.494 |
| ED_+3_ | Sagittal stratum L | 0.488 | 0.908 | 0.065 | 0.772 |
| ED_+3_ | External capsule R | 0.279 | 0.908 | 0.088 | 0.707 |
| ED_+3_ | External capsule L | -1.202 | 0.908 | -0.080 | 0.727 |
| ED_+3_ | Cingulum (cingulate gyrus) R | -0.374 | 0.908 | -0.065 | 0.772 |
| ED_+3_ | Cingulum (cingulate gyrus) L | -0.813 | 0.908 | -0.061 | 0.780 |
| ED_+3_ | Cingulum (hippocampus) R | 0.524 | 0.908 | 0.066 | 0.772 |
| ED_+3_ | Cingulum (hippocampus) L | 0.631 | 0.908 | 0.099 | 0.667 |
| ED_+3_ | Fornix (cres)/Stria terminalis R | -0.320 | 0.908 | 0.081 | 0.723 |
| ED_+3_ | Fornix (cres)/Stria terminalis L | 0.046 | 0.983 | 0.037 | 0.879 |
| ED_+3_ | Superior longitudinal fasciculus R | -1.577 | 0.900 | -0.104 | 0.657 |
| ED_+3_ | Superior longitudinal fasciculus L | -1.240 | 0.908 | -0.083 | 0.717 |
| ED_+3_ | Superior fronto-occipital fasciculus R | 2.599 | 0.541 | 0.256 | 0.267 |
| ED_+3_ | Superior fronto-occipital fasciculus L | 0.592 | 0.908 | 0.099 | 0.667 |
| ED_+3_ | Inferior fronto-occipital fasciculus R | 0.793 | 0.908 | 0.147 | 0.524 |
| ED_+3_ | Inferior fronto-occipital fasciculus L | 0.578 | 0.908 | 0.065 | 0.772 |
| ED_+3_ | Uncinate fasciculus R | 1.473 | 0.900 | 0.192 | 0.416 |
| ED_+3_ | Uncinate fasciculus L | 0.626 | 0.908 | 0.092 | 0.686 |
| ED_+3_ | Tapetum R | -0.173 | 0.939 | -0.009 | 0.990 |
| ED_+3_ | Tapetum L | -2.064 | 0.900 | -0.124 | 0.608 |

| ED_+5_ | Middle cerebellar peduncle | -1.575 | 0.508 | -0.11 | 0.638 |
| --- | --- | --- | --- | --- | --- |
| ED_+5_ | Pontine crossing tract | -1.869 | 0.452 | -0.126 | 0.603 |
| ED_+5_ | Genu of corpus callosum | -2.241 | 0.408 | -0.191 | 0.416 |
| ED_+5_ | Body of corpus callosum | -1.863 | 0.459 | -0.065 | 0.772 |
| ED_+5_ | Splenium of corpus callosum | -1.672 | 0.497 | -0.124 | 0.611 |
| ED_+5_ | Fornix (column and body of fornix) | -1.057 | 0.669 | 0.079 | 0.727 |
| ED_+5_ | Corticospinal tract R | -1.17 | 0.637 | 0.081 | 0.723 |
| ED_+5_ | Corticospinal tract L | -1.125 | 0.642 | 0.035 | 0.887 |
| ED_+5_ | Medial lemniscus R | -1.739 | 0.474 | -0.006 | 0.999 |
| ED_+5_ | Medial lemniscus L | -2.009 | 0.429 | -0.185 | 0.416 |
| ED_+5_ | Inferior cerebellar peduncle R | -1.172 | 0.637 | 0.007 | 0.997 |
| ED_+5_ | Inferior cerebellar peduncle L | -1.477 | 0.551 | -0.045 | 0.845 |
| ED_+5_ | Superior cerebellar peduncle R | -1.335 | 0.596 | 0.07 | 0.758 |
| ED_+5_ | Superior cerebellar peduncle L | -1.798 | 0.469 | -0.087 | 0.707 |
| ED_+5_ | Cerebral peduncle R | -1.55 | 0.524 | -0.009 | 0.99 |
| ED_+5_ | Cerebral peduncle L | -1.324 | 0.596 | 0.034 | 0.894 |
| ED_+5_ | Anterior limb of internal capsule R | -2.081 | 0.419 | -0.114 | 0.626 |
| ED_+5_ | Anterior limb of internal capsule L | -1.328 | 0.596 | 0.086 | 0.707 |
| ED_+5_ | Posterior limb of internal capsule R | -1.748 | 0.472 | -0.104 | 0.657 |
| ED_+5_ | Posterior limb of internal capsule L | -1.667 | 0.497 | -0.008 | 0.994 |
| ED_+5_ | Retrolenticular part of internal capsule R | -1.178 | 0.637 | 0.063 | 0.772 |
| ED_+5_ | Retrolenticular part of internal capsule L | -1.919 | 0.429 | -0.153 | 0.512 |
| ED_+5_ | Anterior corona radiata R | -2.206 | 0.41 | -0.115 | 0.623 |
| ED_+5_ | Anterior corona radiata L | -1.784 | 0.469 | -0.077 | 0.736 |
| ED_+5_ | Superior corona radiata R | -1.724 | 0.477 | -0.017 | 0.962 |
| ED_+5_ | Superior corona radiata L | -1.943 | 0.429 | -0.097 | 0.679 |
| ED_+5_ | Posterior corona radiata R | -1.761 | 0.469 | -0.138 | 0.557 |
| ED_+5_ | Posterior corona radiata L | -2.14 | 0.419 | -0.167 | 0.466 |
| ED_+5_ | Posterior thalamic radiation R | -1.026 | 0.678 | 0.075 | 0.74 |
| ED_+5_ | Posterior thalamic radiation L | -1.526 | 0.534 | -0.055 | 0.796 |
| ED_+5_ | Sagittal stratum R | -0.786 | 0.757 | 0.07 | 0.758 |
| ED_+5_ | Sagittal stratum L | -0.161 | 0.957 | 0.077 | 0.738 |
| ED_+5_ | External capsule R | -1.723 | 0.477 | -0.002 | 0.999 |
| ED_+5_ | External capsule L | -1.779 | 0.469 | -0.124 | 0.611 |
| ED_+5_ | Cingulum (cingulate gyrus) R | -1.708 | 0.486 | -0.185 | 0.416 |
| ED_+5_ | Cingulum (cingulate gyrus) L | -1.667 | 0.497 | -0.042 | 0.854 |
| ED_+5_ | Cingulum (hippocampus) R | 0.724 | 0.786 | 0.115 | 0.623 |
| ED_+5_ | Cingulum (hippocampus) L | 0.271 | 0.941 | 0.052 | 0.808 |
| ED_+5_ | Fornix (cres)/Stria terminalis R | -0.856 | 0.737 | -0.019 | 0.957 |
| ED_+5_ | Fornix (cres)/Stria terminalis L | 0.304 | 0.93 | 0.064 | 0.772 |
| ED_+5_ | Superior longitudinal fasciculus R | -1.736 | 0.475 | -0.093 | 0.685 |
| ED_+5_ | Superior longitudinal fasciculus L | -2.06 | 0.419 | -0.195 | 0.416 |
| ED_+5_ | Superior fronto-occipital fasciculus R | -1.928 | 0.429 | -0.048 | 0.828 |
| ED_+5_ | Superior fronto-occipital fasciculus L | -1.301 | 0.609 | 0.11 | 0.638 |
| ED_+5_ | Inferior fronto-occipital fasciculus R | -1.258 | 0.629 | 0.073 | 0.743 |
| ED_+5_ | Inferior fronto-occipital fasciculus L | -0.971 | 0.704 | 0.075 | 0.738 |
| ED_+5_ | Uncinate fasciculus R | -0.606 | 0.817 | 0.061 | 0.781 |
| ED_+5_ | Uncinate fasciculus L | -0.482 | 0.859 | 0.079 | 0.727 |
| ED_+5_ | Tapetum R | -1.268 | 0.622 | -0.044 | 0.848 |
| ED_+5_ | Tapetum L | -2.05 | 0.419 | -0.17 | 0.466 |

| **2.c. Adolescents** | | | | | |
| --- | --- | --- | --- | --- | --- |
|  | tract | t | p_FDR_ | Spearman’s Rho | p_FDR_ |
| ED_-3_ | Middle cerebellar peduncle | -1.906 | 0.399 | -0.094 | 0.539 |
| ED_-3_ | Pontine crossing tract | -0.123 | 0.961 | 0.024 | 0.884 |
| ED_-3_ | Genu of corpus callosum | 0.768 | 0.658 | 0.018 | 0.927 |
| ED_-3_ | Body of corpus callosum | -1.011 | 0.568 | -0.073 | 0.638 |
| ED_-3_ | Splenium of corpus callosum | -1.911 | 0.399 | -0.131 | 0.416 |
| ED_-3_ | Fornix (column and body of fornix) | -0.229 | 0.955 | -0.049 | 0.740 |
| ED_-3_ | Corticospinal tract R | 0.029 | 0.981 | -0.005 | 0.995 |
| ED_-3_ | Corticospinal tract L | 0.418 | 0.872 | 0.064 | 0.680 |
| ED_-3_ | Medial lemniscus R | -1.189 | 0.499 | -0.070 | 0.655 |
| ED_-3_ | Medial lemniscus L | -1.501 | 0.465 | -0.084 | 0.603 |
| ED_-3_ | Inferior cerebellar peduncle R | -1.042 | 0.537 | -0.076 | 0.999 |
| ED_-3_ | Inferior cerebellar peduncle L | 0.918 | 0.608 | 0.016 | 0.937 |
| ED_-3_ | Superior cerebellar peduncle R | -1.201 | 0.499 | -0.083 | 0.607 |
| ED_-3_ | Superior cerebellar peduncle L | -1.445 | 0.465 | -0.102 | 0.511 |
| ED_-3_ | Cerebral peduncle R | -0.698 | 0.700 | -0.079 | 0.617 |
| ED_-3_ | Cerebral peduncle L | 0.475 | 0.850 | 0.012 | 0.957 |
| ED_-3_ | Anterior limb of internal capsule R | -0.587 | 0.781 | -0.061 | 0.686 |
| ED_-3_ | Anterior limb of internal capsule L | 0.261 | 0.949 | 0.012 | 0.957 |
| ED_-3_ | Posterior limb of internal capsule R | -0.023 | 0.981 | -0.007 | 0.984 |
| ED_-3_ | Posterior limb of internal capsule L | 0.361 | 0.903 | 0.010 | 0.965 |
| ED_-3_ | Retrolenticular part of internal capsule R | -0.776 | 0.658 | -0.039 | 0.784 |
| ED_-3_ | Retrolenticular part of internal capsule L | -0.864 | 0.635 | -0.090 | 0.572 |
| ED_-3_ | Anterior corona radiata R | -1.143 | 0.499 | -0.063 | 0.680 |
| ED_-3_ | Anterior corona radiata L | -0.771 | 0.658 | -0.049 | 0.743 |
| ED_-3_ | Superior corona radiata R | -2.009 | 0.399 | -0.126 | 0.416 |
| ED_-3_ | Superior corona radiata L | -1.639 | 0.465 | -0.122 | 0.416 |
| ED_-3_ | Posterior corona radiata R | -2.170 | 0.380 | -0.126 | 0.416 |
| ED_-3_ | Posterior corona radiata L | -2.436 | 0.315 | -0.166 | 0.278 |
| ED_-3_ | Posterior thalamic radiation R | -1.800 | 0.399 | -0.099 | 0.524 |
| ED_-3_ | Posterior thalamic radiation L | -1.123 | 0.499 | -0.077 | 0.623 |
| ED_-3_ | Sagittal stratum R | -1.118 | 0.499 | -0.101 | 0.513 |
| ED_-3_ | Sagittal stratum L | -1.438 | 0.465 | -0.143 | 0.397 |
| ED_-3_ | External capsule R | -1.197 | 0.499 | -0.071 | 0.648 |
| ED_-3_ | External capsule L | -1.331 | 0.499 | -0.099 | 0.524 |
| ED_-3_ | Cingulum (cingulate gyrus) R | -1.600 | 0.465 | -0.116 | 0.449 |
| ED_-3_ | Cingulum (cingulate gyrus) L | -1.193 | 0.499 | -0.101 | 0.513 |
| ED_-3_ | Cingulum (hippocampus) R | -1.457 | 0.465 | -0.070 | 0.655 |
| ED_-3_ | Cingulum (hippocampus) L | 0.466 | 0.850 | 0.031 | 0.833 |
| ED_-3_ | Fornix (cres)/Stria terminalis R | -1.555 | 0.465 | -0.105 | 0.497 |
| ED_-3_ | Fornix (cres)/Stria terminalis L | -0.931 | 0.608 | -0.063 | 0.681 |
| ED_-3_ | Superior longitudinal fasciculus R | -1.191 | 0.499 | -0.115 | 0.457 |
| ED_-3_ | Superior longitudinal fasciculus L | -0.279 | 0.949 | -0.054 | 0.723 |
| ED_-3_ | Superior fronto-occipital fasciculus R | -2.357 | 0.315 | -0.127 | 0.416 |
| ED_-3_ | Superior fronto-occipital fasciculus L | -1.137 | 0.499 | -0.098 | 0.524 |
| ED_-3_ | Inferior fronto-occipital fasciculus R | 0.038 | 0.981 | -0.005 | 0.997 |
| ED_-3_ | Inferior fronto-occipital fasciculus L | -0.205 | 0.955 | -0.056 | 0.708 |
| ED_-3_ | Uncinate fasciculus R | 0.154 | 0.956 | -0.025 | 0.879 |
| ED_-3_ | Uncinate fasciculus L | -0.159 | 0.956 | -0.060 | 0.691 |
| ED_-3_ | Tapetum R | -1.812 | 0.399 | -0.097 | 0.524 |
| ED_-3_ | Tapetum L | -2.754 | 0.312 | -0.180 | 0.267 |
| ED_-5_ | Middle cerebellar peduncle | -1.526 | 0.420 | -0.100 | 0.519 |
| ED_-5_ | Pontine crossing tract | 0.150 | 0.981 | 0.055 | 0.714 |
| ED_-5_ | Genu of corpus callosum | 0.105 | 0.987 | -0.033 | 0.811 |
| ED_-5_ | Body of corpus callosum | -1.974 | 0.247 | -0.130 | 0.416 |
| ED_-5_ | Splenium of corpus callosum | -2.104 | 0.247 | -0.146 | 0.362 |
| ED_-5_ | Fornix (column and body of fornix) | -0.788 | 0.650 | -0.076 | 0.623 |
| ED_-5_ | Corticospinal tract R | 0.406 | 0.908 | 0.020 | 0.914 |
| ED_-5_ | Corticospinal tract L | -0.540 | 0.836 | -0.016 | 0.939 |
| ED_-5_ | Medial lemniscus R | -0.183 | 0.977 | -0.008 | 0.977 |
| ED_-5_ | Medial lemniscus L | -1.967 | 0.247 | -0.111 | 0.466 |
| ED_-5_ | Inferior cerebellar peduncle R | -2.179 | 0.578 | -0.145 | 0.794 |
| ED_-5_ | Inferior cerebellar peduncle L | -0.425 | 0.908 | -0.069 | 0.657 |
| ED_-5_ | Superior cerebellar peduncle R | -1.196 | 0.524 | -0.103 | 0.504 |
| ED_-5_ | Superior cerebellar peduncle L | -2.229 | 0.247 | -0.141 | 0.404 |
| ED_-5_ | Cerebral peduncle R | -0.024 | 0.987 | -0.027 | 0.863 |
| ED_-5_ | Cerebral peduncle L | -0.802 | 0.650 | -0.054 | 0.723 |
| ED_-5_ | Anterior limb of internal capsule R | -0.343 | 0.944 | -0.026 | 0.871 |
| ED_-5_ | Anterior limb of internal capsule L | -0.205 | 0.977 | -0.019 | 0.917 |
| ED_-5_ | Posterior limb of internal capsule R | 0.053 | 0.987 | 0.002 | 0.999 |
| ED_-5_ | Posterior limb of internal capsule L | -0.180 | 0.977 | 0.001 | 0.999 |
| ED_-5_ | Retrolenticular part of internal capsule R | -1.046 | 0.538 | -0.051 | 0.736 |
| ED_-5_ | Retrolenticular part of internal capsule L | -1.749 | 0.286 | -0.112 | 0.466 |
| ED_-5_ | Anterior corona radiata R | -1.075 | 0.534 | -0.085 | 0.603 |
| ED_-5_ | Anterior corona radiata L | -0.778 | 0.650 | -0.062 | 0.682 |
| ED_-5_ | Superior corona radiata R | -1.845 | 0.271 | -0.146 | 0.362 |
| ED_-5_ | Superior corona radiata L | -1.242 | 0.524 | -0.107 | 0.490 |
| ED_-5_ | Posterior corona radiata R | -2.833 | 0.108 | -0.176 | 0.267 |
| ED_-5_ | Posterior corona radiata L | -3.346 | **0.047** | -0.248 | 0.063 |
| ED_-5_ | Posterior thalamic radiation R | -1.138 | 0.524 | -0.047 | 0.753 |
| ED_-5_ | Posterior thalamic radiation L | -1.809 | 0.271 | -0.117 | 0.445 |
| ED_-5_ | Sagittal stratum R | -0.847 | 0.650 | -0.061 | 0.688 |
| ED_-5_ | Sagittal stratum L | -2.105 | 0.247 | -0.140 | 0.404 |
| ED_-5_ | External capsule R | -1.188 | 0.524 | -0.078 | 0.623 |
| ED_-5_ | External capsule L | -2.050 | 0.247 | -0.137 | 0.404 |
| ED_-5_ | Cingulum (cingulate gyrus) R | -2.291 | 0.247 | -0.186 | 0.267 |
| ED_-5_ | Cingulum (cingulate gyrus) L | -1.297 | 0.524 | -0.108 | 0.476 |
| ED_-5_ | Cingulum (hippocampus) R | -0.931 | 0.596 | -0.041 | 0.776 |
| ED_-5_ | Cingulum (hippocampus) L | -1.469 | 0.439 | -0.111 | 0.466 |
| ED_-5_ | Fornix (cres)/Stria terminalis R | -1.916 | 0.253 | -0.085 | 0.603 |
| ED_-5_ | Fornix (cres)/Stria terminalis L | -1.156 | 0.524 | -0.086 | 0.602 |
| ED_-5_ | Superior longitudinal fasciculus R | -0.224 | 0.977 | -0.047 | 0.756 |
| ED_-5_ | Superior longitudinal fasciculus L | -0.529 | 0.836 | -0.034 | 0.808 |
| ED_-5_ | Superior fronto-occipital fasciculus R | -1.107 | 0.529 | -0.081 | 0.617 |
| ED_-5_ | Superior fronto-occipital fasciculus L | -0.273 | 0.977 | -0.111 | 0.466 |
| ED_-5_ | Inferior fronto-occipital fasciculus R | -0.021 | 0.987 | -0.001 | 0.999 |
| ED_-5_ | Inferior fronto-occipital fasciculus L | -1.020 | 0.541 | -0.069 | 0.657 |
| ED_-5_ | Uncinate fasciculus R | 0.017 | 0.987 | 0.004 | 0.999 |
| ED_-5_ | Uncinate fasciculus L | -1.327 | 0.524 | -0.116 | 0.449 |
| ED_-5_ | Tapetum R | -1.149 | 0.524 | -0.074 | 0.634 |
| ED_-5_ | Tapetum L | -2.742 | 0.108 | -0.171 | 0.267 |
| ED_+3_ | Middle cerebellar peduncle | -1.330 | 0.266 | -0.067 | 0.660 |
| ED_+3_ | Pontine crossing tract | -0.402 | 0.749 | -0.008 | 0.977 |
| ED_+3_ | Genu of corpus callosum | -0.337 | 0.785 | -0.051 | 0.736 |
| ED_+3_ | Body of corpus callosum | -2.192 | 0.087 | -0.147 | 0.362 |
| ED_+3_ | Splenium of corpus callosum | -2.722 | **0.043** | -0.172 | 0.267 |
| ED_+3_ | Fornix (column and body of fornix) | -1.112 | 0.338 | -0.104 | 0.503 |
| ED_+3_ | Corticospinal tract R | -0.696 | 0.582 | -0.036 | 0.796 |
| ED_+3_ | Corticospinal tract L | 0.028 | 0.978 | 0.026 | 0.871 |
| ED_+3_ | Medial lemniscus R | -1.160 | 0.327 | -0.089 | 0.588 |
| ED_+3_ | Medial lemniscus L | -1.631 | 0.175 | -0.107 | 0.490 |
| ED_+3_ | Inferior cerebellar peduncle R | -1.508 | 0.508 | -0.116 | 0.951 |
| ED_+3_ | Inferior cerebellar peduncle L | 0.246 | 0.841 | -0.019 | 0.923 |
| ED_+3_ | Superior cerebellar peduncle R | -1.618 | 0.175 | -0.109 | 0.470 |
| ED_+3_ | Superior cerebellar peduncle L | -1.853 | 0.128 | -0.146 | 0.362 |
| ED_+3_ | Cerebral peduncle R | -1.774 | 0.146 | -0.131 | 0.416 |
| ED_+3_ | Cerebral peduncle L | -0.035 | 0.978 | -0.002 | 0.999 |
| ED_+3_ | Anterior limb of internal capsule R | -1.418 | 0.241 | -0.093 | 0.544 |
| ED_+3_ | Anterior limb of internal capsule L | -1.571 | 0.186 | -0.106 | 0.492 |
| ED_+3_ | Posterior limb of internal capsule R | -0.783 | 0.532 | -0.027 | 0.863 |
| ED_+3_ | Posterior limb of internal capsule L | -0.422 | 0.749 | -0.018 | 0.927 |
| ED_+3_ | Retrolenticular part of internal capsule R | -2.248 | 0.083 | -0.122 | 0.416 |
| ED_+3_ | Retrolenticular part of internal capsule L | -1.369 | 0.256 | -0.106 | 0.492 |
| ED_+3_ | Anterior corona radiata R | -2.117 | 0.091 | -0.132 | 0.416 |
| ED_+3_ | Anterior corona radiata L | -2.179 | 0.087 | -0.150 | 0.362 |
| ED_+3_ | Superior corona radiata R | -2.766 | **0.043** | -0.154 | 0.356 |
| ED_+3_ | Superior corona radiata L | -2.793 | **0.043** | -0.171 | 0.267 |
| ED_+3_ | Posterior corona radiata R | -2.704 | **0.043** | -0.152 | 0.361 |
| ED_+3_ | Posterior corona radiata L | -3.146 | **0.043** | -0.210 | 0.214 |
| ED_+3_ | Posterior thalamic radiation R | -2.733 | **0.043** | -0.155 | 0.356 |
| ED_+3_ | Posterior thalamic radiation L | -2.352 | 0.068 | -0.137 | 0.404 |
| ED_+3_ | Sagittal stratum R | -2.552 | **0.043** | -0.189 | 0.267 |
| ED_+3_ | Sagittal stratum L | -2.553 | **0.043** | -0.216 | 0.199 |
| ED_+3_ | External capsule R | -2.572 | **0.043** | -0.155 | 0.356 |
| ED_+3_ | External capsule L | -2.160 | 0.087 | -0.130 | 0.416 |
| ED_+3_ | Cingulum (cingulate gyrus) R | -2.694 | **0.043** | -0.179 | 0.267 |
| ED_+3_ | Cingulum (cingulate gyrus) L | -2.614 | **0.043** | -0.170 | 0.267 |
| ED_+3_ | Cingulum (hippocampus) R | -2.058 | 0.092 | -0.114 | 0.457 |
| ED_+3_ | Cingulum (hippocampus) L | -0.472 | 0.727 | -0.043 | 0.772 |
| ED_+3_ | Fornix (cres)/Stria terminalis R | -1.637 | 0.175 | -0.135 | 0.416 |
| ED_+3_ | Fornix (cres)/Stria terminalis L | -1.728 | 0.155 | -0.115 | 0.449 |
| ED_+3_ | Superior longitudinal fasciculus R | -2.017 | 0.092 | -0.156 | 0.356 |
| ED_+3_ | Superior longitudinal fasciculus L | -2.083 | 0.092 | -0.129 | 0.416 |
| ED_+3_ | Superior fronto-occipital fasciculus R | -2.625 | **0.043** | -0.148 | 0.362 |
| ED_+3_ | Superior fronto-occipital fasciculus L | -2.039 | 0.092 | -0.133 | 0.416 |
| ED_+3_ | Inferior fronto-occipital fasciculus R | -1.226 | 0.301 | -0.093 | 0.548 |
| ED_+3_ | Inferior fronto-occipital fasciculus L | -1.310 | 0.268 | -0.125 | 0.416 |
| ED_+3_ | Uncinate fasciculus R | -0.545 | 0.684 | -0.071 | 0.650 |
| ED_+3_ | Uncinate fasciculus L | -1.108 | 0.338 | -0.140 | 0.404 |
| ED_+3_ | Tapetum R | -2.017 | 0.092 | -0.103 | 0.504 |
| ED_+3_ | Tapetum L | -2.639 | **0.043** | -0.180 | 0.267 |
| ED_+5_ | Middle cerebellar peduncle | -0.912 | 0.693 | -0.084 | 0.603 |
| ED_+5_ | Pontine crossing tract | 0.901 | 0.693 | 0.033 | 0.820 |
| ED_+5_ | Genu of corpus callosum | 1.059 | 0.693 | 0.036 | 0.796 |
| ED_+5_ | Body of corpus callosum | -0.876 | 0.693 | -0.172 | 0.267 |
| ED_+5_ | Splenium of corpus callosum | -1.051 | 0.693 | -0.126 | 0.416 |
| ED_+5_ | Fornix (column and body of fornix) | -1.211 | 0.693 | -0.098 | 0.524 |
| ED_+5_ | Corticospinal tract R | -0.190 | 0.939 | -0.019 | 0.917 |
| ED_+5_ | Corticospinal tract L | -0.510 | 0.880 | -0.057 | 0.707 |
| ED_+5_ | Medial lemniscus R | 1.245 | 0.693 | 0.036 | 0.796 |
| ED_+5_ | Medial lemniscus L | 0.149 | 0.939 | -0.120 | 0.423 |
| ED_+5_ | Inferior cerebellar peduncle R | 0.309 | 0.939 | -0.138 | 0.821 |
| ED_+5_ | Inferior cerebellar peduncle L | 0.320 | 0.939 | -0.036 | 0.796 |
| ED_+5_ | Superior cerebellar peduncle R | -0.255 | 0.939 | -0.068 | 0.659 |
| ED_+5_ | Superior cerebellar peduncle L | 0.172 | 0.939 | -0.130 | 0.416 |
| ED_+5_ | Cerebral peduncle R | -0.645 | 0.828 | -0.041 | 0.780 |
| ED_+5_ | Cerebral peduncle L | -1.343 | 0.693 | -0.121 | 0.416 |
| ED_+5_ | Anterior limb of internal capsule R | 1.099 | 0.693 | 0.021 | 0.909 |
| ED_+5_ | Anterior limb of internal capsule L | 0.220 | 0.939 | -0.051 | 0.736 |
| ED_+5_ | Posterior limb of internal capsule R | -0.380 | 0.939 | -0.018 | 0.924 |
| ED_+5_ | Posterior limb of internal capsule L | -0.444 | 0.921 | -0.068 | 0.660 |
| ED_+5_ | Retrolenticular part of internal capsule R | 0.161 | 0.939 | -0.043 | 0.772 |
| ED_+5_ | Retrolenticular part of internal capsule L | -1.334 | 0.693 | -0.092 | 0.549 |
| ED_+5_ | Anterior corona radiata R | 0.969 | 0.693 | 0.013 | 0.956 |
| ED_+5_ | Anterior corona radiata L | 0.291 | 0.939 | -0.036 | 0.796 |
| ED_+5_ | Superior corona radiata R | -0.939 | 0.693 | -0.127 | 0.416 |
| ED_+5_ | Superior corona radiata L | -1.429 | 0.693 | -0.179 | 0.267 |
| ED_+5_ | Posterior corona radiata R | -0.807 | 0.711 | -0.090 | 0.574 |
| ED_+5_ | Posterior corona radiata L | -1.600 | 0.693 | -0.185 | 0.267 |
| ED_+5_ | Posterior thalamic radiation R | -0.895 | 0.693 | -0.137 | 0.404 |
| ED_+5_ | Posterior thalamic radiation L | -2.065 | 0.686 | -0.118 | 0.438 |
| ED_+5_ | Sagittal stratum R | -0.930 | 0.693 | -0.115 | 0.449 |
| ED_+5_ | Sagittal stratum L | -2.605 | 0.479 | -0.183 | 0.267 |
| ED_+5_ | External capsule R | 0.038 | 0.994 | 0.015 | 0.942 |
| ED_+5_ | External capsule L | -0.544 | 0.872 | -0.065 | 0.675 |
| ED_+5_ | Cingulum (cingulate gyrus) R | -0.160 | 0.939 | -0.053 | 0.727 |
| ED_+5_ | Cingulum (cingulate gyrus) L | -2.045 | 0.686 | -0.133 | 0.416 |
| ED_+5_ | Cingulum (hippocampus) R | -1.412 | 0.693 | -0.111 | 0.466 |
| ED_+5_ | Cingulum (hippocampus) L | -1.275 | 0.693 | -0.089 | 0.584 |
| ED_+5_ | Fornix (cres)/Stria terminalis R | -0.833 | 0.710 | -0.079 | 0.617 |
| ED_+5_ | Fornix (cres)/Stria terminalis L | -1.711 | 0.693 | -0.128 | 0.416 |
| ED_+5_ | Superior longitudinal fasciculus R | -0.022 | 0.994 | -0.096 | 0.525 |
| ED_+5_ | Superior longitudinal fasciculus L | -1.234 | 0.693 | -0.062 | 0.682 |
| ED_+5_ | Superior fronto-occipital fasciculus R | -0.302 | 0.939 | -0.111 | 0.466 |
| ED_+5_ | Superior fronto-occipital fasciculus L | -0.615 | 0.828 | -0.166 | 0.278 |
| ED_+5_ | Inferior fronto-occipital fasciculus R | 1.283 | 0.693 | 0.057 | 0.707 |
| ED_+5_ | Inferior fronto-occipital fasciculus L | -0.612 | 0.828 | -0.037 | 0.796 |
| ED_+5_ | Uncinate fasciculus R | 1.120 | 0.693 | 0.081 | 0.617 |
| ED_+5_ | Uncinate fasciculus L | -0.977 | 0.693 | -0.098 | 0.524 |
| ED_+5_ | Tapetum R | 0.008 | 0.994 | 0.012 | 0.957 |
| ED_+5_ | Tapetum L | -1.456 | 0.693 | -0.105 | 0.495 |

P-values were adjusted for multiple comparisons using Benjamini-Hochberg’s false discovery rate (FDR) correction method.

| **3.d. Adults** | | | | | |
| --- | --- | --- | --- | --- | --- |
|  | tract | t | p_FDR_ | Spearman’s Rho | p_FDR_ |
| ED_-3_ | Middle cerebellar peduncle | -1.533 | 0.425 | -0.179 | 0.466 |
| ED_-3_ | Pontine crossing tract | -1.391 | 0.451 | -0.166 | 0.504 |
| ED_-3_ | Genu of corpus callosum | -0.644 | 0.747 | -0.060 | 0.796 |
| ED_-3_ | Body of corpus callosum | -0.657 | 0.747 | -0.065 | 0.781 |
| ED_-3_ | Splenium of corpus callosum | -1.702 | 0.364 | -0.146 | 0.569 |
| ED_-3_ | Fornix (column and body of fornix) | -1.933 | 0.330 | -0.210 | 0.416 |
| ED_-3_ | Corticospinal tract R | -1.849 | 0.361 | -0.221 | 0.404 |
| ED_-3_ | Corticospinal tract L | -0.503 | 0.773 | -0.068 | 0.772 |
| ED_-3_ | Medial lemniscus R | -1.828 | 0.361 | -0.176 | 0.470 |
| ED_-3_ | Medial lemniscus L | -1.177 | 0.551 | -0.118 | 0.638 |
| ED_-3_ | Inferior cerebellar peduncle R | -0.896 | 0.701 | -0.064 | 0.781 |
| ED_-3_ | Inferior cerebellar peduncle L | -0.013 | 0.992 | 0.046 | 0.851 |
| ED_-3_ | Superior cerebellar peduncle R | -2.013 | 0.330 | -0.222 | 0.404 |
| ED_-3_ | Superior cerebellar peduncle L | -0.661 | 0.747 | -0.124 | 0.623 |
| ED_-3_ | Cerebral peduncle R | -3.644 | **0.039** | -0.350 | 0.199 |
| ED_-3_ | Cerebral peduncle L | 0.760 | 0.747 | 0.057 | 0.806 |
| ED_-3_ | Anterior limb of internal capsule R | -1.642 | 0.364 | -0.138 | 0.603 |
| ED_-3_ | Anterior limb of internal capsule L | 0.828 | 0.713 | 0.116 | 0.645 |
| ED_-3_ | Posterior limb of internal capsule R | -2.525 | 0.215 | -0.219 | 0.407 |
| ED_-3_ | Posterior limb of internal capsule L | -0.560 | 0.761 | -0.093 | 0.707 |
| ED_-3_ | Retrolenticular part of internal capsule R | -1.697 | 0.364 | -0.185 | 0.457 |
| ED_-3_ | Retrolenticular part of internal capsule L | -0.999 | 0.647 | -0.118 | 0.638 |
| ED_-3_ | Anterior corona radiata R | 0.599 | 0.747 | 0.002 | 0.999 |
| ED_-3_ | Anterior corona radiata L | 2.397 | 0.215 | 0.252 | 0.356 |
| ED_-3_ | Superior corona radiata R | 0.025 | 0.992 | 0.026 | 0.937 |
| ED_-3_ | Superior corona radiata L | 0.607 | 0.747 | 0.074 | 0.761 |
| ED_-3_ | Posterior corona radiata R | -1.099 | 0.582 | -0.130 | 0.617 |
| ED_-3_ | Posterior corona radiata L | -0.362 | 0.813 | -0.032 | 0.914 |
| ED_-3_ | Posterior thalamic radiation R | -1.362 | 0.451 | -0.155 | 0.524 |
| ED_-3_ | Posterior thalamic radiation L | -0.346 | 0.813 | -0.023 | 0.948 |
| ED_-3_ | Sagittal stratum R | -1.459 | 0.451 | -0.170 | 0.494 |
| ED_-3_ | Sagittal stratum L | -1.675 | 0.364 | -0.196 | 0.416 |
| ED_-3_ | External capsule R | -0.725 | 0.747 | -0.097 | 0.691 |
| ED_-3_ | External capsule L | -0.270 | 0.857 | -0.029 | 0.926 |
| ED_-3_ | Cingulum (cingulate gyrus) R | -3.037 | 0.116 | -0.303 | 0.267 |
| ED_-3_ | Cingulum (cingulate gyrus) L | -0.391 | 0.812 | -0.038 | 0.888 |
| ED_-3_ | Cingulum (hippocampus) R | -0.534 | 0.764 | -0.079 | 0.743 |
| ED_-3_ | Cingulum (hippocampus) L | 0.434 | 0.793 | 0.050 | 0.835 |
| ED_-3_ | Fornix (cres)/Stria terminalis R | -0.010 | 0.992 | -0.017 | 0.964 |
| ED_-3_ | Fornix (cres)/Stria terminalis L | -0.471 | 0.781 | -0.055 | 0.808 |
| ED_-3_ | Superior longitudinal fasciculus R | -0.840 | 0.713 | -0.071 | 0.772 |
| ED_-3_ | Superior longitudinal fasciculus L | 0.924 | 0.695 | 0.115 | 0.648 |
| ED_-3_ | Superior fronto-occipital fasciculus R | 2.149 | 0.296 | 0.137 | 0.603 |
| ED_-3_ | Superior fronto-occipital fasciculus L | 2.456 | 0.215 | 0.208 | 0.416 |
| ED_-3_ | Inferior fronto-occipital fasciculus R | -1.386 | 0.451 | -0.179 | 0.466 |
| ED_-3_ | Inferior fronto-occipital fasciculus L | -0.125 | 0.959 | -0.019 | 0.957 |
| ED_-3_ | Uncinate fasciculus R | -1.970 | 0.330 | -0.205 | 0.416 |
| ED_-3_ | Uncinate fasciculus L | -1.160 | 0.551 | -0.125 | 0.623 |
| ED_-3_ | Tapetum R | -1.162 | 0.551 | -0.149 | 0.549 |
| ED_-3_ | Tapetum L | -0.600 | 0.747 | -0.003 | 0.999 |
| ED_-5_ | Middle cerebellar peduncle | -0.938 | 0.730 | -0.025 | 0.939 |
| ED_-5_ | Pontine crossing tract | -1.602 | 0.671 | -0.129 | 0.617 |
| ED_-5_ | Genu of corpus callosum | -0.700 | 0.734 | -0.073 | 0.767 |
| ED_-5_ | Body of corpus callosum | -1.985 | 0.449 | -0.197 | 0.416 |
| ED_-5_ | Splenium of corpus callosum | -0.101 | 0.920 | -0.019 | 0.957 |
| ED_-5_ | Fornix (column and body of fornix) | -1.215 | 0.730 | -0.140 | 0.599 |
| ED_-5_ | Corticospinal tract R | -1.071 | 0.730 | -0.113 | 0.655 |
| ED_-5_ | Corticospinal tract L | -1.198 | 0.730 | -0.188 | 0.449 |
| ED_-5_ | Medial lemniscus R | -0.705 | 0.734 | 0.001 | 0.999 |
| ED_-5_ | Medial lemniscus L | 0.258 | 0.871 | 0.083 | 0.736 |
| ED_-5_ | Inferior cerebellar peduncle R | -0.375 | 0.829 | -0.121 | 0.634 |
| ED_-5_ | Inferior cerebellar peduncle L | 0.918 | 0.730 | 0.162 | 0.514 |
| ED_-5_ | Superior cerebellar peduncle R | -1.821 | 0.511 | -0.131 | 0.617 |
| ED_-5_ | Superior cerebellar peduncle L | -0.846 | 0.730 | -0.056 | 0.808 |
| ED_-5_ | Cerebral peduncle R | -1.008 | 0.730 | -0.119 | 0.638 |
| ED_-5_ | Cerebral peduncle L | 1.352 | 0.730 | 0.100 | 0.682 |
| ED_-5_ | Anterior limb of internal capsule R | -0.786 | 0.730 | -0.125 | 0.623 |
| ED_-5_ | Anterior limb of internal capsule L | -0.852 | 0.730 | -0.017 | 0.964 |
| ED_-5_ | Posterior limb of internal capsule R | -0.857 | 0.730 | -0.123 | 0.623 |
| ED_-5_ | Posterior limb of internal capsule L | -0.191 | 0.886 | -0.002 | 0.999 |
| ED_-5_ | Retrolenticular part of internal capsule R | -1.201 | 0.730 | -0.143 | 0.588 |
| ED_-5_ | Retrolenticular part of internal capsule L | 0.495 | 0.800 | 0.081 | 0.738 |
| ED_-5_ | Anterior corona radiata R | -0.610 | 0.747 | -0.123 | 0.623 |
| ED_-5_ | Anterior corona radiata L | 1.092 | 0.730 | 0.095 | 0.705 |
| ED_-5_ | Superior corona radiata R | -0.417 | 0.828 | -0.091 | 0.707 |
| ED_-5_ | Superior corona radiata L | 0.463 | 0.807 | 0.001 | 0.999 |
| ED_-5_ | Posterior corona radiata R | 1.515 | 0.671 | 0.014 | 0.977 |
| ED_-5_ | Posterior corona radiata L | -1.838 | 0.511 | -0.272 | 0.267 |
| ED_-5_ | Posterior thalamic radiation R | 0.682 | 0.734 | 0.045 | 0.856 |
| ED_-5_ | Posterior thalamic radiation L | 1.438 | 0.708 | 0.091 | 0.707 |
| ED_-5_ | Sagittal stratum R | -0.237 | 0.871 | -0.054 | 0.815 |
| ED_-5_ | Sagittal stratum L | 2.133 | 0.449 | 0.241 | 0.362 |
| ED_-5_ | External capsule R | -1.024 | 0.730 | -0.128 | 0.617 |
| ED_-5_ | External capsule L | 1.034 | 0.730 | 0.100 | 0.682 |
| ED_-5_ | Cingulum (cingulate gyrus) R | -2.628 | 0.330 | -0.291 | 0.267 |
| ED_-5_ | Cingulum (cingulate gyrus) L | -0.943 | 0.730 | -0.151 | 0.539 |
| ED_-5_ | Cingulum (hippocampus) R | -0.342 | 0.834 | -0.162 | 0.514 |
| ED_-5_ | Cingulum (hippocampus) L | 3.744 | **0.016** | 0.160 | 0.524 |
| ED_-5_ | Fornix (cres)/Stria terminalis R | -0.166 | 0.887 | -0.108 | 0.660 |
| ED_-5_ | Fornix (cres)/Stria terminalis L | 1.998 | 0.449 | 0.128 | 0.617 |
| ED_-5_ | Superior longitudinal fasciculus R | 2.202 | 0.449 | 0.151 | 0.539 |
| ED_-5_ | Superior longitudinal fasciculus L | -0.795 | 0.730 | -0.070 | 0.772 |
| ED_-5_ | Superior fronto-occipital fasciculus R | -0.370 | 0.829 | -0.081 | 0.738 |
| ED_-5_ | Superior fronto-occipital fasciculus L | 1.033 | 0.730 | 0.032 | 0.914 |
| ED_-5_ | Inferior fronto-occipital fasciculus R | 0.701 | 0.734 | 0.079 | 0.743 |
| ED_-5_ | Inferior fronto-occipital fasciculus L | 0.526 | 0.793 | 0.151 | 0.539 |
| ED_-5_ | Uncinate fasciculus R | 0.824 | 0.730 | 0.102 | 0.680 |
| ED_-5_ | Uncinate fasciculus L | 0.617 | 0.747 | 0.130 | 0.617 |
| ED_-5_ | Tapetum R | 0.598 | 0.747 | -0.050 | 0.835 |
| ED_-5_ | Tapetum L | -0.231 | 0.871 | -0.152 | 0.538 |
| ED_+3_ | Middle cerebellar peduncle | -2.496 | 0.173 | -0.294 | 0.267 |
| ED_+3_ | Pontine crossing tract | -2.265 | 0.173 | -0.260 | 0.321 |
| ED_+3_ | Genu of corpus callosum | -2.282 | 0.173 | -0.196 | 0.416 |
| ED_+3_ | Body of corpus callosum | -1.757 | 0.271 | -0.155 | 0.524 |
| ED_+3_ | Splenium of corpus callosum | -2.271 | 0.173 | -0.197 | 0.416 |
| ED_+3_ | Fornix (column and body of fornix) | -0.620 | 0.832 | -0.095 | 0.705 |
| ED_+3_ | Corticospinal tract R | -2.143 | 0.181 | -0.255 | 0.339 |
| ED_+3_ | Corticospinal tract L | -1.394 | 0.428 | -0.184 | 0.460 |
| ED_+3_ | Medial lemniscus R | -2.327 | 0.173 | -0.246 | 0.356 |
| ED_+3_ | Medial lemniscus L | -1.987 | 0.192 | -0.218 | 0.412 |
| ED_+3_ | Inferior cerebellar peduncle R | -1.183 | 0.539 | -0.081 | 0.738 |
| ED_+3_ | Inferior cerebellar peduncle L | -0.487 | 0.832 | -0.044 | 0.863 |
| ED_+3_ | Superior cerebellar peduncle R | -2.090 | 0.188 | -0.211 | 0.416 |
| ED_+3_ | Superior cerebellar peduncle L | -1.965 | 0.192 | -0.236 | 0.362 |
| ED_+3_ | Cerebral peduncle R | -2.567 | 0.173 | -0.312 | 0.267 |
| ED_+3_ | Cerebral peduncle L | -1.427 | 0.428 | -0.124 | 0.623 |
| ED_+3_ | Anterior limb of internal capsule R | -2.236 | 0.173 | -0.237 | 0.362 |
| ED_+3_ | Anterior limb of internal capsule L | -0.722 | 0.766 | -0.080 | 0.740 |
| ED_+3_ | Posterior limb of internal capsule R | -0.482 | 0.832 | -0.064 | 0.781 |
| ED_+3_ | Posterior limb of internal capsule L | -0.081 | 0.967 | -0.015 | 0.974 |
| ED_+3_ | Retrolenticular part of internal capsule R | -0.886 | 0.659 | -0.056 | 0.808 |
| ED_+3_ | Retrolenticular part of internal capsule L | -0.096 | 0.967 | 0.020 | 0.957 |
| ED_+3_ | Anterior corona radiata R | -1.101 | 0.556 | -0.126 | 0.623 |
| ED_+3_ | Anterior corona radiata L | 0.184 | 0.967 | -0.002 | 0.999 |
| ED_+3_ | Superior corona radiata R | -0.595 | 0.832 | -0.065 | 0.781 |
| ED_+3_ | Superior corona radiata L | 0.216 | 0.967 | 0.046 | 0.851 |
| ED_+3_ | Posterior corona radiata R | -0.156 | 0.967 | -0.016 | 0.967 |
| ED_+3_ | Posterior corona radiata L | -1.370 | 0.428 | -0.104 | 0.675 |
| ED_+3_ | Posterior thalamic radiation R | -0.421 | 0.867 | -0.081 | 0.738 |
| ED_+3_ | Posterior thalamic radiation L | -0.194 | 0.967 | -0.030 | 0.923 |
| ED_+3_ | Sagittal stratum R | -1.415 | 0.428 | -0.166 | 0.504 |
| ED_+3_ | Sagittal stratum L | 1.206 | 0.532 | 0.100 | 0.682 |
| ED_+3_ | External capsule R | -0.940 | 0.630 | -0.125 | 0.623 |
| ED_+3_ | External capsule L | 0.251 | 0.967 | 0.059 | 0.796 |
| ED_+3_ | Cingulum (cingulate gyrus) R | -2.968 | 0.173 | -0.277 | 0.267 |
| ED_+3_ | Cingulum (cingulate gyrus) L | -1.956 | 0.192 | -0.199 | 0.416 |
| ED_+3_ | Cingulum (hippocampus) R | -1.052 | 0.575 | -0.131 | 0.617 |
| ED_+3_ | Cingulum (hippocampus) L | -2.759 | 0.173 | -0.264 | 0.296 |
| ED_+3_ | Fornix (cres)/Stria terminalis R | -0.191 | 0.967 | -0.048 | 0.846 |
| ED_+3_ | Fornix (cres)/Stria terminalis L | -0.525 | 0.832 | -0.032 | 0.914 |
| ED_+3_ | Superior longitudinal fasciculus R | 0.064 | 0.967 | 0.036 | 0.894 |
| ED_+3_ | Superior longitudinal fasciculus L | 0.542 | 0.832 | 0.063 | 0.784 |
| ED_+3_ | Superior fronto-occipital fasciculus R | 0.041 | 0.967 | -0.093 | 0.707 |
| ED_+3_ | Superior fronto-occipital fasciculus L | 1.127 | 0.556 | 0.104 | 0.675 |
| ED_+3_ | Inferior fronto-occipital fasciculus R | -1.426 | 0.428 | -0.178 | 0.466 |
| ED_+3_ | Inferior fronto-occipital fasciculus L | 0.061 | 0.967 | -0.002 | 0.999 |
| ED_+3_ | Uncinate fasciculus R | -2.162 | 0.181 | -0.223 | 0.404 |
| ED_+3_ | Uncinate fasciculus L | -0.860 | 0.659 | -0.092 | 0.707 |
| ED_+3_ | Tapetum R | -0.510 | 0.832 | -0.079 | 0.743 |
| ED_+3_ | Tapetum L | -0.945 | 0.630 | -0.053 | 0.820 |
| ED_+5_ | Middle cerebellar peduncle | -1.587 | 0.446 | -0.180 | 0.466 |
| ED_+5_ | Pontine crossing tract | -1.616 | 0.446 | -0.279 | 0.267 |
| ED_+5_ | Genu of corpus callosum | 0.063 | 0.978 | -0.005 | 0.999 |
| ED_+5_ | Body of corpus callosum | 0.053 | 0.978 | -0.012 | 0.982 |
| ED_+5_ | Splenium of corpus callosum | -1.247 | 0.526 | -0.092 | 0.707 |
| ED_+5_ | Fornix (column and body of fornix) | -0.648 | 0.726 | -0.225 | 0.404 |
| ED_+5_ | Corticospinal tract R | -1.287 | 0.523 | -0.134 | 0.607 |
| ED_+5_ | Corticospinal tract L | -0.861 | 0.683 | -0.156 | 0.524 |
| ED_+5_ | Medial lemniscus R | -1.716 | 0.446 | -0.241 | 0.362 |
| ED_+5_ | Medial lemniscus L | -2.060 | 0.446 | -0.245 | 0.356 |
| ED_+5_ | Inferior cerebellar peduncle R | -1.292 | 0.523 | -0.140 | 0.599 |
| ED_+5_ | Inferior cerebellar peduncle L | -0.512 | 0.746 | -0.139 | 0.600 |
| ED_+5_ | Superior cerebellar peduncle R | -1.583 | 0.446 | -0.225 | 0.404 |
| ED_+5_ | Superior cerebellar peduncle L | -1.938 | 0.446 | -0.237 | 0.362 |
| ED_+5_ | Cerebral peduncle R | 0.974 | 0.655 | 0.123 | 0.623 |
| ED_+5_ | Cerebral peduncle L | -0.749 | 0.726 | -0.227 | 0.404 |
| ED_+5_ | Anterior limb of internal capsule R | 2.272 | 0.446 | 0.076 | 0.756 |
| ED_+5_ | Anterior limb of internal capsule L | -1.436 | 0.469 | -0.194 | 0.421 |
| ED_+5_ | Posterior limb of internal capsule R | 1.445 | 0.469 | 0.069 | 0.772 |
| ED_+5_ | Posterior limb of internal capsule L | -0.628 | 0.726 | -0.234 | 0.362 |
| ED_+5_ | Retrolenticular part of internal capsule R | 1.188 | 0.526 | 0.107 | 0.665 |
| ED_+5_ | Retrolenticular part of internal capsule L | -0.241 | 0.901 | -0.001 | 0.999 |
| ED_+5_ | Anterior corona radiata R | 2.720 | 0.411 | 0.205 | 0.416 |
| ED_+5_ | Anterior corona radiata L | -0.941 | 0.655 | -0.179 | 0.466 |
| ED_+5_ | Superior corona radiata R | 0.539 | 0.742 | 0.092 | 0.707 |
| ED_+5_ | Superior corona radiata L | -0.178 | 0.935 | 0.003 | 0.999 |
| ED_+5_ | Posterior corona radiata R | -0.623 | 0.726 | -0.057 | 0.806 |
| ED_+5_ | Posterior corona radiata L | -0.275 | 0.901 | -0.010 | 0.990 |
| ED_+5_ | Posterior thalamic radiation R | -1.341 | 0.523 | -0.129 | 0.617 |
| ED_+5_ | Posterior thalamic radiation L | -1.201 | 0.526 | -0.122 | 0.626 |
| ED_+5_ | Sagittal stratum R | 0.984 | 0.655 | 0.094 | 0.707 |
| ED_+5_ | Sagittal stratum L | -0.572 | 0.737 | -0.178 | 0.466 |
| ED_+5_ | External capsule R | 1.761 | 0.446 | 0.099 | 0.685 |
| ED_+5_ | External capsule L | 0.563 | 0.737 | -0.101 | 0.682 |
| ED_+5_ | Cingulum (cingulate gyrus) R | -0.715 | 0.726 | -0.065 | 0.781 |
| ED_+5_ | Cingulum (cingulate gyrus) L | -0.686 | 0.726 | -0.087 | 0.723 |
| ED_+5_ | Cingulum (hippocampus) R | -0.657 | 0.726 | -0.099 | 0.685 |
| ED_+5_ | Cingulum (hippocampus) L | -1.852 | 0.446 | -0.226 | 0.404 |
| ED_+5_ | Fornix (cres)/Stria terminalis R | 1.557 | 0.446 | 0.068 | 0.772 |
| ED_+5_ | Fornix (cres)/Stria terminalis L | 0.054 | 0.978 | -0.102 | 0.680 |
| ED_+5_ | Superior longitudinal fasciculus R | 0.947 | 0.655 | 0.142 | 0.591 |
| ED_+5_ | Superior longitudinal fasciculus L | -0.258 | 0.901 | -0.074 | 0.761 |
| ED_+5_ | Superior fronto-occipital fasciculus R | 2.256 | 0.446 | 0.078 | 0.745 |
| ED_+5_ | Superior fronto-occipital fasciculus L | 0.854 | 0.683 | -0.019 | 0.957 |
| ED_+5_ | Inferior fronto-occipital fasciculus R | 1.895 | 0.446 | 0.139 | 0.600 |
| ED_+5_ | Inferior fronto-occipital fasciculus L | -0.014 | 0.989 | -0.196 | 0.416 |
| ED_+5_ | Uncinate fasciculus R | 1.848 | 0.446 | 0.158 | 0.524 |
| ED_+5_ | Uncinate fasciculus L | -0.346 | 0.870 | -0.187 | 0.449 |
| ED_+5_ | Tapetum R | -0.715 | 0.726 | -0.082 | 0.738 |
| ED_+5_ | Tapetum L | -1.446 | 0.469 | -0.198 | 0.416 |

P-values were adjusted for multiple comparisons using Benjamini-Hochberg’s false discovery rate (FDR) correction method.

**Supplemental table 3:** Comparison of correlation coefficients across different thresholds (ED_+3_ versus ED_+5_ and ED_-3_ versus ED_-5_ respectively) using Pearson’s 1898 method of comparing correlation coefficients across different age groups. P-values were adjusted for multiple comparisons using Benjamini-Hochberg’s false discovery rate (FDR) correction method. *Abbreviations: ASD = autism spectrum disorder, ED = edge density, FDR = false discovery rate, L = left, R = right*

| **3.a. Infants** | **ED_+3_ versus ED_+5_** | | **ED-_3_ versus ED-_5_** | |
| --- | --- | --- | --- | --- |
| Tract | **z** | **p_FDR_** | **z** | **p_FDR_** |
| Middle cerebellar peduncle | -0.7 | 0.605 | 1.156 | 0.711 |
| Pontine crossing tract | -1.148 | 0.359 | 1.357 | 0.686 |
| Genu of corpus callosum | -2.244 | 0.052 | 1.539 | 0.686 |
| Body of corpus callosum | 5.268 | **<0.001** | 1.353 | 0.686 |
| Splenium of corpus callosum | -6.413 | **<0.001** | 1.474 | 0.686 |
| Fornix (column and body of fornix) | 4.749 | **<0.001** | -1.346 | 0.686 |
| Corticospinal tract R | -2.041 | 0.076 | 1.875 | 0.608 |
| Corticospinal tract L | -0.687 | 0.605 | 0.714 | 0.816 |
| Medial lemniscus R | -0.052 | 0.968 | 1.069 | 0.712 |
| Medial lemniscus L | 0.269 | 0.82 | 0.696 | 0.816 |
| Inferior cerebellar peduncle R | -0.538 | 0.656 | 0.187 | 0.88 |
| Inferior cerebellar peduncle L | -0.948 | 0.464 | 0.516 | 0.816 |
| Superior cerebellar peduncle R | -0.407 | 0.744 | 0.474 | 0.816 |
| Superior cerebellar peduncle L | -0.642 | 0.605 | 0.293 | 0.88 |
| Cerebral peduncle R | -3.526 | **0.001** | 0.87 | 0.816 |
| Cerebral peduncle L | 4.992 | **<0.001** | 0.658 | 0.816 |
| Anterior limb of internal capsule R | 2.198 | 0.056 | -1.136 | 0.711 |
| Anterior limb of internal capsule L | -3.54 | **0.001** | -0.213 | 0.88 |
| Posterior limb of internal capsule R | 1.574 | 0.186 | -0.446 | 0.816 |
| Posterior limb of internal capsule L | 1.648 | 0.171 | 1.213 | 0.703 |
| Retrolenticular part of internal capsule R | -3.179 | **0.004** | -2.12 | 0.475 |
| Retrolenticular part of internal capsule L | 1.817 | 0.124 | 0.41 | 0.816 |
| Anterior corona radiata R | -4.179 | **<0.001** | -1.568 | 0.686 |
| Anterior corona radiata L | -4.134 | **<0.001** | 0.485 | 0.816 |
| Superior corona radiata R | 2.857 | **0.011** | -0.44 | 0.816 |
| Superior corona radiata L | 1.597 | 0.184 | 0.225 | 0.88 |
| Posterior corona radiata R | -4.49 | **<0.001** | -1.438 | 0.686 |
| Posterior corona radiata L | -0.351 | 0.772 | 0.625 | 0.816 |
| Posterior thalamic radiation R | 6.666 | **<0.001** | 0.66 | 0.816 |
| Posterior thalamic radiation L | -1.038 | 0.416 | 0.453 | 0.816 |
| Sagittal stratum R | 0.636 | 0.605 | -1.612 | 0.686 |
| Sagittal stratum L | 4.504 | **<0.001** | -0.455 | 0.816 |
| External capsule R | 0.624 | 0.605 | -2.112 | 0.475 |
| External capsule L | -1.274 | 0.298 | -0.708 | 0.816 |
| Cingulum (cingulate gyrus) R | -3.307 | **0.003** | -0.438 | 0.816 |
| Cingulum (cingulate gyrus) L | 4.035 | **<0.001** | 2.2 | 0.475 |
| Cingulum (hippocampus) R | 3.559 | **0.001** | -2.075 | 0.475 |
| Cingulum (hippocampus) L | 1.553 | 0.188 | -0.228 | 0.88 |
| Fornix (cres)/Stria terminalis R | -0.637 | 0.605 | -1.213 | 0.703 |
| Fornix (cres)/Stria terminalis L | 6.243 | **<0.001** | 0.406 | 0.816 |
| Superior longitudinal fasciculus R | -2.813 | **0.012** | -0.817 | 0.816 |
| Superior longitudinal fasciculus L | -1.399 | 0.245 | 0.007 | 0.994 |
| Superior fronto-occipital fasciculus R R | 2.148 | 0.061 | -1.09 | 0.712 |
| Superior fronto-occipital fasciculus R L | 4.525 | **<0.001** | -0.629 | 0.816 |
| Inferior fronto-occipital fasciculus R | -4.173 | **<0.001** | -1.217 | 0.703 |
| Inferior fronto-occipital fasciculus L | 2.42 | **0.035** | -0.173 | 0.88 |
| Uncinate fasciculus R | -2.266 | 0.051 | -0.525 | 0.816 |
| Uncinate fasciculus L | -0.771 | 0.58 | -0.218 | 0.88 |
| Tapetum R | 0.04 | 0.968 | -0.995 | 0.761 |
| Tapetum L | -3.073 | **0.006** | -0.888 | 0.816 |

| **3.b. Toddlers** | **ED_+3_ versus ED_+5_** | | **ED-_3_ versus ED-_5_** | |
| --- | --- | --- | --- | --- |
| Tract | **z** | **p_FDR_** | **z** | **p_FDR_** |
| Middle cerebellar peduncle | 1.988 | 0.209 | 1.261 | 0.826 |
| Pontine crossing tract | 2.095 | 0.206 | -0.520 | 0.944 |
| Genu of corpus callosum | 1.253 | 0.422 | -0.607 | 0.944 |
| Body of corpus callosum | 2.297 | 0.157 | -0.166 | 0.963 |
| Splenium of corpus callosum | 0.920 | 0.551 | 1.164 | 0.826 |
| Fornix (column and body of fornix) | 1.678 | 0.290 | 0.524 | 0.944 |
| Corticospinal tract R | 2.362 | 0.145 | 0.189 | 0.963 |
| Corticospinal tract L | 2.549 | 0.127 | 0.577 | 0.944 |
| Medial lemniscus R | 2.425 | 0.140 | -0.902 | 0.856 |
| Medial lemniscus L | 2.235 | 0.174 | -0.829 | 0.891 |
| Inferior cerebellar peduncle R | 2.430 | 0.140 | 1.104 | 0.826 |
| Inferior cerebellar peduncle L | 2.800 | 0.102 | 0.901 | 0.856 |
| Superior cerebellar peduncle R | 2.656 | 0.115 | -0.548 | 0.944 |
| Superior cerebellar peduncle L | 3.03 | 0.07 | 0.601 | 0.944 |
| Cerebral peduncle R | 1.634 | 0.309 | 0.518 | 0.944 |
| Cerebral peduncle L | 2.048 | 0.209 | 0.425 | 0.944 |
| Anterior limb of internal capsule R | 2.914 | 0.079 | -0.094 | 0.976 |
| Anterior limb of internal capsule L | 1.481 | 0.355 | -1.368 | 0.824 |
| Posterior limb of internal capsule R | 1.615 | 0.316 | -0.426 | 0.944 |
| Posterior limb of internal capsule L | 2.12 | 0.201 | 0.149 | 0.963 |
| Retrolenticular part of internal capsule R | 0.926 | 0.551 | -0.211 | 0.963 |
| Retrolenticular part of internal capsule L | 1.345 | 0.386 | 0.599 | 0.944 |
| Anterior corona radiata R | 1.519 | 0.347 | -0.96 | 0.856 |
| Anterior corona radiata L | 1.295 | 0.409 | -0.373 | 0.946 |
| Superior corona radiata R | 1.643 | 0.308 | -0.33 | 0.955 |
| Superior corona radiata L | 1.786 | 0.270 | 0.65 | 0.944 |
| Posterior corona radiata R | 0.86 | 0.582 | -0.33 | 0.955 |
| Posterior corona radiata L | 1.702 | 0.290 | 0.093 | 0.976 |
| Posterior thalamic radiation R | 0.845 | 0.582 | 0.397 | 0.944 |
| Posterior thalamic radiation L | 1.517 | 0.347 | 1.666 | 0.817 |
| Sagittal stratum R | 2.014 | 0.209 | 0.614 | 0.944 |
| Sagittal stratum L | 1.035 | 0.522 | 0.862 | 0.87 |
| External capsule R | 1.771 | 0.272 | -0.217 | 0.963 |
| External capsule L | 0.686 | 0.667 | 1.289 | 0.826 |
| Cingulum (cingulate gyrus) R | 1.176 | 0.472 | 0.659 | 0.944 |
| Cingulum (cingulate gyrus) L | 0.976 | 0.524 | -0.764 | 0.917 |
| Cingulum (hippocampus) R | -0.301 | 0.872 | -0.361 | 0.946 |
| Cingulum (hippocampus) L | 0.719 | 0.649 | 0.376 | 0.946 |
| Fornix (cres)/Stria terminalis R | 0.793 | 0.612 | 0.402 | 0.944 |
| Fornix (cres)/Stria terminalis L | -0.499 | 0.771 | 0.283 | 0.955 |
| Superior longitudinal fasciculus R | 0.313 | 0.868 | -1.14 | 0.826 |
| Superior longitudinal fasciculus L | 0.936 | 0.547 | -2.561 | 0.633 |
| Superior fronto-occipital fasciculus R R | 4.166 | **0.006** | 0.984 | 0.856 |
| Superior fronto-occipital fasciculus R L | 1.679 | 0.290 | 0.493 | 0.944 |
| Inferior fronto-occipital fasciculus R | 2.051 | 0.209 | 0.160 | 0.963 |
| Inferior fronto-occipital fasciculus L | 1.570 | 0.331 | 0.651 | 0.944 |
| Uncinate fasciculus R | 2.165 | 0.195 | 0.286 | 0.955 |
| Uncinate fasciculus L | 1.115 | 0.493 | 0.112 | 0.976 |
| Tapetum R | 1.138 | 0.479 | 0.335 | 0.955 |
| Tapetum L | 0.100 | 0.974 | -0.316 | 0.955 |

| **3.c. Adolescents** | **ED_+3_ versus ED_+5_** | | **ED-_3_ versus ED-_5_** | |
| --- | --- | --- | --- | --- |
| Tracts | **z** | **p_FDR_** | **z** | **p_FDR_** |
| Middle cerebellar peduncle | -0.452 | 0.805 | -0.471 | 0.944 |
| Pontine crossing tract | -1.845 | 0.254 | -0.399 | 0.944 |
| Genu of corpus callosum | -1.014 | 0.522 | 0.945 | 0.856 |
| Body of corpus callosum | -0.989 | 0.522 | 1.504 | 0.817 |
| Splenium of corpus callosum | -1.412 | 0.366 | 0.313 | 0.955 |
| Fornix (column and body of fornix) | 0.123 | 0.971 | 1.107 | 0.826 |
| Corticospinal tract R | -0.574 | 0.731 | -0.389 | 0.944 |
| Corticospinal tract L | 0.659 | 0.677 | 1.360 | 0.824 |
| Medial lemniscus R | -2.691 | 0.115 | -1.178 | 0.826 |
| Medial lemniscus L | -1.989 | 0.209 | 0.909 | 0.856 |
| Inferior cerebellar peduncle R | -1.045 | 0.591 | 1.499 | 0.837 |
| Inferior cerebellar peduncle L | -0.101 | 0.974 | 2.089 | 0.817 |
| Superior cerebellar peduncle R | -1.873 | 0.243 | 0.017 | 0.999 |
| Superior cerebellar peduncle L | -2.427 | 0.140 | 1.442 | 0.824 |
| Cerebral peduncle R | -1.082 | 0.505 | -0.570 | 0.944 |
| Cerebral peduncle L | 1.396 | 0.368 | 1.570 | 0.817 |
| Anterior limb of internal capsule R | -1.977 | 0.209 | -0.276 | 0.955 |
| Anterior limb of internal capsule L | -1.487 | 0.355 | 0.618 | 0.944 |
| Posterior limb of internal capsule R | -0.371 | 0.841 | -0.070 | 0.984 |
| Posterior limb of internal capsule L | 0.036 | 0.991 | 0.792 | 0.917 |
| Retrolenticular part of internal capsule R | -2.475 | 0.140 | 0.400 | 0.944 |
| Retrolenticular part of internal capsule L | -0.012 | 0.995 | 1.479 | 0.817 |
| Anterior corona radiata R | -2.363 | 0.145 | -0.105 | 0.976 |
| Anterior corona radiata L | -1.961 | 0.210 | 0.030 | 0.996 |
| Superior corona radiata R | -1.415 | 0.366 | -0.155 | 0.963 |
| Superior corona radiata L | -1.165 | 0.476 | -0.585 | 0.944 |
| Posterior corona radiata R | -1.691 | 0.290 | 1.115 | 0.826 |
| Posterior corona radiata L | -1.43 | 0.362 | 1.361 | 0.824 |
| Posterior thalamic radiation R | -2.126 | 0.201 | -1.108 | 0.826 |
| Posterior thalamic radiation L | -0.296 | 0.872 | 1.061 | 0.84 |
| Sagittal stratum R | -1.702 | 0.290 | -0.394 | 0.944 |
| Sagittal stratum L | 0.082 | 0.974 | 1.081 | 0.831 |
| External capsule R | -2.04 | 0.209 | 0.002 | 0.999 |
| External capsule L | -1.807 | 0.270 | 1.522 | 0.817 |
| Cingulum (cingulate gyrus) R | -1.976 | 0.209 | 1.043 | 0.844 |
| Cingulum (cingulate gyrus) L | -0.622 | 0.699 | 0.191 | 0.963 |
| Cingulum (hippocampus) R | -0.853 | 0.582 | -0.921 | 0.856 |
| Cingulum (hippocampus) L | 1.150 | 0.479 | 2.491 | 0.633 |
| Fornix (cres)/Stria terminalis R | -0.904 | 0.555 | 0.437 | 0.944 |
| Fornix (cres)/Stria terminalis L | 0.006 | 0.995 | 0.324 | 0.955 |
| Superior longitudinal fasciculus R | -1.989 | 0.209 | -0.996 | 0.856 |
| Superior longitudinal fasciculus L | -0.976 | 0.524 | 0.368 | 0.946 |
| Superior fronto-occipital fasciculus R R | -2.053 | 0.209 | -1.677 | 0.817 |
| Superior fronto-occipital fasciculus R L | -1.452 | 0.355 | -1.294 | 0.826 |
| Inferior fronto-occipital fasciculus R | -2.289 | 0.157 | 0.086 | 0.976 |
| Inferior fronto-occipital fasciculus L | -0.741 | 0.638 | 1.477 | 0.817 |
| Uncinate fasciculus R | -1.719 | 0.290 | 0.203 | 0.963 |
| Uncinate fasciculus L | -0.16 | 0.955 | 1.711 | 0.817 |
| Tapetum R | -1.95 | 0.210 | -1.055 | 0.84 |
| Tapetum L | -1.109 | 0.493 | 0.005 | 0.999 |

| **3.d. Adults** | **ED_+3_ versus ED_+5_** | | **ED-_3_ versus ED-_5_** | |
| --- | --- | --- | --- | --- |
| **Tracts** | **z** | **p_FDR_** | **z** | **p_FDR_** |
| Middle cerebellar peduncle | -1.454 | 0.355 | -0.434 | 0.944 |
| Pontine crossing tract | -0.821 | 0.598 | 0.753 | 0.917 |
| Genu of corpus callosum | -2.117 | 0.201 | 0.055 | 0.986 |
| Body of corpus callosum | -1.585 | 0.329 | 0.955 | 0.856 |
| Splenium of corpus callosum | -0.781 | 0.613 | -1.527 | 0.817 |
| Fornix (column and body of fornix) | -0.088 | 0.974 | -0.461 | 0.944 |
| Corticospinal tract R | -0.782 | 0.613 | -0.634 | 0.944 |
| Corticospinal tract L | -0.43 | 0.814 | 0.927 | 0.856 |
| Medial lemniscus R | -1.244 | 0.425 | -1.73 | 0.817 |
| Medial lemniscus L | 0.344 | 0.859 | -1.438 | 0.824 |
| Inferior cerebellar peduncle R | 0.444 | 0.807 | -1.126 | 0.826 |
| Inferior cerebellar peduncle L | -0.047 | 0.987 | -0.877 | 0.87 |
| Superior cerebellar peduncle R | -1.086 | 0.505 | 0.598 | 0.944 |
| Superior cerebellar peduncle L | 0.124 | 0.971 | 0.293 | 0.955 |
| Cerebral peduncle R | -3.283 | 0.048 | -1.831 | 0.817 |
| Cerebral peduncle L | -0.9 | 0.555 | -0.512 | 0.944 |
| Anterior limb of internal capsule R | -3.237 | 0.048 | -0.541 | 0.944 |
| Anterior limb of internal capsule L | 0.712 | 0.649 | 1.707 | 0.817 |
| Posterior limb of internal capsule R | -1.68 | 0.29 | -1.103 | 0.826 |
| Posterior limb of internal capsule L | 0.319 | 0.867 | -0.22 | 0.963 |
| Retrolenticular part of internal capsule R | -2.297 | 0.157 | -0.678 | 0.944 |
| Retrolenticular part of internal capsule L | 0.122 | 0.971 | -1.114 | 0.826 |
| Anterior corona radiata R | -2.648 | 0.115 | 0.849 | 0.875 |
| Anterior corona radiata L | 1.286 | 0.411 | 1.209 | 0.826 |
| Superior corona radiata R | -1.52 | 0.347 | 0.278 | 0.955 |
| Superior corona radiata L | 0.386 | 0.839 | 0.175 | 0.963 |
| Posterior corona radiata R | 0.523 | 0.767 | -1.768 | 0.817 |
| Posterior corona radiata L | -0.999 | 0.522 | 1.702 | 0.817 |
| Posterior thalamic radiation R | 1.369 | 0.382 | -1.867 | 0.817 |
| Posterior thalamic radiation L | 1.064 | 0.51 | -1.157 | 0.826 |
| Sagittal stratum R | -3.036 | 0.07 | -1.157 | 0.826 |
| Sagittal stratum L | 1.138 | 0.479 | -2.896 | 0.633 |
| External capsule R | -2.967 | 0.075 | 0.223 | 0.963 |
| External capsule L | -0.111 | 0.974 | -1.087 | 0.831 |
| Cingulum (cingulate gyrus) R | -2.224 | 0.174 | -0.253 | 0.963 |
| Cingulum (cingulate gyrus) L | -1.007 | 0.522 | 0.574 | 0.944 |
| Cingulum (hippocampus) R | -0.335 | 0.859 | -0.178 | 0.963 |
| Cingulum (hippocampus) L | -0.399 | 0.832 | -1.698 | 0.817 |
| Fornix (cres)/Stria terminalis R | -1.58 | 0.329 | 0.106 | 0.976 |
| Fornix (cres)/Stria terminalis L | -0.496 | 0.771 | -1.585 | 0.817 |
| Superior longitudinal fasciculus R | -1.4 | 0.368 | -2.685 | 0.633 |
| Superior longitudinal fasciculus L | 0.717 | 0.649 | 2.012 | 0.817 |
| Superior fronto-occipital fasciculus R R | -1.467 | 0.355 | 1.647 | 0.817 |
| Superior fronto-occipital fasciculus R L | 0.503 | 0.771 | 1.188 | 0.826 |
| Inferior fronto-occipital fasciculus R | -3.505 | 0.03 | -1.319 | 0.826 |
| Inferior fronto-occipital fasciculus L | 0.06 | 0.982 | -0.599 | 0.944 |
| Uncinate fasciculus R | -4.018 | 0.006 | -2.322 | 0.806 |
| Uncinate fasciculus L | -0.515 | 0.769 | -1.526 | 0.817 |
| Tapetum R | 0.189 | 0.945 | -1.408 | 0.824 |
| Tapetum L | 0.336 | 0.859 | -0.407 | 0.944 |

P-values were adjusted for multiple comparisons using Benjamini-Hochberg’s false discovery rate (FDR) correction method.

**Supplemental table 4** Results from previous study, where edge density was created by guiding tractography through anatomically defined nodes (7). As we applied termination masks in this approach, several tracts that had an overlap with those masks were not analyzed (Pontine crossing fibres, L/R corticospinal tracts, L/R medical lemnisci, L/R Inferior fronto-occipital fasciculi). *Abbreviations: ASD = autism spectrum disorder, ED = edge density, FDR = false discovery rate, L = left, R = right*

| **4.a Infants** | | | | |
| --- | --- | --- | --- | --- |
| Tract | t-value | p_FDR_ | Spearman’s Rho | P_FDR_ |
| Middle cerebellar peduncle | 0.358 | 0.914 | -0.279 | 0.087 |
| Genu of corpus callosum | 0.962 | 0.633 | -0.053 | 0.755 |
| Body of corpus callosum | -1.139 | 0.568 | 0.211 | 0.198 |
| Splenium of corpus callosum | 0.356 | 0.914 | 0.012 | 0.95 |
| Fornix (column and body of fornix) | 0.3 | 0.933 | -0.002 | 0.993 |
| Inferior cerebellar peduncle R | 0.405 | 0.914 | -0.102 | 0.603 |
| Inferior cerebellar peduncle L | 0.865 | 0.691 | -0.133 | 0.479 |
| Superior cerebellar peduncle R | 0.398 | 0.914 | -0.094 | 0.651 |
| Superior cerebellar peduncle L | -0.335 | 0.925 | -0.011 | 0.95 |
| Cerebral peduncle R | -0.428 | 0.914 | -0.009 | 0.95 |
| Cerebral peduncle L | 0.362 | 0.914 | -0.021 | 0.901 |
| Anterior limb of internal capsule R | -0.573 | 0.842 | 0.128 | 0.509 |
| Anterior limb of internal capsule L | -0.478 | 0.893 | 0.091 | 0.658 |
| Posterior limb of internal capsule R | 0.747 | 0.765 | -0.009 | 0.95 |
| Posterior limb of internal capsule L | 0.382 | 0.914 | 0.087 | 0.669 |
| Retrolenticular part of internal capsule R | 1.094 | 0.588 | -0.024 | 0.889 |
| Retrolenticular part of internal capsule L | 1.571 | 0.405 | -0.096 | 0.649 |
| Anterior corona radiata R | 0.494 | 0.893 | 0 | 0.997 |
| Anterior corona radiata L | 0.048 | 0.985 | 0.081 | 0.679 |
| Superior corona radiata R | 0.145 | 0.964 | 0.118 | 0.556 |
| Superior corona radiata L | 0.633 | 0.826 | 0.025 | 0.889 |
| Posterior corona radiata R | 0.636 | 0.826 | -0.039 | 0.82 |
| Posterior corona radiata L | 0.154 | 0.964 | 0.05 | 0.776 |
| Posterior thalamic radiation R | 0.33 | 0.925 | 0.067 | 0.724 |
| Posterior thalamic radiation L | 0.177 | 0.964 | 0.072 | 0.704 |
| Sagittal stratum R | -0.17 | 0.964 | 0.089 | 0.664 |
| Sagittal stratum L | 0.005 | 0.997 | 0.057 | 0.739 |
| External capsule R | 0.054 | 0.985 | 0.117 | 0.561 |
| External capsule L | 0.989 | 0.622 | -0.017 | 0.924 |
| Cingulum (cingulate gyrus) R | 1.324 | 0.532 | -0.059 | 0.732 |
| Cingulum (cingulate gyrus) L | 0.26 | 0.937 | 0.037 | 0.82 |
| Cingulum (hippocampus) R | 0.086 | 0.971 | 0.01 | 0.95 |
| Cingulum (hippocampus) L | -0.269 | 0.937 | 0.071 | 0.704 |
| Fornix (cres)/Stria terminalis R | 0.102 | 0.97 | 0.079 | 0.679 |
| Fornix (cres)/Stria terminalis L | 0.122 | 0.964 | 0.06 | 0.732 |
| Superior longitudinal fasciculus R | 1.248 | 0.542 | -0.057 | 0.739 |
| Superior longitudinal fasciculus L | 2.126 | 0.214 | -0.168 | 0.339 |
| Superior fronto-occipital fasciculus R | -0.116 | 0.964 | 0.061 | 0.731 |
| Superior fronto-occipital fasciculus L | -0.993 | 0.622 | 0.09 | 0.658 |
| Uncinate fasciculus R | -0.272 | 0.937 | 0.092 | 0.658 |
| Uncinate fasciculus L | -0.12 | 0.964 | 0.079 | 0.679 |
| Tapetum R | 1.248 | 0.542 | -0.086 | 0.669 |
| Tapetum L | -0.369 | 0.914 | 0.061 | 0.732 |

| **4.b Toddlers** | | | | |
| --- | --- | --- | --- | --- |
| tract | t-values | p_FDR_ | Spearman's Rho | p_FDR_ |
| Middle cerebellar peduncle | -1.005 | 0.622 | 0.025 | 0.82 |
| Genu of corpus callosum | 0.483 | 0.893 | -0.025 | 0.82 |
| Body of corpus callosum | 2.556 | 0.149 | -0.153 | 0.16 |
| Splenium of corpus callosum | 3.364 | 0.255 | -0.213 | 0.209 |
| Fornix (column and body of fornix) | 2.252 | 0.189 | -0.149 | 0.168 |
| Inferior cerebellar peduncle R | -0.425 | 0.914 | 0.052 | 0.679 |
| Inferior cerebellar peduncle L | -0.573 | 0.842 | 0.035 | 0.755 |
| Superior cerebellar peduncle R | -0.547 | 0.86 | 0.014 | 0.901 |
| Superior cerebellar peduncle L | -0.645 | 0.826 | 0.03 | 0.806 |
| Cerebral peduncle R | 1.821 | 0.322 | -0.111 | 0.339 |
| Cerebral peduncle L | 0.594 | 0.841 | -0.02 | 0.861 |
| Anterior limb of internal capsule R | 1.662 | 0.392 | -0.094 | 0.454 |
| Anterior limb of internal capsule L | 2.691 | 0.12 | -0.186 | 0.087 |
| Posterior limb of internal capsule R | 1.157 | 0.566 | -0.07 | 0.599 |
| Posterior limb of internal capsule L | 1.115 | 0.572 | -0.082 | 0.522 |
| Retrolenticular part of internal capsule R | 1.719 | 0.365 | -0.1 | 0.4 |
| Retrolenticular part of internal capsule L | 1.993 | 0.255 | -0.132 | 0.229 |
| Anterior corona radiata R | 1.813 | 0.322 | -0.111 | 0.339 |
| Anterior corona radiata L | 2.501 | 0.15 | -0.167 | 0.126 |
| Superior corona radiata R | 2.424 | 0.162 | -0.148 | 0.168 |
| Superior corona radiata L | 2.28 | 0.189 | -0.145 | 0.183 |
| Posterior corona radiata R | 2.855 | 0.09 | -0.171 | 0.117 |
| Posterior corona radiata L | 3.351 | 0.937 | -0.081 | 0.679 |
| Posterior thalamic radiation R | 2.328 | 0.188 | -0.156 | 0.16 |
| Posterior thalamic radiation L | 1.28 | 0.542 | -0.053 | 0.679 |
| Sagittal stratum R | 1.331 | 0.529 | -0.127 | 0.247 |
| Sagittal stratum L | 1.438 | 0.466 | -0.104 | 0.371 |
| External capsule R | 1.599 | 0.405 | -0.086 | 0.502 |
| External capsule L | 1.591 | 0.405 | -0.105 | 0.371 |
| Cingulum (cingulate gyrus) R | 2.015 | 0.25 | -0.156 | 0.16 |
| Cingulum (cingulate gyrus) L | 2.114 | 0.214 | -0.134 | 0.221 |
| Cingulum (hippocampus) R | 2.14 | 0.213 | -0.114 | 0.339 |
| Cingulum (hippocampus) L | -0.77 | 0.753 | 0.027 | 0.82 |
| Fornix (cres)/Stria terminalis R | 0.801 | 0.736 | -0.068 | 0.603 |
| Fornix (cres)/Stria terminalis L | 1.561 | 0.405 | -0.121 | 0.289 |
| Superior longitudinal fasciculus R | 2.198 | 0.196 | -0.171 | 0.117 |
| Superior longitudinal fasciculus L | 2.57 | 0.149 | -0.187 | 0.087 |
| Superior fronto-occipital fasciculus R | 1.743 | 0.359 | -0.096 | 0.43 |
| Superior fronto-occipital fasciculus L | 2.855 | 0.09 | -0.182 | 0.087 |
| Uncinate fasciculus R | 0.087 | 0.971 | -0.051 | 0.693 |
| Uncinate fasciculus L | 1.013 | 0.622 | -0.091 | 0.463 |
| Tapetum R | 1.929 | 0.273 | -0.106 | 0.37 |
| Tapetum L | 3.105 | 0.053 | -0.184 | 0.087 |

| **4.c Adolescents** | | | | |
| --- | --- | --- | --- | --- |
| tract | t-values | p_FDR_ | Spearman's Rho | p_FDR_ |
| Middle cerebellar peduncle | 1.918 | 0.276 | -0.09 | 0.591 |
| Genu of corpus callosum | -1.009 | 0.622 | 0.084 | 0.599 |
| Body of corpus callosum | -1.434 | 0.477 | 0.122 | 0.4 |
| Splenium of corpus callosum | -0.031 | 0.992 | 0.03 | 0.82 |
| Fornix (column and body of fornix) | 0.508 | 0.886 | -0.056 | 0.708 |
| Inferior cerebellar peduncle R | 0.267 | 0.937 | -0.031 | 0.82 |
| Inferior cerebellar peduncle L | 1.155 | 0.566 | -0.051 | 0.731 |
| Superior cerebellar peduncle R | 1.649 | 0.398 | -0.159 | 0.233 |
| Superior cerebellar peduncle L | 1.281 | 0.542 | -0.148 | 0.289 |
| Cerebral peduncle R | -1.761 | 0.359 | 0.174 | 0.184 |
| Cerebral peduncle L | -0.654 | 0.826 | 0.058 | 0.704 |
| Anterior limb of internal capsule R | -1.139 | 0.568 | 0.093 | 0.567 |
| Anterior limb of internal capsule L | -0.386 | 0.914 | 0.028 | 0.823 |
| Posterior limb of internal capsule R | -0.614 | 0.832 | 0.019 | 0.889 |
| Posterior limb of internal capsule L | -0.897 | 0.69 | 0.067 | 0.679 |
| Retrolenticular part of internal capsule R | 1.543 | 0.411 | -0.112 | 0.461 |
| Retrolenticular part of internal capsule L | 4.076 | 0.011 | -0.267 | 0.033 |
| Anterior corona radiata R | -1.06 | 0.613 | 0.087 | 0.599 |
| Anterior corona radiata L | 1.002 | 0.622 | -0.076 | 0.651 |
| Superior corona radiata R | -1.267 | 0.542 | 0.092 | 0.576 |
| Superior corona radiata L | -0.313 | 0.929 | -0.028 | 0.823 |
| Posterior corona radiata R | -0.863 | 0.691 | 0.112 | 0.461 |
| Posterior corona radiata L | -0.004 | 0.997 | -0.009 | 0.95 |
| Posterior thalamic radiation R | -0.643 | 0.826 | 0.098 | 0.534 |
| Posterior thalamic radiation L | 2.226 | 0.196 | -0.186 | 0.16 |
| Sagittal stratum R | -0.124 | 0.964 | 0.032 | 0.82 |
| Sagittal stratum L | 4.036 | 0.011 | -0.261 | 0.036 |
| External capsule R | -0.242 | 0.948 | 0.053 | 0.725 |
| External capsule L | 0.164 | 0.964 | -0.047 | 0.739 |
| Cingulum (cingulate gyrus) R | -0.589 | 0.842 | 0.082 | 0.603 |
| Cingulum (cingulate gyrus) L | -1.382 | 0.502 | 0.128 | 0.371 |
| Cingulum (hippocampus) R | 0.126 | 0.964 | -0.05 | 0.731 |
| Cingulum (hippocampus) L | 0.748 | 0.765 | -0.037 | 0.806 |
| Fornix (cres)/Stria terminalis R | 0.41 | 0.914 | -0.05 | 0.731 |
| Fornix (cres)/Stria terminalis L | 0.678 | 0.826 | -0.07 | 0.669 |
| Superior longitudinal fasciculus R | -0.17 | 0.964 | 0.008 | 0.95 |
| Superior longitudinal fasciculus L | 0.89 | 0.69 | -0.087 | 0.599 |
| Superior fronto-occipital fasciculus R | -0.967 | 0.633 | 0.052 | 0.725 |
| Superior fronto-occipital fasciculus L | -0.053 | 0.985 | -0.017 | 0.901 |
| Uncinate fasciculus R | 0.64 | 0.826 | -0.058 | 0.704 |
| Uncinate fasciculus L | 1.234 | 0.542 | -0.084 | 0.599 |
| Tapetum R | 0.13 | 0.964 | 0.034 | 0.819 |
| Tapetum L | 1.944 | 0.273 | -0.102 | 0.51 |

| **4.d Adults** | | | | |
| --- | --- | --- | --- | --- |
| tract | t-values | p_FDR_ | Spearman's Rho | p_FDR_ |
| Middle cerebellar peduncle | -1.731 | 0.366 | 0.212 | 0.209 |
| Genu of corpus callosum | 0.857 | 0.691 | -0.143 | 0.461 |
| Body of corpus callosum | 1.048 | 0.613 | -0.104 | 0.603 |
| Splenium of corpus callosum | 2.01 | 0.032 | -0.234 | 0.033 |
| Fornix (column and body of fornix) | 2.593 | 0.15 | -0.235 | 0.16 |
| Inferior cerebellar peduncle R | -0.627 | 0.827 | 0.113 | 0.591 |
| Inferior cerebellar peduncle L | -0.882 | 0.69 | 0.072 | 0.704 |
| Superior cerebellar peduncle R | -0.379 | 0.914 | 0.086 | 0.679 |
| Superior cerebellar peduncle L | -1.046 | 0.613 | 0.095 | 0.657 |
| Cerebral peduncle R | 1.537 | 0.411 | -0.176 | 0.339 |
| Cerebral peduncle L | -0.192 | 0.964 | -0.038 | 0.82 |
| Anterior limb of internal capsule R | 0.382 | 0.914 | -0.073 | 0.704 |
| Anterior limb of internal capsule L | 1.19 | 0.566 | -0.129 | 0.517 |
| Posterior limb of internal capsule R | 3.268 | 0.053 | -0.307 | 0.068 |
| Posterior limb of internal capsule L | 1.637 | 0.401 | -0.167 | 0.362 |
| Retrolenticular part of internal capsule R | 2.327 | 0.189 | -0.239 | 0.16 |
| Retrolenticular part of internal capsule L | 2.243 | 0.196 | -0.255 | 0.131 |
| Anterior corona radiata R | 1.208 | 0.559 | -0.09 | 0.668 |
| Anterior corona radiata L | 1.248 | 0.542 | -0.167 | 0.362 |
| Superior corona radiata R | 1.172 | 0.566 | -0.114 | 0.591 |
| Superior corona radiata L | 1.458 | 0.466 | -0.158 | 0.399 |
| Posterior corona radiata R | 0.012 | 0.997 | -0.001 | 0.996 |
| Posterior corona radiata L | 0.266 | 0.032 | -0.231 | 0.033 |
| Posterior thalamic radiation R | 3.808 | 0.022 | -0.345 | 0.033 |
| Posterior thalamic radiation L | 2.457 | 0.162 | -0.203 | 0.23 |
| Sagittal stratum R | 1.136 | 0.568 | -0.108 | 0.599 |
| Sagittal stratum L | 0.319 | 0.929 | -0.058 | 0.74 |
| External capsule R | 1.279 | 0.542 | -0.138 | 0.479 |
| External capsule L | 1.551 | 0.411 | -0.208 | 0.221 |
| Cingulum (cingulate gyrus) R | 0.798 | 0.737 | -0.074 | 0.704 |
| Cingulum (cingulate gyrus) L | 1.579 | 0.405 | -0.175 | 0.339 |
| Cingulum (hippocampus) R | 0.116 | 0.964 | -0.006 | 0.97 |
| Cingulum (hippocampus) L | 1.177 | 0.566 | -0.03 | 0.863 |
| Fornix (cres)/Stria terminalis R | 2.922 | 0.09 | -0.27 | 0.113 |
| Fornix (cres)/Stria terminalis L | 2.328 | 0.189 | -0.223 | 0.184 |
| Superior longitudinal fasciculus R | 0.456 | 0.909 | -0.044 | 0.819 |
| Superior longitudinal fasciculus L | 1.423 | 0.477 | -0.197 | 0.247 |
| Superior fronto-occipital fasciculus R | 0.205 | 0.964 | -0.067 | 0.725 |
| Superior fronto-occipital fasciculus L | 2.066 | 0.25 | -0.181 | 0.329 |
| Uncinate fasciculus R | 1.248 | 0.542 | -0.131 | 0.51 |
| Uncinate fasciculus L | 0.54 | 0.862 | -0.072 | 0.704 |
| Tapetum R | 1.652 | 0.399 | -0.151 | 0.428 |
| Tapetum L | 2.459 | 0.162 | -0.235 | 0.16 |

P-values were adjusted for multiple comparisons using Benjamini-Hochberg’s false discovery rate (FDR) correction method.

**Supplemental tables 5a-d:** Permutation test shuffling diagnosis labels in n=5000 permutations. P-value is reported as the proportion of random tests showing more extreme effects than the original analysis. *Abbreviations: ASD = autism spectrum disorder, ED = edge density, FDR = false discovery rate, L = left, R = right*

| **5.a Infants** | | | | |
| --- | --- | --- | --- | --- |
| **tract** | **ED+3** | **ED+5** | **ED-3** | **ED-5** |
| Middle cerebellar peduncle | 0.047 | <0.001 | 0.092 | 0.011 |
| Pontine crossing tract | 0.071 | 0.002 | 0.074 | 0.012 |
| Genu of corpus callosum | 0.028 | 0.078 | 0.027 | 0.025 |
| Body of corpus callosum | 0.006 | 0.005 | 0.019 | 0.083 |
| Splenium of corpus callosum | 0.072 | 0.047 | 0.076 | 0.004 |
| Fornix (column and body of fornix) | 0.092 | <0.001 | 0.007 | 0.096 |
| Corticospinal tract R | 0.052 | <0.001 | 0.053 | 0.008 |
| Corticospinal tract L | 0.075 | <0.001 | 0.097 | 0.035 |
| Medial lemniscus R | 0.010 | <0.001 | 0.051 | 0.039 |
| Medial lemniscus L | 0.014 | 0.005 | 0.067 | 0.069 |
| Inferior cerebellar peduncle R | 0.027 | <0.001 | 0.088 | 0.093 |
| Inferior cerebellar peduncle L | 0.097 | 0.014 | 0.083 | 0.033 |
| Superior cerebellar peduncle R | 0.063 | 0.002 | 0.095 | 0.059 |
| Superior cerebellar peduncle L | 0.063 | <0.001 | 0.090 | 0.081 |
| Cerebral peduncle R | 0.087 | 0.028 | 0.089 | 0.020 |
| Cerebral peduncle L | 0.096 | <0.001 | 0.042 | 0.084 |
| Anterior limb of internal capsule R | 0.099 | 0.075 | 0.072 | 0.015 |
| Anterior limb of internal capsule L | 0.096 | 0.033 | 0.047 | 0.033 |
| Posterior limb of internal capsule R | 0.027 | 0.001 | 0.016 | 0.045 |
| Posterior limb of internal capsule L | 0.005 | <0.001 | 0.009 | 0.088 |
| Retrolenticular part of internal capsule R | 0.020 | <0.001 | 0.004 | 0.035 |
| Retrolenticular part of internal capsule L | 0.083 | 0.003 | 0.079 | 0.033 |
| Anterior corona radiata R | 0.079 | 0.005 | 0.087 | 0.001 |
| Anterior corona radiata L | 0.095 | 0.002 | 0.099 | 0.052 |
| Superior corona radiata R | 0.037 | <0.001 | 0.050 | 0.088 |
| Superior corona radiata L | 0.094 | <0.001 | 0.041 | 0.062 |
| Posterior corona radiata R | 0.051 | <0.001 | 0.058 | 0.010 |
| Posterior corona radiata L | 0.022 | 0.041 | 0.055 | 0.068 |
| Posterior thalamic radiation R | 0.029 | <0.001 | 0.049 | 0.074 |
| Posterior thalamic radiation L | 0.072 | 0.016 | 0.075 | 0.031 |
| Sagittal stratum R | 0.039 | 0.079 | 0.006 | 0.081 |
| Sagittal stratum L | 0.018 | 0.001 | 0.004 | 0.020 |
| External capsule R | 0.026 | <0.001 | 0.048 | 0.003 |
| External capsule L | 0.066 | <0.001 | 0.087 | 0.021 |
| Cingulum (cingulate gyrus) R | 0.088 | <0.001 | 0.028 | 0.063 |
| Cingulum (cingulate gyrus) L | 0.090 | <0.001 | 0.012 | 0.008 |
| Cingulum (hippocampus) R | 0.013 | <0.001 | 0.002 | 0.040 |
| Cingulum (hippocampus) L | 0.006 | <0.001 | 0.031 | 0.050 |
| Fornix (cres)/Stria terminalis R | 0.013 | 0.003 | 0.002 | 0.066 |
| Fornix (cres)/Stria terminalis L | 0.091 | 0.004 | 0.056 | 0.025 |
| Superior longitudinal fasciculus R | 0.089 | <0.001 | 0.032 | 0.099 |
| Superior longitudinal fasciculus L | 0.087 | 0.003 | 0.081 | 0.083 |
| Superior fronto-occipital fasciculus R R | 0.026 | <0.001 | 0.033 | 0.048 |
| Superior fronto-occipital fasciculus R L | 0.049 | <0.001 | 0.048 | 0.021 |
| Inferior fronto-occipital fasciculus R | 0.078 | 0.005 | 0.023 | 0.069 |
| Inferior fronto-occipital fasciculus L | 0.071 | <0.001 | 0.067 | 0.089 |
| Uncinate fasciculus R | 0.085 | 0.011 | 0.040 | 0.090 |
| Uncinate fasciculus L | 0.023 | 0.002 | 0.058 | 0.080 |
| Tapetum R | 0.093 | <0.001 | 0.054 | 0.054 |
| Tapetum L | 0.068 | 0.007 | 0.002 | 0.026 |
| **5.b Toddlers** | | | | |
|  | **ED+3** | **ED+5** | **ED-3** | **ED-5** |
| Middle cerebellar peduncle | 0.071 | 0.006 | 0.093 | 0.044 |
| Pontine crossing tract | 0.071 | 0.002 | 0.096 | 0.082 |
| Genu of corpus callosum | 0.041 | <0.001 | 0.017 | 0.040 |
| Body of corpus callosum | 0.047 | <0.001 | 0.066 | 0.055 |
| Splenium of corpus callosum | 0.044 | 0.004 | 0.038 | 0.010 |
| Fornix (column and body of fornix) | 0.061 | 0.026 | 0.052 | 0.095 |
| Corticospinal tract R | 0.022 | 0.023 | 0.020 | 0.024 |
| Corticospinal tract L | 0.018 | 0.026 | 0.026 | 0.047 |
| Medial lemniscus R | 0.030 | 0.005 | 0.032 | 0.014 |
| Medial lemniscus L | 0.048 | <0.001 | 0.087 | 0.045 |
| Inferior cerebellar peduncle R | 0.016 | 0.022 | 0.031 | 0.065 |
| Inferior cerebellar peduncle L | 0.015 | 0.007 | 0.059 | 0.093 |
| Superior cerebellar peduncle R | 0.009 | 0.015 | 0.015 | 0.008 |
| Superior cerebellar peduncle L | 0.009 | 0.001 | 0.027 | 0.051 |
| Cerebral peduncle R | 0.080 | 0.007 | 0.072 | 0.095 |
| Cerebral peduncle L | 0.043 | 0.014 | 0.083 | 0.086 |
| Anterior limb of internal capsule R | 0.025 | 0.002 | 0.067 | 0.060 |
| Anterior limb of internal capsule L | 0.067 | 0.017 | 0.073 | 0.038 |
| Posterior limb of internal capsule R | 0.093 | 0.001 | 0.081 | 0.086 |
| Posterior limb of internal capsule L | 0.055 | 0.005 | 0.092 | 0.099 |
| Retrolenticular part of internal capsule R | 0.077 | 0.023 | 0.023 | 0.029 |
| Retrolenticular part of internal capsule L | 0.045 | 0.003 | 0.016 | 0.007 |
| Anterior corona radiata R | 0.050 | 0.001 | 0.014 | 0.053 |
| Anterior corona radiata L | 0.067 | 0.001 | 0.017 | 0.034 |
| Superior corona radiata R | 0.093 | 0.002 | 0.084 | 0.088 |
| Superior corona radiata L | 0.099 | <0.001 | 0.079 | 0.041 |
| Posterior corona radiata R | 0.038 | 0.001 | 0.027 | 0.037 |
| Posterior corona radiata L | 0.072 | <0.001 | 0.038 | 0.036 |
| Posterior thalamic radiation R | 0.086 | 0.029 | 0.042 | 0.027 |
| Posterior thalamic radiation L | 0.087 | 0.010 | 0.053 | 0.009 |
| Sagittal stratum R | 0.034 | 0.040 | 0.075 | 0.082 |
| Sagittal stratum L | 0.061 | 0.087 | 0.092 | 0.069 |
| External capsule R | 0.080 | 0.004 | 0.072 | 0.083 |
| External capsule L | 0.022 | 0.002 | 0.011 | 0.001 |
| Cingulum (cingulate gyrus) R | 0.073 | <0.001 | 0.042 | 0.022 |
| Cingulum (cingulate gyrus) L | 0.041 | 0.005 | 0.033 | 0.081 |
| Cingulum (hippocampus) R | 0.061 | 0.047 | 0.084 | 0.091 |
| Cingulum (hippocampus) L | 0.056 | 0.078 | 0.085 | 0.088 |
| Fornix (cres)/Stria terminalis R | 0.075 | 0.040 | 0.040 | 0.023 |
| Fornix (cres)/Stria terminalis L | 0.097 | 0.075 | 0.060 | 0.046 |
| Superior longitudinal fasciculus R | 0.010 | 0.001 | 0.015 | 0.065 |
| Superior longitudinal fasciculus L | 0.023 | <0.001 | 0.007 | 0.045 |
| Superior fronto-occipital fasciculus R R | 0.001 | 0.003 | 0.006 | 0.041 |
| Superior fronto-occipital fasciculus R L | 0.053 | 0.020 | 0.058 | 0.099 |
| Inferior fronto-occipital fasciculus R | 0.041 | 0.016 | 0.097 | 0.089 |
| Inferior fronto-occipital fasciculus L | 0.054 | 0.032 | 0.095 | 0.050 |
| Uncinate fasciculus R | 0.015 | 0.053 | 0.027 | 0.044 |
| Uncinate fasciculus L | 0.053 | 0.061 | 0.058 | 0.066 |
| Tapetum R | 0.087 | 0.020 | 0.075 | 0.056 |
| Tapetum L | 0.003 | 0.001 | 0.004 | 0.005 |
| **5.c Adolescents** | | | | |
|  | **ED+3** | **ED+5** | **ED-3** | **ED-5** |
| Middle cerebellar peduncle | 0.021 | 0.037 | 0.006 | 0.012 |
| Pontine crossing tract | 0.067 | 0.037 | 0.091 | 0.088 |
| Genu of corpus callosum | 0.075 | 0.031 | 0.044 | 0.089 |
| Body of corpus callosum | 0.004 | 0.039 | 0.034 | 0.005 |
| Splenium of corpus callosum | 0.001 | 0.030 | 0.008 | 0.004 |
| Fornix (column and body of fornix) | 0.030 | 0.027 | 0.084 | 0.043 |
| Corticospinal tract R | 0.047 | 0.085 | 0.097 | 0.067 |
| Corticospinal tract L | 0.098 | 0.063 | 0.066 | 0.057 |
| Medial lemniscus R | 0.025 | 0.021 | 0.023 | 0.087 |
| Medial lemniscus L | 0.011 | 0.087 | 0.016 | 0.007 |
| Inferior cerebellar peduncle R | 0.020 | 0.080 | 0.032 | 0.007 |
| Inferior cerebellar peduncle L | 0.080 | 0.075 | 0.034 | 0.067 |
| Superior cerebellar peduncle R | 0.010 | 0.080 | 0.023 | 0.022 |
| Superior cerebellar peduncle L | 0.007 | 0.085 | 0.016 | 0.003 |
| Cerebral peduncle R | 0.008 | 0.050 | 0.051 | 0.099 |
| Cerebral peduncle L | 0.096 | 0.017 | 0.063 | 0.042 |
| Anterior limb of internal capsule R | 0.015 | 0.027 | 0.052 | 0.073 |
| Anterior limb of internal capsule L | 0.011 | 0.084 | 0.080 | 0.085 |
| Posterior limb of internal capsule R | 0.044 | 0.072 | 0.097 | 0.096 |
| Posterior limb of internal capsule L | 0.066 | 0.067 | 0.074 | 0.084 |
| Retrolenticular part of internal capsule R | 0.002 | 0.087 | 0.043 | 0.028 |
| Retrolenticular part of internal capsule L | 0.017 | 0.019 | 0.041 | 0.010 |
| Anterior corona radiata R | 0.003 | 0.034 | 0.026 | 0.028 |
| Anterior corona radiata L | 0.003 | 0.076 | 0.044 | 0.046 |
| Superior corona radiata R | 0.001 | 0.034 | 0.004 | 0.006 |
| Superior corona radiata L | 0.001 | 0.016 | 0.010 | 0.021 |
| Posterior corona radiata R | 0.001 | 0.042 | 0.004 | 0.001 |
| Posterior corona radiata L | <0.001 | 0.009 | 0.002 | <0.001 |
| Posterior thalamic radiation R | 0.001 | 0.039 | 0.008 | 0.027 |
| Posterior thalamic radiation L | 0.002 | 0.003 | 0.026 | 0.009 |
| Sagittal stratum R | 0.001 | 0.037 | 0.027 | 0.041 |
| Sagittal stratum L | 0.001 | 0.001 | 0.015 | 0.004 |
| External capsule R | 0.001 | 0.098 | 0.021 | 0.024 |
| External capsule L | 0.004 | 0.059 | 0.020 | 0.004 |
| Cingulum (cingulate gyrus) R | 0.001 | 0.087 | 0.013 | 0.003 |
| Cingulum (cingulate gyrus) L | 0.001 | 0.005 | 0.024 | 0.020 |
| Cingulum (hippocampus) R | 0.005 | 0.018 | 0.015 | 0.036 |
| Cingulum (hippocampus) L | 0.066 | 0.022 | 0.063 | 0.016 |
| Fornix (cres)/Stria terminalis R | 0.011 | 0.041 | 0.012 | 0.007 |
| Fornix (cres)/Stria terminalis L | 0.008 | 0.009 | 0.038 | 0.026 |
| Superior longitudinal fasciculus R | 0.004 | 0.099 | 0.026 | 0.082 |
| Superior longitudinal fasciculus L | 0.004 | 0.023 | 0.079 | 0.059 |
| Superior fronto-occipital fasciculus R R | 0.001 | 0.077 | 0.002 | 0.026 |
| Superior fronto-occipital fasciculus R L | 0.004 | 0.051 | 0.026 | 0.078 |
| Inferior fronto-occipital fasciculus R | 0.023 | 0.018 | 0.097 | 0.099 |
| Inferior fronto-occipital fasciculus L | 0.020 | 0.056 | 0.084 | 0.032 |
| Uncinate fasciculus R | 0.060 | 0.027 | 0.087 | 0.098 |
| Uncinate fasciculus L | 0.025 | 0.034 | 0.087 | 0.017 |
| Tapetum R | 0.004 | 0.099 | 0.008 | 0.022 |
| Tapetum L | 0.001 | 0.015 | 0.001 | 0.001 |
| **5.d. Adults** | | | | |
|  | **ED+3** | **ED+5** | **ED-3** | **ED-5** |
| Middle cerebellar peduncle | 0.001 | 0.011 | 0.009 | 0.018 |
| Pontine crossing tract | 0.002 | 0.011 | 0.016 | 0.004 |
| Genu of corpus callosum | 0.003 | 0.095 | 0.048 | 0.044 |
| Body of corpus callosum | 0.010 | 0.096 | 0.053 | 0.008 |
| Splenium of corpus callosum | 0.003 | 0.013 | 0.007 | 0.094 |
| Fornix (column and body of fornix) | 0.052 | 0.062 | 0.008 | 0.023 |
| Corticospinal tract R | 0.003 | 0.016 | 0.005 | 0.020 |
| Corticospinal tract L | 0.015 | 0.034 | 0.063 | 0.025 |
| Medial lemniscus R | <0.001 | 0.004 | 0.002 | 0.052 |
| Medial lemniscus L | 0.004 | 0.002 | 0.018 | 0.082 |
| Inferior cerebellar peduncle R | 0.013 | 0.006 | 0.023 | 0.071 |
| Inferior cerebellar peduncle L | 0.060 | 0.064 | 0.099 | 0.035 |
| Superior cerebellar peduncle R | 0.003 | 0.013 | 0.004 | 0.001 |
| Superior cerebellar peduncle L | 0.005 | 0.005 | 0.051 | 0.033 |
| Cerebral peduncle R | 0.001 | 0.041 | <0.001 | 0.031 |
| Cerebral peduncle L | 0.011 | 0.054 | 0.053 | 0.026 |
| Anterior limb of internal capsule R | 0.003 | 0.010 | 0.010 | 0.038 |
| Anterior limb of internal capsule L | 0.047 | 0.016 | 0.042 | 0.030 |
| Posterior limb of internal capsule R | 0.066 | 0.018 | 0.004 | 0.038 |
| Posterior limb of internal capsule L | 0.092 | 0.066 | 0.065 | 0.081 |
| Retrolenticular part of internal capsule R | 0.031 | 0.026 | 0.004 | 0.024 |
| Retrolenticular part of internal capsule L | 0.091 | 0.079 | 0.034 | 0.062 |
| Anterior corona radiata R | 0.028 | 0.005 | 0.060 | 0.058 |
| Anterior corona radiata L | 0.088 | 0.029 | 0.002 | 0.033 |
| Superior corona radiata R | 0.052 | 0.055 | 0.098 | 0.071 |
| Superior corona radiata L | 0.084 | 0.085 | 0.053 | 0.068 |
| Posterior corona radiata R | 0.085 | 0.049 | 0.026 | 0.028 |
| Posterior corona radiata L | 0.017 | 0.078 | 0.075 | 0.004 |
| Posterior thalamic radiation R | 0.066 | 0.010 | 0.021 | 0.053 |
| Posterior thalamic radiation L | 0.086 | 0.019 | 0.071 | 0.027 |
| Sagittal stratum R | 0.012 | 0.036 | 0.011 | 0.081 |
| Sagittal stratum L | 0.028 | 0.060 | 0.010 | 0.007 |
| External capsule R | 0.040 | 0.013 | 0.051 | 0.035 |
| External capsule L | 0.079 | 0.071 | 0.080 | 0.032 |
| Cingulum (cingulate gyrus) R | <0.001 | 0.041 | <0.001 | 0.001 |
| Cingulum (cingulate gyrus) L | 0.006 | 0.047 | 0.069 | 0.033 |
| Cingulum (hippocampus) R | 0.032 | 0.047 | 0.062 | 0.076 |
| Cingulum (hippocampus) L | 0.003 | 0.007 | 0.069 | 0.002 |
| Fornix (cres)/Stria terminalis R | 0.086 | 0.023 | 0.099 | 0.088 |
| Fornix (cres)/Stria terminalis L | 0.055 | 0.097 | 0.067 | 0.012 |
| Superior longitudinal fasciculus R | 0.095 | 0.032 | 0.032 | 0.011 |
| Superior longitudinal fasciculus L | 0.058 | 0.081 | 0.036 | 0.033 |
| Superior fronto-occipital fasciculus R R | 0.098 | 0.011 | 0.013 | 0.076 |
| Superior fronto-occipital fasciculus R L | 0.024 | 0.054 | 0.004 | 0.045 |
| Inferior fronto-occipital fasciculus R | 0.012 | 0.012 | 0.017 | 0.051 |
| Inferior fronto-occipital fasciculus L | 0.096 | 0.099 | 0.092 | 0.052 |
| Uncinate fasciculus R | 0.001 | 0.010 | 0.004 | 0.050 |
| Uncinate fasciculus L | 0.036 | 0.082 | 0.025 | 0.055 |
| Tapetum R | 0.061 | 0.047 | 0.026 | 0.066 |
| Tapetum L | 0.024 | 0.013 | 0.052 | 0.084 |
